# Serum Adiponectin Levels Are Positively Associated With Diabetic Peripheral Neuropathy in Chinese Patients With Type 2 Diabetes

**DOI:** 10.3389/fendo.2020.567959

**Published:** 2020-11-25

**Authors:** Qing Sun, Bin Yan, Dan Yang, Jie Guo, Chao Wang, Qian Zhang, Yue Shi, Xiaohu Shi, Guoqing Tian, Xiaochun Liang

**Affiliations:** ^1^ Department of Traditional Chinese Medicine, Peking Union Medical College Hospital, Chinese Academy of Medical Sciences, Beijing, China; ^2^ Center for Translational Medicine, Peking Union Medical College Hospital, Chinese Academy of Medical Sciences, Beijing, China; ^3^ Department of Integrative Oncology, China-Japan Friendship Hospital, Beijing, China

**Keywords:** adiponectin, diabetic peripheral neuropathy, type 2 diabetes, Chinese patients, biomarker

## Abstract

**Objective:**

To investigate the association between serum adiponectin levels and diabetic peripheral neuropathy (DPN) in Chinese type 2 diabetes (T2D) patients.

**Design and Methods:**

Two hundred nineteen T2D patients aged 40–79 years were divided into two groups according to whether they had DPN. The systemic levels of five biomarkers were measured using a human adipokine multiplexed bead-based immunoassay. Diabetic peripheral neuropathy diagnostic criteria included both common DPN symptoms and neurological screening tests.

**Results:**

Most features of DPN (*n*=98) and non-DPN patients (*n*=121) are similar, but the DPN patients were slightly older, had longer diabetes duration, higher hemoglobin (Hb) A1c, lower estimated glomerular filtration rates (eGFR), less exercise, and used lipid-lowering drugs more often. Serum adiponectin levels of DPN patients were higher than that of non-DPN patients (8.13 *vs*. 9.63 mg/ml, *P* = 0.004). Serum adiponectin levels were positively associated with DPN after adjusting for age, gender, body mass index, hypertension, HbA1c, alcohol intake, smoking status, physical activity, log-transformed low density lipoprotein cholesterol, lipid-lowering drug usage, eGFR, and diabetes duration {odds ratio (OR) 1.72 [95% confidence interval (CI) 1.02-2.89], *P* = 0.041}. The OR refers to a doubling in biomarkers.

**Conclusions:**

Serum adiponectin levels were higher in DPN patients compared to non‑DPN patients in this Chinese T2D population. Serum adiponectin levels were positively associated with DPN presence, independent of multiple confounders.

## Introduction

A 2017 survey by the International Diabetes Federation showed that approximately 425 million people suffer from diabetes mellitus (DM) worldwide, including about 114 million people in China (1). DM can cause a variety of chronic complications, among which diabetic peripheral neuropathy (DPN) is an important cause of disability or death. The study found that, with prolonged disease, DPN prevalence was as high as 30% or more (2). However, DPN pathogenesis remains unclear, thus limiting disease prevention and treatment.

Adiponectin is an adipocytokine that plays many roles in human metabolism, including lipid regulation (3), glucose metabolism, and mediating the bodily response to insulin (4). Unlike DPN in T1D patients, insulin resistance and dyslipidemia are important causes of DPN in T2D patients (5). In type 2 diabetes (T2D) patients, serum adiponectin concentrations have been found to be significantly lower than those non-T2D participants (6), but not as high as those of type 1 diabetic (T1D) patients (7). Previous studies have shown that in diabetic nephropathy (DN) patients, serum adiponectin levels are notably lower than those of non-DN patients; in diabetic retinopathy (DR) patients, serum adiponectin levels are notably higher compared with non-DR patients (8). Higher adiponectin levels are beneficial for autonomic cardiovascular function in T2D patients (9), but are positively correlated with microvascular complications (10). However, the mechanism by which serum adiponectin affects DPN remains controversial due to inconsistencies in reported outcomes (8, 11, 12). Some studies found significantly higher serum adiponectin levels in DPN patients compared to non-DPN patients (11), while others found low serum adiponectin to be significantly associated with DPN incidence (12, 13). Furthermore, one study suggests that adiponectin has no relationship with diabetic distal sensorimotor polyneuropathy (DSPN) (14).

At present, no study has investigated associations between serum adiponectin levels and DPN in Chinese T2D patients. Here, we assess the correlation between adiponectin and DPN in this population using serum adiponectin levels measured in Chinese T2D patients with and without DPN.

## Participants and Methods

### Study Population

This cross-sectional study recruited 246 T2D patients aged 40 to 79 years old who were being treated in-patient at Peking Union Medical College Hospital (PUMCH) between January 2015 and December 2017. Twenty-seven patients were excluded due to missing data in one or more of the study variables (age, sex, body mass index, HbA1c, diabetes duration, hypertension, lipid profile, alcohol intake, smoking, physical activity), resulting in a final sample size of 219 participants. The study was approved by the Institutional Review Boards of PUMCH, Peking Union Medical College, and the Chinese Academy of Medical Sciences (Beijing, China). Written informed consent was obtained from each study participant. Type 2 diabetes was either self‑reported disease that was validated by a medical record review using World Health Organization diagnostic criteria (1999) or determined based on current usage of antidiabetic medications, or fasting and/or 2 h glucose levels in the diabetic range as measured by standardized 75 g oral glucose tolerance test (15).

### Measurement of Serum Biomarkers

Blood samples were collected from participants following overnight fasting. The serum was separated and stored at -80°C until analysis. Serum adiponectin was measured with a human adipokine multiplexed bead-based immunoassay (Millipore, Billerica, MA, USA) on a Luminex^®^ 200™ Bioanalyzer (Austin, TX, USA) that concurrently measured serum leptin, lipocalin-2/NGAL, IL-6, and TNF-α levels. All other measurements were performed using routine laboratory tests and certified methods.

### Assessment of Diabetic Peripheral Neuropathy

Diagnostic criteria for DPN include the presence of common symptoms (foot sensation including pain, numbness, and paresthesia) and neurological screening examinations (temperature and pinprick sensation, 10-g monofilament test, vibration perception with 128-Hz tuning fork, and ankle reflexes). Diabetic peripheral neuropathy was assigned if there was at least one abnormal screening test in patients with DPN symptoms or at least two abnormal screening tests in patients without DPN symptoms (16). Patients with other forms of neuropathy, including chronic inflammatory demyelinating polyneuropathy, infections, malnutrition, exogenous toxins or drugs, hypothyroidism, and renal failure were excluded from the study (5).

### Assessment of Covariates

Participant height, weight, and blood pressure (BP) were measured by standardized methods. Body mass index (BMI) was calculated as kg/m^2^. Hypertension was diagnosed if participant blood pressure was ≥140/90 mmHg or if participant was using antihypertensive medication. Trained medical interviewers collected participant medical history, smoking and alcohol habits, and physical activity. Participants with ≥1 h of sports activity per week during at least one athletic session were classified as physically active. Alcohol intake was defined as none (0 g/day), moderate (0–20 g/day for women, 0–40 g/day for men), or high (≥20 g/day for women, ≥40 g/day for men). Metabolic variables collected from medical records included serum fasting blood glucose, total cholesterol, high-density lipoprotein (HDL) and low-density lipoprotein-cholesterol (LDL-C), triglycerides, uric acid, and plasma hemoglobin A1c (HbA1c). Dyslipidemia was diagnosed in participants who either used lipid-lowering medication or had a total cholesterol level ≥5.70 mmol/L, and/or LDL-C level ≥2.59 mmol/L, and/or HDL-C level ≤ 0.91 mmol/L, and/or triglycerides level ≥1.70 mmol/L (17). Estimated glomerular filtration rate (eGFR) was calculated using the Chronic Kidney Disease Epidemiology Collaboration equation (18). Those potential confounders were selected based on previous literature (12).

### Statistical Analysis

Continuous variables conforming to a normal distribution were presented as mean ± standard deviation (SD), otherwise they were presented in interquartile ranges with a median (25^th^, 75^th^ percentiles). Binary variables were presented as percentages. Differences between DPN and non-DPN participants were analyzed by *t* test, Wilcoxon test, or *χ2* test as appropriate. Associations between adipocytokines and DPN were analyzed separately using logistic regression after adjusting for age and sex, then subsequently adjusting for BMI, hypertension, HbA1c, smoking status, physical activity, and log-transformed LDL-C; data were further adjusted for the use of lipid-lowering medications, eGFR, and diabetes duration. In the sensitivity analyses, we examined the interaction between age groups (<60 *vs.* ≥60), sex, lipid-lowering medications, diabetic retinopathy, diabetic nephropathy and each adipocytokine, by adding appropriate cross-product term in the model. If there were statistically significant interaction effect, we would further conduct stratified analyses. All statistical analyses were performed using SAS 9.3 (SAS Institute, Cary, NC). All *P* values were two-sided and were considered statistically significant if <0.05.

## Results

### Clinical and Biochemical Outcomes

Clinical and biochemical characteristics for the T2D patients in this study are summarized in **Table 1**. Patients with DPN (*n*=98) had higher serum adiponectin and leptin concentrations than those without DPN (*n*=121). Most of the characteristic data were similar between the two groups, although the participants with DPN were slightly older, had a longer diabetes duration, higher HbA1c levels, and lower eGFR levels. Furthermore, they tended to be less physically active and more often used lipid-lowering drugs (**Table 1**).

**Table 1:** Characteristics of Type 2 diabetic participants stratified by DPN status.

Variable	Without DPN(*n* = 121)	With DPN(*n* = 98)	*P* value
Age (years)	58.0 (52.0, 63.0)	62.5 (54.0, 70.0)	**0.001**
Male (%)	48.8	43.9	0.471
Diabetes duration (years)	10 (5, 15)	16 (10, 21)	**＜0.001**
Height (cm)	168 (160, 172)	165 (160, 172)	0.394
BMI (kg/m^2^)	26.3 ± 3.4	26.4 ± 4.3	0.742
Fasting blood glucose (mmol/L)*	2.02 ± 0.31	2.10 ± 0.33	0.052
HbA1c (%)	7.4 (6.7, 8.8)	8.0 (6.7, 9.4)	**0.047**
Hypertension (%)^†^	62.0	67.4	0.410
Systolic BP (mmHg)	135.0 (120.0, 144.0)	139.5 (120.0, 146.0)	0.372
Diastolic BP (mmHg)	78.3 ± 10.3	76.9 ± 11.1	0.317
Dyslipidemia (%)^#^	85.1	86.7	0.734
Total cholesterol (mmol/L)	4.57 (3.73, 5.38)	4.26 (3.58, 5.14)	0.289
Fasting triglycerides (mmol/L)	1.62 (1.08, 2.26)	1.64 (1.13, 2.36)	0.684
HDL-cholesterol (mmol/L)*	0.008 ± 0.26	0.044 ± 0.30	0.345
LDL-cholesterol (mmol/L)*	0.953 ± 0.32	0.866 ± 0.40	0.080
Use of lipid-lowering drugs (%)	28.9	46.9	**0.006**
Uric acid (umol/l)	340.2 ± 82.3	351.8 ± 106.9	0.378
eGFR (ml/min/1.73 m^2^)	93.6 ± 21.0	84.4 ± 30.0	**0.011**
Smoking (%)			0.547
Current	28.9	23.5	
Former	11.6	15.3	
Never	59.5	61.2	
Alcohol intake (%)^‡^			0.064
High	24.8	12.5	
Moderate	4.1	5.1	
None	71.1	82.7	
Physically active (%)^§^	34.7	17.4	**0.004**
Biomarkers			
Adiponectin (mg/ml)	8.13 (5.30, 11.13)	9.63 (6.73, 14.13)	**0.004**
Leptin (ng/ml)	5.87 (2.90, 11.87)	7.94(2.98, 20.13)	**0.048**
NGAL (ng/ml)	103.04(71.55, 200.64)	101.75(79.13, 172.54)	0.944
IL-6 (pg/ml)	7.00 (3.18, 22.82)	7.55 (3.78, 14.88)	0.794
TNF-α (pg/ml)	10.97 (6.31, 25.16)	12.13 (7.16, 17.69)	0.933
hsCRP (mg/L)*	0.19 ± 0.96	0.04 ± 1.10	0.271

Data are given as mean ± SD, median (25^th^, 75^th^ percentiles), or percentages.

BMI, body mass index; DPN, diabetic peripheral neuropathy; HbA1c, hemoglobinA1c; BP, blood pressure; HDL, high-density lipoprotein; LDL, low-density lipoprotein; eGFR, estimated glomerular filtration rate; NAGL, neutrophil gelatinase-associated lipocalin; IL, interleukin; TNF, tumor necrosis factor; hsCRP, high-sensitivity C‑reactive protein; Fbg, fibrinogen.

*Data were log2-transformed before analysis.

^†^Hypertension was assigned if blood pressure ≥140/90 mmHg or use of anti‑hypertensive medication.

^#^Dyslipidemia was diagnosed in participants who used lipid-lowering drugs or had a total cholesterol level ≥5.70 mmol/L, and/or LDL-C level ≥2.59 mmol/L, and/or HDL‑C level ≤1.81 mmol/L, and/or triglycerides level ≥1.70 mmol/L.

^‡^Alcohol intake was classified as none (0 g/day), moderate (≥0 to <20 g/day for women, ≥0 to <40 g/day for men), or high (≥20 g/day for women, ≥40 g/day for men)

^§^Physically active was defined as ≥1 h sports activity per week in at least one athletic season.

Boldface type indicates statistical significance (P<0.05).

### Association Between Serum Adiponectin and Diabetic Peripheral Neuropathy

Correlation between serum adipocytokines and DPN was analyzed using multiple logistic regression analysis (**Table 2**). Results were adjusted for age, gender, BMI, hypertension, HbA1c levels, log-transformed LDL-C levels, alcohol intake, smoking status, and physical activity (models 1–2). The associations between serum adiponectin and leptin levels in DPN patients were statistically significant with an odds ratio (OR) for adiponectin of 1.91, 95% confidence interval (CI): 1.17–3.11, *P*=0.009; OR for leptin: 1.04, 95% CI: 1.00–1.07, *P*=0.040). The positive association with DPN persisted after adjustment for eGFR, diabetes duration, and use of lipid-lowering medications (model 3) for serum adiponectin (OR: 1.72, 95% CI: 1.02–2.89, *P*=0.041), but not for leptin (OR: 1.02, 95% CI: 0.99–1.06, *P*=0.220). In the sensitivity analyses, there were no statistically significant multiplicative interaction between age groups (<60 vs. ≥60), sex, lipid-lowering medications, diabetic retinopathy, diabetic nephropathy, and each adipocytokine (*P* value for interaction >0.05 for all).

**Table 2:** OR and 95% CI for the association between biomarkers and DPN.

Variable	Model 1		Model 2		Model 3	
	OR (95% CI)	*P* value	OR (95% CI)	*P* value	OR (95% CI)	*P* value
Adiponectin	**1.60 (1.06–2.43)**	**0.026**	**1.91 (1.17–3.11)**	**0.009**	**1.72 (1.02–2.89)**	**0.041**
Leptin	**1.04 (1.01–1.07)**	**0.007**	**1.04 (1.00–1.07)**	**0.040**	1.02 (0.99–1.06)	0.220
NGAL	1.06 (0.71–1.60)	0.778	1.08 (0.69–1.67)	0.739	0.90 (0.56–1.45)	0.670
IL-6	0.95 (0.83–1.09)	0.493	0.97 (0.84–1.12)	0.658	0.98 (0.84–1.15)	0.831
TNF-α	0.92 (0.71–1.19)	0.526	0.88 (0.66–1.16)	0.349	0.83 (0.61–1.11)	0.212
hsCRP	0.89 (0.68–1.17)	0.405	**0.69 (0.50–0.97)**	**0.030**	**0.62 (0.43–0.89)**	**0.009**

ORs, 95%CIs, and corresponding P values are given for a doubling in circulating of biomarkers.

Data were log2-transformed before logistic regression except for leptin. Serum adipocytokines were included in the models separately. OR was for per 1-unit increase in corresponding variables.

Model 1: adjusted by age and gender. Model 2: adjusted by model 1 variables plus BMI, hypertension, HbA1c level, alcohol intake, smoking status, physical activity, and log-transformed LDL-C level. Model 3: adjusted by model 2 variables plus use of lipid-lowering medications, eGFR, and disease duration.

Boldface type indicates statistical significance (P < 0.05).

## Discussion

This study found a positive association between serum adiponectin levels and the presence of DPN in T2D Chinese patients and, after adjusting for potential confounders, that association persisted.

Recently, many studies have investigated the relationship between serum adiponectin levels and DPN. A cross-sectional study from India tested serum adiponectin in 487 T2D patients, and the authors found that adiponectin levels are significantly higher in diabetic patients with neuropathy than in those without (11). The same results appeared in a similar study (8). Jung et al. measured adiponectin and evaluated DPN in 153 diabetic patients, and reported that high serum adiponectin levels are independently associated with a higher incidence of neuropathy. In the aforementioned studies, the diagnosis of neuropathy in the former was based on only one detection method, a biothesiometer to assess vibratory perception threshold (VPT). In contrast, the assessment of peripheral neuropathy of the latter was similar to this study, and was evaluated based on typical symptoms and neurological tests. Besides, the participants in these three studies were all Asians and had certain similarities in demographic information.

However, the KORA F4/FF4 study found that low serum adiponectin levels were related to DPN incidence (12). This difference with the results of the present study may be attributable to the following factors. KORA F4/FF4 was a population-based cohort study, with a DPN incidence of 133 DPN and 397 participants with no incidence of DPN, and investigated an elderly population aged 62 to 81 years. Our study is a cross-sectional study of an in-patient population with a relatively small sample size (*n*=219), however the age ranged from 40 to 79 years old. The ethnic compositions of participants in these two studies were significantly different, and adiponectin concentration levels were affected by different genetic backgrounds, potentially leading to different findings based on ethnic differences (19). In addition, the KORA F4/FF4 study used the examination part of the Michigan Neuropathy Screening Instrument (MNSI) and a 10-g monofilament as DPN diagnostic criteria, while our diagnostic criteria utilized common DPN symptoms and neurological screening tests. These confounding factors may have led to the different results observed in the two studies.

In addition, there were studies that differ from the above-mentioned research conclusions. A cross-sectional study from Japan analyzed the relationship between nerve conduction velocity (NCV) and plasma adipocytokines (TNFα, adiponectin, and leptin) in 105 T2D patients (20). The authors reported that there was no significant relationship between plasma adiponectin and NCV. Another study (21), also from Japan, showed that neither serum total nor high molecular weight adiponectin was correlated with DPN in 198 diabetic subjects. The inconsistency between the current research and the previous researches may be due to small sample sizes, If the sample size is appropriately expanded, perhaps the differences will be significant. What’s more, these two studies diagnosed DPN by NCV or bilateral ankle reflex, leading to more advanced DPN patients, whose inflammation was less pronounced (22).

Adiponectin has multiple physiological functions in the human body. Adiponectin can improve insulin resistance induced by a high-fat diet, suggesting that adiponectin may have an insulin-sensitizing effect (23) that may be achieved by activating the AMP-activated protein kinase signaling pathway to promote glucose and fatty acid utilization (24). Another study found that adiponectin may be independent of insulin level, inhibiting glucose production, and promoting glucose assimilation (25). Thus, high concentrations of adiponectin are beneficial for patients with T2D. Adiponectin is the most important anti-inflammatory adipocytokine; it has been found to inhibit the NF-κB-signaling pathway in endothelial cells (26) and macrophages (27), and is involved in the transformation of macrophages from pro-inflammatory M1 to anti-inflammatory M2 cells (28). Adiponectin may alleviate the symptoms of DPN by inhibiting p38 mitogen-activated protein kinase (p38 MAPK) activation, as well as the transient receptor potential cation channel subfamily V member 1 (TRPV1) and calcitonin gene-related peptide (CGRP) signal pathways in dorsal root ganglions neurons (29). Some studies indicate that high concentrations of leptin, the first adipocytokine to be identified, positively correlate with insulin resistance, T2D, and cardiovascular diseases (30). Leptin can upregulate pro-inflammatory cytokines, such as TNF-α and IL-6, while adiponectin has anti‑inflammatory properties. However, the specific mechanism of adiponectin in DPN remains unknown. Adiponectin may play no role in the pathophysiology of DPN, and may be increased to counteract deleterious effects of pro-inflammatory cytokines, thus representing an indirect risk marker. It may also have a novel effect on DPN (31, 32).Strengths of this study are as follows: we explore the association between serum adiponectin levels and DPN prevalence in Chinese T2D patients. Additionally, our data allowed us to control multiple potential confounding variables between serum adiponectin levels and DPN. We used multivariate analyses to account for DPN risk factors as potential confounders.

However, our study has some limitations. First, the cross-sectional observational design may limit findings. Second, the statistical power of our conclusions is low, calling into question whether non-significance was due to a lack of relationship between the groups or due to lack of statistical power. Therefore, results should be interpreted with caution. Large-scale prospective trials are needed to properly evaluate the role of adiponectin in DPN.

## Conclusion

Our results show that serum adiponectin levels in DPN participants were higher than those of non-DPN participants in a Chinese T2D population. Serum adiponectin levels were positively associated with DPN, independent of multiple potential confounders. Future prospective studies are needed to evaluate whether adiponectin can be used as a biomarker and therapeutic target for DPN.

## Data Availability Statement

The raw data supporting the conclusions of this article will be made available by the authors, without undue reservation.

## Ethics Statement

The studies involving human participants were reviewed and approved by Institutional Review Boards of PUMCH, Peking Union Medical College, and the Chinese Academy of Medical Sciences (Beijing, China). The patients/participants provided their written informed consent to participate in this study.

## Author Contributions

XL and GT contributed to the concept and design of the study and supervised this research. QS, DY, CW, QZ, XS, and YS performed the clinical study. QS interpreted the data, drafted part of the manuscript and finally approved the submission of this research. BY wrote part of the manuscript and submitted the manuscript. JG conducted statistical analysis. GT and XL are responsible for the overall contents. All authors contributed to the article and approved the submitted version.

## Funding

This study was supported in part by grants from National Natural Science Foundation of China (grant number 81603481) and the PUMCH Science Fund for Junior Faculty (grant number pumch‑2013‑140).

## Conflict of Interest

The authors declare that the research was conducted in the absence of any commercial or financial relationships that could be construed as a potential conflict of interest.
