# Supplementary material for: Serum Adiponectin Levels Are Positively Associated With Diabetic Peripheral Neuropathy in Chinese Patients With Type 2 Diabetes
Source: Front Endocrinol (Lausanne). 2020 Nov 25;11:567959. doi: 10.3389/fendo.2020.567959 (PMC7724032; doi:10.3389/fendo.2020.567959)
Supplement: Supplementary file 1 [file DataSheet_1.pdf]

model1to3

The LOGISTIC Procedure

Model Information	
Data Set	SASUSER.SUN_30DIA1
Response Variable	dspn_y
Number of Response Levels	2
Model	binary logit
Optimization Technique	Fisher's scoring

Number of Observations Read	219
Number of Observations Used	219

Response Profile		
Ordered Value	dspn_y	Total Frequency
1	0	121
2	1	98

Probability modeled is dspn_y=1.

Class Level Information			
Class	Value	Design Variables	
sex	1	1	0
	2	0	1

Model Convergence Status
Convergence criterion (GCONV=1E-8) satisfied.

Model Fit Statistics		
Criterion	Intercept Only	Intercept and Covariates
AIC	303.178	293.863
SC	306.568	307.419
-2 Log L	301.178	285.863

model1 for logadi

The LOGISTIC Procedure

Testing Global Null Hypothesis: BETA=0			
Test	Chi-Square	DF	Pr > ChiSq
Likelihood Ratio	15.3157	3	0.0016
Score	14.5892	3	0.0022
Wald	13.1723	3	0.0043

Type 3 Analysis of Effects			
Effect	DF	Wald Chi-Square	Pr > ChiSq
logadi	1	4.9853	0.0256
age	1	5.7434	0.0166
sex	1	0.2145	0.6433

Analysis of Maximum Likelihood Estimates						
Parameter		DF	Estimate	Standard Error	Wald Chi-Square	Pr > ChiSq
Intercept		1	-3.4545	0.9923	12.1189	0.0005
logadi		1	0.4727	0.2117	4.9853	0.0256
age		1	0.0360	0.0150	5.7434	0.0166
sex	1	1	0.1367	0.2951	0.2145	0.6433
sex	2	0	0	.	.	.

Odds Ratio Estimates			
Effect	Point Estimate	95% Wald Confidence Limits	
logadi	1.604	1.059	2.430
age	1.037	1.007	1.068
sex 1 vs 2	1.146	0.643	2.044

Association of Predicted Probabilities and Observed Responses			
Percent Concordant	64.5	Somers' D	0.294
Percent Discordant	35.2	Gamma	0.295
Percent Tied	0.3	Tau-a	0.146
Pairs	11858	c	0.647

model1 for logIL6

The LOGISTIC Procedure

Model Information	
**Data Set**	SASUSER.SUN_30DIA1
**Response Variable**	dspn_y
**Number of Response Levels**	2
**Model**	binary logit
**Optimization Technique**	Fisher's scoring

**Number of Observations Read**	219
**Number of Observations Used**	219

Response Profile		
**Ordered Value**	**dspn_y**	**Total Frequency**
**1**	0	121
**2**	1	98

Probability modeled is dspn_y=1.

Class Level Information			
**Class**	**Value**	**Design Variables**	
**sex**	**1**	1	0
	**2**	0	1

Model Convergence Status
Convergence criterion (GCONV=1E-8) satisfied.

Model Fit Statistics		
**Criterion**	**Intercept Only**	**Intercept and Covariates**
**AIC**	303.178	298.999
**SC**	306.568	312.555
**-2 Log L**	301.178	290.999

Testing Global Null Hypothesis: BETA=0			
**Test**	**Chi-Square**	**DF**	**Pr > ChiSq**
**Likelihood Ratio**	10.1799	3	0.0171
**Score**	9.8878	3	0.0195
**Wald**	9.4775	3	0.0236

The LOGISTIC Procedure

Type 3 Analysis of Effects			
Effect	DF	Wald Chi-Square	Pr > ChiSq
logIL6	1	0.4709	0.4926
age	1	8.4611	0.0036
sex	1	0.0026	0.9591

Analysis of Maximum Likelihood Estimates						
Parameter		DF	Estimate	Standard Error	Wald Chi-Square	Pr > ChiSq
Intercept		1	-2.6286	0.9436	7.7604	0.0053
logIL6		1	-0.0472	0.0687	0.4709	0.4926
age		1	0.0429	0.0147	8.4611	0.0036
sex	1	1	-0.0146	0.2850	0.0026	0.9591
sex	2	0	0	.	.	.

Odds Ratio Estimates			
Effect	Point Estimate	95% Wald Confidence Limits	
logIL6	0.954	0.834	1.092
age	1.044	1.014	1.074
sex 1 vs 2	0.985	0.564	1.723

Association of Predicted Probabilities and Observed Responses			
Percent Concordant	62.6	Somers' D	0.257
Percent Discordant	36.9	Gamma	0.258
Percent Tied	0.5	Tau-a	0.128
Pairs	11858	c	0.628

The LOGISTIC Procedure

Model Information	
Data Set	SASUSER.SUN_30DIA1
Response Variable	dspn_y
Number of Response Levels	2
Model	binary logit
Optimization Technique	Fisher's scoring

model1 for logIL1beta

The LOGISTIC Procedure

Number of Observations Read	219
Number of Observations Used	219

Response Profile		
Ordered Value	dspn_y	Total Frequency
1	0	121
2	1	98

Probability modeled is dspn_y=1.

Class Level Information			
Class	Value	Design Variables	
sex	1	1	0
	2	0	1

Model Convergence Status
Convergence criterion (GCONV=1E-8) satisfied.

Model Fit Statistics		
Criterion	Intercept Only	Intercept and Covariates
AIC	303.178	294.050
SC	306.568	307.606
-2 Log L	301.178	286.050

Testing Global Null Hypothesis: BETA=0			
Test	Chi-Square	DF	Pr > ChiSq
Likelihood Ratio	15.1283	3	0.0017
Score	14.1682	3	0.0027
Wald	12.9646	3	0.0047

Type 3 Analysis of Effects			
Effect	DF	Wald Chi-Square	Pr > ChiSq
logIL1beta	1	4.7851	0.0287
age	1	7.2525	0.0071
sex	1	0.0152	0.9020

The LOGISTIC Procedure

Analysis of Maximum Likelihood Estimates						
**Parameter**		**DF**	**Estimate**	**Standard Error**	**Wald Chi-Square**	**Pr > ChiSq**
**Intercept**		1	-2.4183	0.9395	6.6250	0.0101
**logIL1beta**		1	-0.2480	0.1134	4.7851	0.0287
**age**		1	0.0401	0.0149	7.2525	0.0071
**sex**	**1**	1	-0.0354	0.2876	0.0152	0.9020
**sex**	**2**	0	0	.	.	.

Odds Ratio Estimates			
**Effect**	**Point Estimate**	**95% Wald Confidence Limits**	
**logIL1beta**	0.780	0.625	0.975
**age**	1.041	1.011	1.072
**sex 1 vs 2**	0.965	0.549	1.696

Association of Predicted Probabilities and Observed Responses			
**Percent Concordant**	66.3	**Somers' D**	0.329
**Percent Discordant**	33.3	**Gamma**	0.331
**Percent Tied**	0.4	**Tau-a**	0.164
**Pairs**	11858	**c**	0.665

The LOGISTIC Procedure

Model Information	
**Data Set**	SASUSER.SUN_30DIA1
**Response Variable**	dspn_y
**Number of Response Levels**	2
**Model**	binary logit
**Optimization Technique**	Fisher's scoring

**Number of Observations Read**	219
**Number of Observations Used**	219

Response Profile		
**Ordered Value**	**dspn_y**	**Total Frequency**
**1**	0	121
**2**	1	98

The LOGISTIC Procedure

Probability modeled is dspn_y=1.

Class Level Information			
Class	Value	Design Variables	
sex	1	1	0
	2	0	1

Model Convergence Status
Convergence criterion (GCONV=1E-8) satisfied.

Model Fit Statistics		
Criterion	Intercept Only	Intercept and Covariates
AIC	303.178	291.324
SC	306.568	304.880
-2 Log L	301.178	283.324

Testing Global Null Hypothesis: BETA=0			
Test	Chi-Square	DF	Pr > ChiSq
Likelihood Ratio	17.8547	3	0.0005
Score	16.9610	3	0.0007
Wald	14.9488	3	0.0019

Type 3 Analysis of Effects			
Effect	DF	Wald Chi-Square	Pr > ChiSq
Leptin	1	7.1845	0.0074
age	1	7.7590	0.0053
sex	1	1.4891	0.2224

Analysis of Maximum Likelihood Estimates						
Parameter		DF	Estimate	Standard Error	Wald Chi-Square	Pr > ChiSq
Intercept		1	-3.2770	0.9647	11.5386	0.0007
Leptin		1	0.0365	0.0136	7.1845	0.0074
age		1	0.0416	0.0149	7.7590	0.0053
sex	1	1	0.3971	0.3254	1.4891	0.2224
sex	2	0	0	.	.	.

The LOGISTIC Procedure

Odds Ratio Estimates			
Effect	Point Estimate	95% Wald Confidence Limits	
Leptin	1.037	1.010	1.065
age	1.043	1.012	1.073
sex 1 vs 2	1.487	0.786	2.815

Association of Predicted Probabilities and Observed Responses			
Percent Concordant	66.8	Somers' D	0.339
Percent Discordant	32.8	Gamma	0.341
Percent Tied	0.4	Tau-a	0.168
Pairs	11858	c	0.670

The LOGISTIC Procedure

Model Information	
Data Set	SASUSER.SUN_30DIA1
Response Variable	dspn_y
Number of Response Levels	2
Model	binary logit
Optimization Technique	Fisher's scoring

Number of Observations Read	219
Number of Observations Used	219

Response Profile		
Ordered Value	dspn_y	Total Frequency
1	0	121
2	1	98

Probability modeled is dspn_y=1.

Class Level Information			
Class	Value	Design Variables	
sex	1	1	0
	2	0	1

Model Convergence Status
Convergence criterion (GCONV=1E-8) satisfied.

The LOGISTIC Procedure

Model Fit Statistics		
Criterion	Intercept Only	Intercept and Covariates
AIC	303.178	299.067
SC	306.568	312.624
-2 Log L	301.178	291.067

Testing Global Null Hypothesis: BETA=0			
Test	Chi-Square	DF	Pr > ChiSq
Likelihood Ratio	10.1110	3	0.0176
Score	9.8366	3	0.0200
Wald	9.4330	3	0.0241

Type 3 Analysis of Effects			
Effect	DF	Wald Chi-Square	Pr > ChiSq
logtnfa	1	0.4029	0.5256
age	1	8.2854	0.0040
sex	1	0.0012	0.9719

Analysis of Maximum Likelihood Estimates						
Parameter		DF	Estimate	Standard Error	Wald Chi-Square	Pr > ChiSq
Intercept		1	-2.5083	1.0041	6.2397	0.0125
logtnfa		1	-0.0846	0.1333	0.4029	0.5256
age		1	0.0425	0.0148	8.2854	0.0040
sex	1	1	-0.0101	0.2852	0.0012	0.9719
sex	2	0	0	.	.	.

Odds Ratio Estimates			
Effect	Point Estimate	95% Wald Confidence Limits	
logtnfa	0.919	0.708	1.193
age	1.043	1.014	1.074
sex 1 vs 2	0.990	0.566	1.731

The LOGISTIC Procedure

Association of Predicted Probabilities and Observed Responses			
**Percent Concordant**	62.5	**Somers' D**	0.255
**Percent Discordant**	37.0	**Gamma**	0.256
**Percent Tied**	0.5	**Tau-a**	0.126
**Pairs**	11858	**c**	0.627

The LOGISTIC Procedure

Model Information	
**Data Set**	SASUSER.SUN_30DIA1
**Response Variable**	dspn_y
**Number of Response Levels**	2
**Model**	binary logit
**Optimization Technique**	Fisher's scoring

**Number of Observations Read**	219
**Number of Observations Used**	219

Response Profile		
**Ordered Value**	**dspn_y**	**Total Frequency**
**1**	0	121
**2**	1	98

Probability modeled is dspn_y=1.

Class Level Information			
**Class**	**Value**	**Design Variables**	
**sex**	**1**	1	0
	**2**	0	1

Model Convergence Status
Convergence criterion (GCONV=1E-8) satisfied.

The LOGISTIC Procedure

Model Fit Statistics		
Criterion	Intercept Only	Intercept and Covariates
AIC	303.178	290.223
SC	306.568	303.779
-2 Log L	301.178	282.223

Testing Global Null Hypothesis: BETA=0			
Test	Chi-Square	DF	Pr > ChiSq
Likelihood Ratio	18.9553	3	0.0003
Score	18.0863	3	0.0004
Wald	16.5509	3	0.0009

Type 3 Analysis of Effects			
Effect	DF	Wald Chi-Square	Pr > ChiSq
logFbg	1	8.6795	0.0032
age	1	8.4475	0.0037
sex	1	0.1549	0.6939

Analysis of Maximum Likelihood Estimates						
Parameter		DF	Estimate	Standard Error	Wald Chi-Square	Pr > ChiSq
Intercept		1	-5.1910	1.2804	16.4359	<.0001
logFbg		1	2.1226	0.7205	8.6795	0.0032
age		1	0.0437	0.0150	8.4475	0.0037
sex	1	1	0.1160	0.2946	0.1549	0.6939
sex	2	0	0	.	.	.

Odds Ratio Estimates			
Effect	Point Estimate	95% Wald Confidence Limits	
logFbg	8.353	2.035	34.284
age	1.045	1.014	1.076
sex 1 vs 2	1.123	0.630	2.001

The LOGISTIC Procedure

Association of Predicted Probabilities and Observed Responses			
**Percent Concordant**	66.7	**Somers' D**	0.339
**Percent Discordant**	32.9	**Gamma**	0.340
**Percent Tied**	0.4	**Tau-a**	0.168
**Pairs**	11858	**c**	0.669

The LOGISTIC Procedure

Model Information	
**Data Set**	SASUSER.SUN_30DIA1
**Response Variable**	dspn_y
**Number of Response Levels**	2
**Model**	binary logit
**Optimization Technique**	Fisher's scoring

**Number of Observations Read**	219
**Number of Observations Used**	219

Response Profile		
**Ordered Value**	**dspn_y**	**Total Frequency**
**1**	0	121
**2**	1	98

Probability modeled is dspn_y=1.

Class Level Information			
**Class**	**Value**	**Design Variables**	
**sex**	**1**	1	0
	**2**	0	1

Model Convergence Status
Convergence criterion (GCONV=1E-8) satisfied.

The LOGISTIC Procedure

Model Fit Statistics		
Criterion	Intercept Only	Intercept and Covariates
AIC	303.178	298.775
SC	306.568	312.331
-2 Log L	301.178	290.775

Testing Global Null Hypothesis: BETA=0			
Test	Chi-Square	DF	Pr > ChiSq
Likelihood Ratio	10.4040	3	0.0154
Score	10.1137	3	0.0176
Wald	9.6966	3	0.0213

Type 3 Analysis of Effects			
Effect	DF	Wald Chi-Square	Pr > ChiSq
loghsCRP	1	0.6943	0.4047
age	1	7.9638	0.0048
sex	1	0.0426	0.8365

Analysis of Maximum Likelihood Estimates						
Parameter		DF	Estimate	Standard Error	Wald Chi-Square	Pr > ChiSq
Intercept		1	-2.6492	0.9329	8.0636	0.0045
loghsCRP		1	-0.1170	0.1404	0.6943	0.4047
age		1	0.0418	0.0148	7.9638	0.0048
sex	1	1	-0.0596	0.2890	0.0426	0.8365
sex	2	0	0	.	.	.

Odds Ratio Estimates			
Effect	Point Estimate	95% Wald Confidence Limits	
loghsCRP	0.890	0.676	1.171
age	1.043	1.013	1.073
sex 1 vs 2	0.942	0.535	1.660

The LOGISTIC Procedure

Association of Predicted Probabilities and Observed Responses			
**Percent Concordant**	63.2	**Somers' D**	0.268
**Percent Discordant**	36.4	**Gamma**	0.269
**Percent Tied**	0.4	**Tau-a**	0.133
**Pairs**	11858	**c**	0.634

The LOGISTIC Procedure

Model Information	
**Data Set**	SASUSER.SUN_30DIA1
**Response Variable**	dspn_y
**Number of Response Levels**	2
**Model**	binary logit
**Optimization Technique**	Fisher's scoring

**Number of Observations Read**	219
**Number of Observations Used**	219

Response Profile		
**Ordered Value**	**dspn_y**	**Total Frequency**
**1**	0	121
**2**	1	98

Probability modeled is dspn_y=1.

Class Level Information			
**Class**	**Value**	**Design Variables**	
**sex**	**1**	1	0
	**2**	0	1

Model Convergence Status
Convergence criterion (GCONV=1E-8) satisfied.

The LOGISTIC Procedure

Model Fit Statistics		
Criterion	Intercept Only	Intercept and Covariates
AIC	303.178	299.394
SC	306.568	312.951
-2 Log L	301.178	291.394

Testing Global Null Hypothesis: BETA=0			
Test	Chi-Square	DF	Pr > ChiSq
Likelihood Ratio	9.7842	3	0.0205
Score	9.5519	3	0.0228
Wald	9.1838	3	0.0269

Type 3 Analysis of Effects			
Effect	DF	Wald Chi-Square	Pr > ChiSq
logl2n	1	0.0795	0.7780
age	1	8.7184	0.0032
sex	1	0.0096	0.9221

Analysis of Maximum Likelihood Estimates						
Parameter		DF	Estimate	Standard Error	Wald Chi-Square	Pr > ChiSq
Intercept		1	-3.0655	1.4131	4.7058	0.0301
logl2n		1	0.0587	0.2082	0.0795	0.7780
age		1	0.0436	0.0148	8.7184	0.0032
sex	1	1	-0.0280	0.2862	0.0096	0.9221
sex	2	0	0	.	.	.

Odds Ratio Estimates			
Effect	Point Estimate	95% Wald Confidence Limits	
logl2n	1.060	0.705	1.595
age	1.045	1.015	1.075
sex 1 vs 2	0.972	0.555	1.704

The LOGISTIC Procedure

Association of Predicted Probabilities and Observed Responses			
**Percent Concordant**	62.3	**Somers' D**	0.251
**Percent Discordant**	37.2	**Gamma**	0.252
**Percent Tied**	0.4	**Tau-a**	0.125
**Pairs**	11858	**c**	0.625

The LOGISTIC Procedure

Model Information	
**Data Set**	SASUSER.SUN_30DIA1
**Response Variable**	dspn_y
**Number of Response Levels**	2
**Model**	binary logit
**Optimization Technique**	Fisher's scoring

**Number of Observations Read**	219
**Number of Observations Used**	219

Response Profile		
**Ordered Value**	**dspn_y**	**Total Frequency**
**1**	0	121
**2**	1	98

Probability modeled is dspn_y=1.

Class Level Information				
**Class**	**Value**	**Design Variables**		
**sex**	**1**	1	0	
	**2**	0	1	
**alcohol**	**0**	1	0	0
	**1**	0	1	0
	**2**	0	0	1
**smoking**	**0**	1	0	0
	**1**	0	1	0
	**2**	0	0	1
**physically_active**	**0**	1	0	
	**1**	0	1	

The LOGISTIC Procedure

Model Convergence Status
Convergence criterion (GCONV=1E-8) satisfied.

Model Fit Statistics		
Criterion	Intercept Only	Intercept and Covariates
AIC	303.178	284.995
SC	306.568	329.053
-2 Log L	301.178	258.995

Testing Global Null Hypothesis: BETA=0			
Test	Chi-Square	DF	Pr > ChiSq
Likelihood Ratio	42.1838	12	<.0001
Score	37.7025	12	0.0002
Wald	30.7553	12	0.0021

Type 3 Analysis of Effects			
Effect	DF	Wald Chi-Square	Pr > ChiSq
logadi	1	6.7680	0.0093
age	1	6.0640	0.0138
sex	1	1.6472	0.1993
BMI	1	0.0663	0.7968
hypertension	1	0.1946	0.6591
logLDL_C	1	6.0745	0.0137
HbA1c	1	6.9964	0.0082
alcohol	2	3.6329	0.1626
smoking	2	0.4253	0.8085
physically_active	1	8.1831	0.0042

Analysis of Maximum Likelihood Estimates						
Parameter		DF	Estimate	Standard Error	Wald Chi-Square	Pr > ChiSq
Intercept		1	-7.3402	2.1129	12.0689	0.0005
logadi		1	0.6462	0.2484	6.7680	0.0093
age		1	0.0451	0.0183	6.0640	0.0138
sex	1	1	0.5539	0.4315	1.6472	0.1993
sex	2	0	0	.	.	.

The LOGISTIC Procedure

Analysis of Maximum Likelihood Estimates						
**Parameter**		**DF**	**Estimate**	**Standard Error**	**Wald Chi-Square**	**Pr > ChiSq**
**BMI**		1	0.0111	0.0433	0.0663	0.7968
**hypertension**		1	0.1545	0.3503	0.1946	0.6591
**logLDL_C**		1	-1.1016	0.4470	6.0745	0.0137
**HbA1c**		1	0.2544	0.0962	6.9964	0.0082
**alcohol**	**0**	1	0.9290	0.4880	3.6243	0.0569
**alcohol**	**1**	1	0.6222	0.7737	0.6468	0.4213
**alcohol**	**2**	0	0	.	.	.
**smoking**	**0**	1	-0.1873	0.4617	0.1646	0.6850
**smoking**	**1**	1	-0.3651	0.5668	0.4148	0.5195
**smoking**	**2**	0	0	.	.	.
**physically_active**	**0**	1	1.0505	0.3672	8.1831	0.0042
**physically_active**	**1**	0	0	.	.	.

Odds Ratio Estimates			
**Effect**	**Point Estimate**	**95% Wald Confidence Limits**	
**logadi**	1.908	1.173	3.105
**age**	1.046	1.009	1.084
**sex 1 vs 2**	1.740	0.747	4.054
**BMI**	1.011	0.929	1.101
**hypertension**	1.167	0.587	2.319
**logLDL_C**	0.332	0.138	0.798
**HbA1c**	1.290	1.068	1.557
**alcohol 0 vs 2**	2.532	0.973	6.589
**alcohol 1 vs 2**	1.863	0.409	8.489
**smoking 0 vs 2**	0.829	0.335	2.049
**smoking 1 vs 2**	0.694	0.229	2.108
**physically_active 0 vs 1**	2.859	1.392	5.872

Association of Predicted Probabilities and Observed Responses			
**Percent Concordant**	75.0	**Somers' D**	0.501
**Percent Discordant**	24.8	**Gamma**	0.502
**Percent Tied**	0.2	**Tau-a**	0.249
**Pairs**	11858	**c**	0.751

The LOGISTIC Procedure

Model Information	
**Data Set**	SASUSER.SUN_30DIA1
**Response Variable**	dspn_y
**Number of Response Levels**	2
**Model**	binary logit
**Optimization Technique**	Fisher's scoring

**Number of Observations Read**	219
**Number of Observations Used**	219

Response Profile		
**Ordered Value**	**dspn_y**	**Total Frequency**
**1**	0	121
**2**	1	98

Probability modeled is dspn_y=1.

Class Level Information				
**Class**	**Value**	**Design Variables**		
**sex**	**1**	1	0	
	**2**	0	1	
**alcohol**	**0**	1	0	0
	**1**	0	1	0
	**2**	0	0	1
**smoking**	**0**	1	0	0
	**1**	0	1	0
	**2**	0	0	1
**physically_active**	**0**	1	0	
	**1**	0	1	

Model Convergence Status
Convergence criterion (GCONV=1E-8) satisfied.

The LOGISTIC Procedure

Model Fit Statistics		
Criterion	Intercept Only	Intercept and Covariates
AIC	303.178	292.437
SC	306.568	336.495
-2 Log L	301.178	266.437

Testing Global Null Hypothesis: BETA=0			
Test	Chi-Square	DF	Pr > ChiSq
Likelihood Ratio	34.7417	12	0.0005
Score	32.2880	12	0.0012
Wald	27.7931	12	0.0059

Type 3 Analysis of Effects			
Effect	DF	Wald Chi-Square	Pr > ChiSq
logIL6	1	0.1964	0.6576
age	1	9.2841	0.0023
sex	1	0.4017	0.5262
BMI	1	0.2779	0.5981
hypertension	1	0.0012	0.9725
logLDL_C	1	5.7352	0.0166
HbA1c	1	6.4387	0.0112
alcohol	2	2.2218	0.3293
smoking	2	0.2101	0.9003
physically_active	1	8.2006	0.0042

Analysis of Maximum Likelihood Estimates						
Parameter		DF	Estimate	Standard Error	Wald Chi-Square	Pr > ChiSq
Intercept		1	-6.2267	2.0468	9.2551	0.0023
logIL6		1	-0.0328	0.0740	0.1964	0.6576
age		1	0.0543	0.0178	9.2841	0.0023
sex	1	1	0.2617	0.4129	0.4017	0.5262
sex	2	0	0	.	.	.
BMI		1	0.0224	0.0424	0.2779	0.5981
hypertension		1	0.0118	0.3420	0.0012	0.9725
logLDL_C		1	-1.0539	0.4401	5.7352	0.0166

The LOGISTIC Procedure

Analysis of Maximum Likelihood Estimates						
Parameter		DF	Estimate	Standard Error	Wald Chi-Square	Pr > ChiSq
HbA1c		1	0.2396	0.0944	6.4387	0.0112
alcohol	0	1	0.7095	0.4768	2.2146	0.1367
alcohol	1	1	0.4671	0.7671	0.3709	0.5425
alcohol	2	0	0	.	.	.
smoking	0	1	-0.1545	0.4498	0.1180	0.7313
smoking	1	1	-0.2379	0.5503	0.1870	0.6655
smoking	2	0	0	.	.	.
physically_active	0	1	1.0362	0.3619	8.2006	0.0042
physically_active	1	0	0	.	.	.

Odds Ratio Estimates			
Effect	Point Estimate	95% Wald Confidence Limits	
logIL6	0.968	0.837	1.119
age	1.056	1.020	1.093
sex 1 vs 2	1.299	0.578	2.918
BMI	1.023	0.941	1.111
hypertension	1.012	0.518	1.978
logLDL_C	0.349	0.147	0.826
HbA1c	1.271	1.056	1.529
alcohol 0 vs 2	2.033	0.799	5.176
alcohol 1 vs 2	1.595	0.355	7.175
smoking 0 vs 2	0.857	0.355	2.069
smoking 1 vs 2	0.788	0.268	2.318
physically_active 0 vs 1	2.819	1.387	5.729

Association of Predicted Probabilities and Observed Responses			
Percent Concordant	73.2	Somers' D	0.465
Percent Discordant	26.6	Gamma	0.466
Percent Tied	0.2	Tau-a	0.231
Pairs	11858	c	0.733

The LOGISTIC Procedure

Model Information	
**Data Set**	SASUSER.SUN_30DIA1
**Response Variable**	dspn_y
**Number of Response Levels**	2
**Model**	binary logit
**Optimization Technique**	Fisher's scoring

**Number of Observations Read**	219
**Number of Observations Used**	219

Response Profile		
**Ordered Value**	**dspn_y**	**Total Frequency**
**1**	0	121
**2**	1	98

Probability modeled is dspn_y=1.

Class Level Information				
**Class**	**Value**	**Design Variables**		
**sex**	**1**	1	0	
	**2**	0	1	
**alcohol**	**0**	1	0	0
	**1**	0	1	0
	**2**	0	0	1
**smoking**	**0**	1	0	0
	**1**	0	1	0
	**2**	0	0	1
**physically_active**	**0**	1	0	
	**1**	0	1	

Model Convergence Status
Convergence criterion (GCONV=1E-8) satisfied.

The LOGISTIC Procedure

Model Fit Statistics		
Criterion	Intercept Only	Intercept and Covariates
AIC	303.178	287.738
SC	306.568	331.796
-2 Log L	301.178	261.738

Testing Global Null Hypothesis: BETA=0			
Test	Chi-Square	DF	Pr > ChiSq
Likelihood Ratio	39.4404	12	<.0001
Score	36.0796	12	0.0003
Wald	30.6204	12	0.0023

Type 3 Analysis of Effects			
Effect	DF	Wald Chi-Square	Pr > ChiSq
logIL1beta	1	4.3342	0.0374
age	1	8.2316	0.0041
sex	1	0.3138	0.5754
BMI	1	0.2336	0.6289
hypertension	1	0.0103	0.9191
logLDL_C	1	5.0866	0.0241
HbA1c	1	5.3478	0.0207
alcohol	2	2.4919	0.2877
smoking	2	0.2763	0.8710
physically_active	1	8.5714	0.0034

Analysis of Maximum Likelihood Estimates						
Parameter		DF	Estimate	Standard Error	Wald Chi-Square	Pr > ChiSq
Intercept		1	-5.8721	2.0491	8.2120	0.0042
logIL1beta		1	-0.2535	0.1218	4.3342	0.0374
age		1	0.0517	0.0180	8.2316	0.0041
sex	1	1	0.2334	0.4167	0.3138	0.5754
sex	2	0	0	.	.	.
BMI		1	0.0207	0.0428	0.2336	0.6289
hypertension		1	0.0351	0.3454	0.0103	0.9191
logLDL_C		1	-0.9984	0.4427	5.0866	0.0241

The LOGISTIC Procedure

Analysis of Maximum Likelihood Estimates						
Parameter		DF	Estimate	Standard Error	Wald Chi-Square	Pr > ChiSq
HbA1c		1	0.2206	0.0954	5.3478	0.0207
alcohol	0	1	0.7217	0.4789	2.2710	0.1318
alcohol	1	1	0.7835	0.8168	0.9200	0.3375
alcohol	2	0	0	.	.	.
smoking	0	1	-0.1807	0.4548	0.1579	0.6911
smoking	1	1	-0.2752	0.5573	0.2439	0.6214
smoking	2	0	0	.	.	.
physically_active	0	1	1.0675	0.3646	8.5714	0.0034
physically_active	1	0	0	.	.	.

Odds Ratio Estimates			
Effect	Point Estimate	95% Wald Confidence Limits	
logIL1beta	0.776	0.611	0.985
age	1.053	1.017	1.091
sex 1 vs 2	1.263	0.558	2.858
BMI	1.021	0.939	1.110
hypertension	1.036	0.526	2.038
logLDL_C	0.368	0.155	0.877
HbA1c	1.247	1.034	1.503
alcohol 0 vs 2	2.058	0.805	5.262
alcohol 1 vs 2	2.189	0.442	10.852
smoking 0 vs 2	0.835	0.342	2.035
smoking 1 vs 2	0.759	0.255	2.264
physically_active 0 vs 1	2.908	1.423	5.942

Association of Predicted Probabilities and Observed Responses			
Percent Concordant	74.1	Somers' D	0.484
Percent Discordant	25.7	Gamma	0.485
Percent Tied	0.2	Tau-a	0.241
Pairs	11858	c	0.742

The LOGISTIC Procedure

Model Information	
**Data Set**	SASUSER.SUN_30DIA1
**Response Variable**	dspn_y
**Number of Response Levels**	2
**Model**	binary logit
**Optimization Technique**	Fisher's scoring

**Number of Observations Read**	219
**Number of Observations Used**	219

Response Profile		
**Ordered Value**	**dspn_y**	**Total Frequency**
**1**	0	121
**2**	1	98

Probability modeled is dspn_y=1.

Class Level Information				
**Class**	**Value**	**Design Variables**		
**sex**	**1**	1	0	
	**2**	0	1	
**alcohol**	**0**	1	0	0
	**1**	0	1	0
	**2**	0	0	1
**smoking**	**0**	1	0	0
	**1**	0	1	0
	**2**	0	0	1
**physically_active**	**0**	1	0	
	**1**	0	1	

Model Convergence Status
Convergence criterion (GCONV=1E-8) satisfied.

The LOGISTIC Procedure

Model Fit Statistics		
Criterion	Intercept Only	Intercept and Covariates
AIC	303.178	288.067
SC	306.568	332.125
-2 Log L	301.178	262.067

Testing Global Null Hypothesis: BETA=0			
Test	Chi-Square	DF	Pr > ChiSq
Likelihood Ratio	39.1117	12	0.0001
Score	36.1636	12	0.0003
Wald	30.4832	12	0.0024

Type 3 Analysis of Effects			
Effect	DF	Wald Chi-Square	Pr > ChiSq
Leptin	1	4.2228	0.0399
age	1	7.2923	0.0069
sex	1	2.2247	0.1358
BMI	1	0.3061	0.5801
hypertension	1	0.0009	0.9756
logLDL_C	1	3.6392	0.0564
HbA1c	1	7.2573	0.0071
alcohol	2	1.7205	0.4231
smoking	2	0.2910	0.8646
physically_active	1	7.1327	0.0076

Analysis of Maximum Likelihood Estimates						
Parameter		DF	Estimate	Standard Error	Wald Chi-Square	Pr > ChiSq
Intercept		1	-5.5420	2.0699	7.1683	0.0074
Leptin		1	0.0346	0.0168	4.2228	0.0399
age		1	0.0490	0.0181	7.2923	0.0069
sex	1	1	0.6916	0.4637	2.2247	0.1358
sex	2	0	0	.	.	.
BMI		1	-0.0271	0.0490	0.3061	0.5801
hypertension		1	-0.0104	0.3411	0.0009	0.9756
logLDL_C		1	-0.8628	0.4523	3.6392	0.0564

The LOGISTIC Procedure

Analysis of Maximum Likelihood Estimates						
Parameter		DF	Estimate	Standard Error	Wald Chi-Square	Pr > ChiSq
HbA1c		1	0.2559	0.0950	7.2573	0.0071
alcohol	0	1	0.6269	0.4780	1.7204	0.1896
alcohol	1	1	0.3770	0.7620	0.2447	0.6208
alcohol	2	0	0	.	.	.
smoking	0	1	-0.0148	0.4644	0.0010	0.9746
smoking	1	1	-0.2700	0.5560	0.2358	0.6273
smoking	2	0	0	.	.	.
physically_active	0	1	0.9752	0.3652	7.1327	0.0076
physically_active	1	0	0	.	.	.

Odds Ratio Estimates			
Effect	Point Estimate	95% Wald Confidence Limits	
Leptin	1.035	1.002	1.070
age	1.050	1.014	1.088
sex 1 vs 2	1.997	0.805	4.955
BMI	0.973	0.884	1.071
hypertension	0.990	0.507	1.931
logLDL_C	0.422	0.174	1.024
HbA1c	1.292	1.072	1.556
alcohol 0 vs 2	1.872	0.734	4.777
alcohol 1 vs 2	1.458	0.327	6.491
smoking 0 vs 2	0.985	0.397	2.449
smoking 1 vs 2	0.763	0.257	2.270
physically_active 0 vs 1	2.652	1.296	5.424

Association of Predicted Probabilities and Observed Responses			
Percent Concordant	74.3	Somers' D	0.488
Percent Discordant	25.5	Gamma	0.490
Percent Tied	0.2	Tau-a	0.243
Pairs	11858	c	0.744

The LOGISTIC Procedure

Model Information	
**Data Set**	SASUSER.SUN_30DIA1
**Response Variable**	dspn_y
**Number of Response Levels**	2
**Model**	binary logit
**Optimization Technique**	Fisher's scoring

**Number of Observations Read**	219
**Number of Observations Used**	219

Response Profile		
**Ordered Value**	**dspn_y**	**Total Frequency**
**1**	0	121
**2**	1	98

Probability modeled is dspn_y=1.

Class Level Information				
**Class**	**Value**	**Design Variables**		
**sex**	**1**	1	0	
	**2**	0	1	
**alcohol**	**0**	1	0	0
	**1**	0	1	0
	**2**	0	0	1
**smoking**	**0**	1	0	0
	**1**	0	1	0
	**2**	0	0	1
**physically_active**	**0**	1	0	
	**1**	0	1	

Model Convergence Status
Convergence criterion (GCONV=1E-8) satisfied.

The LOGISTIC Procedure

Model Fit Statistics		
Criterion	Intercept Only	Intercept and Covariates
AIC	303.178	291.745
SC	306.568	335.803
-2 Log L	301.178	265.745

Testing Global Null Hypothesis: BETA=0			
Test	Chi-Square	DF	Pr > ChiSq
Likelihood Ratio	35.4336	12	0.0004
Score	32.8793	12	0.0010
Wald	28.2236	12	0.0051

Type 3 Analysis of Effects			
Effect	DF	Wald Chi-Square	Pr > ChiSq
logtnfa	1	0.8760	0.3493
age	1	9.1150	0.0025
sex	1	0.4528	0.5010
BMI	1	0.3024	0.5824
hypertension	1	0.0026	0.9590
logLDL_C	1	6.0803	0.0137
HbA1c	1	6.6550	0.0099
alcohol	2	2.3791	0.3044
smoking	2	0.2753	0.8714
physically_active	1	8.2534	0.0041

Analysis of Maximum Likelihood Estimates						
Parameter		DF	Estimate	Standard Error	Wald Chi-Square	Pr > ChiSq
Intercept		1	-5.9880	2.0673	8.3900	0.0038
logtnfa		1	-0.1335	0.1426	0.8760	0.3493
age		1	0.0539	0.0179	9.1150	0.0025
sex	1	1	0.2784	0.4138	0.4528	0.5010
sex	2	0	0	.	.	.
BMI		1	0.0233	0.0424	0.3024	0.5824
hypertension		1	0.0176	0.3420	0.0026	0.9590
logLDL_C		1	-1.0838	0.4395	6.0803	0.0137

The LOGISTIC Procedure

Analysis of Maximum Likelihood Estimates						
Parameter		DF	Estimate	Standard Error	Wald Chi-Square	Pr > ChiSq
HbA1c		1	0.2439	0.0945	6.6550	0.0099
alcohol	0	1	0.7329	0.4754	2.3762	0.1232
alcohol	1	1	0.4730	0.7738	0.3736	0.5411
alcohol	2	0	0	.	.	.
smoking	0	1	-0.1794	0.4517	0.1577	0.6913
smoking	1	1	-0.2725	0.5522	0.2436	0.6216
smoking	2	0	0	.	.	.
physically_active	0	1	1.0401	0.3621	8.2534	0.0041
physically_active	1	0	0	.	.	.

Odds Ratio Estimates			
Effect	Point Estimate	95% Wald Confidence Limits	
logtnfa	0.875	0.662	1.157
age	1.055	1.019	1.093
sex 1 vs 2	1.321	0.587	2.973
BMI	1.024	0.942	1.112
hypertension	1.018	0.521	1.990
logLDL_C	0.338	0.143	0.801
HbA1c	1.276	1.060	1.536
alcohol 0 vs 2	2.081	0.820	5.284
alcohol 1 vs 2	1.605	0.352	7.313
smoking 0 vs 2	0.836	0.345	2.026
smoking 1 vs 2	0.761	0.258	2.247
physically_active 0 vs 1	2.830	1.392	5.753

Association of Predicted Probabilities and Observed Responses			
Percent Concordant	73.5	Somers' D	0.472
Percent Discordant	26.3	Gamma	0.473
Percent Tied	0.2	Tau-a	0.235
Pairs	11858	c	0.736

The LOGISTIC Procedure

Model Information	
**Data Set**	SASUSER.SUN_30DIA1
**Response Variable**	dspn_y
**Number of Response Levels**	2
**Model**	binary logit
**Optimization Technique**	Fisher's scoring

**Number of Observations Read**	219
**Number of Observations Used**	219

Response Profile		
**Ordered Value**	**dspn_y**	**Total Frequency**
**1**	0	121
**2**	1	98

Probability modeled is dspn_y=1.

Class Level Information				
**Class**	**Value**	**Design Variables**		
**sex**	**1**	1	0	
	**2**	0	1	
**alcohol**	**0**	1	0	0
	**1**	0	1	0
	**2**	0	0	1
**smoking**	**0**	1	0	0
	**1**	0	1	0
	**2**	0	0	1
**physically_active**	**0**	1	0	
	**1**	0	1	

Model Convergence Status
Convergence criterion (GCONV=1E-8) satisfied.

The LOGISTIC Procedure

Model Fit Statistics		
Criterion	Intercept Only	Intercept and Covariates
AIC	303.178	284.058
SC	306.568	328.116
-2 Log L	301.178	258.058

Testing Global Null Hypothesis: BETA=0			
Test	Chi-Square	DF	Pr > ChiSq
Likelihood Ratio	43.1209	12	<.0001
Score	39.6567	12	<.0001
Wald	33.2934	12	0.0009

Type 3 Analysis of Effects			
Effect	DF	Wald Chi-Square	Pr > ChiSq
logFbg	1	7.9899	0.0047
age	1	9.0515	0.0026
sex	1	1.1563	0.2822
BMI	1	0.0090	0.9245
hypertension	1	0.0199	0.8878
logLDL_C	1	6.8419	0.0089
HbA1c	1	5.4631	0.0194
alcohol	2	1.6381	0.4409
smoking	2	0.4319	0.8058
physically_active	1	9.1471	0.0025

Analysis of Maximum Likelihood Estimates						
Parameter		DF	Estimate	Standard Error	Wald Chi-Square	Pr > ChiSq
Intercept		1	-7.9940	2.1648	13.6362	0.0002
logFbg		1	2.2257	0.7874	7.9899	0.0047
age		1	0.0552	0.0184	9.0515	0.0026
sex	1	1	0.4629	0.4305	1.1563	0.2822
sex	2	0	0	.	.	.
BMI		1	-0.00419	0.0442	0.0090	0.9245
hypertension		1	-0.0492	0.3484	0.0199	0.8878
logLDL_C		1	-1.1778	0.4503	6.8419	0.0089

The LOGISTIC Procedure

Analysis of Maximum Likelihood Estimates						
Parameter		DF	Estimate	Standard Error	Wald Chi-Square	Pr > ChiSq
HbA1c		1	0.2290	0.0980	5.4631	0.0194
alcohol	0	1	0.6119	0.4887	1.5681	0.2105
alcohol	1	1	0.1823	0.7817	0.0544	0.8156
alcohol	2	0	0	.	.	.
smoking	0	1	-0.0291	0.4625	0.0040	0.9499
smoking	1	1	-0.3422	0.5661	0.3655	0.5455
smoking	2	0	0	.	.	.
physically_active	0	1	1.1170	0.3693	9.1471	0.0025
physically_active	1	0	0	.	.	.

Odds Ratio Estimates			
Effect	Point Estimate	95% Wald Confidence Limits	
logFbg	9.260	1.979	43.338
age	1.057	1.019	1.096
sex 1 vs 2	1.589	0.683	3.694
BMI	0.996	0.913	1.086
hypertension	0.952	0.481	1.884
logLDL_C	0.308	0.127	0.744
HbA1c	1.257	1.038	1.524
alcohol 0 vs 2	1.844	0.708	4.805
alcohol 1 vs 2	1.200	0.259	5.554
smoking 0 vs 2	0.971	0.392	2.405
smoking 1 vs 2	0.710	0.234	2.154
physically_active 0 vs 1	3.056	1.482	6.302

Association of Predicted Probabilities and Observed Responses			
Percent Concordant	75.3	Somers' D	0.509
Percent Discordant	24.5	Gamma	0.510
Percent Tied	0.2	Tau-a	0.253
Pairs	11858	c	0.754

The LOGISTIC Procedure

Model Information	
**Data Set**	SASUSER.SUN_30DIA1
**Response Variable**	dspn_y
**Number of Response Levels**	2
**Model**	binary logit
**Optimization Technique**	Fisher's scoring

**Number of Observations Read**	219
**Number of Observations Used**	219

Response Profile		
**Ordered Value**	**dspn_y**	**Total Frequency**
**1**	0	121
**2**	1	98

Probability modeled is dspn_y=1.

Class Level Information				
**Class**	**Value**	**Design Variables**		
**sex**	**1**	1	0	
	**2**	0	1	
**alcohol**	**0**	1	0	0
	**1**	0	1	0
	**2**	0	0	1
**smoking**	**0**	1	0	0
	**1**	0	1	0
	**2**	0	0	1
**physically_active**	**0**	1	0	
	**1**	0	1	

Model Convergence Status
Convergence criterion (GCONV=1E-8) satisfied.

The LOGISTIC Procedure

Model Fit Statistics		
Criterion	Intercept Only	Intercept and Covariates
AIC	303.178	287.788
SC	306.568	331.846
-2 Log L	301.178	261.788

Testing Global Null Hypothesis: BETA=0			
Test	Chi-Square	DF	Pr > ChiSq
Likelihood Ratio	39.3901	12	<.0001
Score	36.0710	12	0.0003
Wald	30.2247	12	0.0026

Type 3 Analysis of Effects			
Effect	DF	Wald Chi-Square	Pr > ChiSq
loghsCRP	1	4.6880	0.0304
age	1	8.6927	0.0032
sex	1	0.2728	0.6014
BMI	1	1.0239	0.3116
hypertension	1	0.2528	0.6151
logLDL_C	1	5.0041	0.0253
HbA1c	1	8.6424	0.0033
alcohol	2	2.8744	0.2376
smoking	2	0.3611	0.8348
physically_active	1	9.3913	0.0022

Analysis of Maximum Likelihood Estimates						
Parameter		DF	Estimate	Standard Error	Wald Chi-Square	Pr > ChiSq
Intercept		1	-7.4923	2.1357	12.3069	0.0005
loghsCRP		1	-0.3666	0.1693	4.6880	0.0304
age		1	0.0530	0.0180	8.6927	0.0032
sex	1	1	0.2171	0.4157	0.2728	0.6014
sex	2	0	0	.	.	.
BMI		1	0.0447	0.0442	1.0239	0.3116
hypertension		1	0.1788	0.3556	0.2528	0.6151
logLDL_C		1	-0.9878	0.4416	5.0041	0.0253

The LOGISTIC Procedure

Analysis of Maximum Likelihood Estimates						
Parameter		DF	Estimate	Standard Error	Wald Chi-Square	Pr > ChiSq
HbA1c		1	0.2940	0.1000	8.6424	0.0033
alcohol	0	1	0.8038	0.4803	2.8008	0.0942
alcohol	1	1	0.6785	0.7956	0.7272	0.3938
alcohol	2	0	0	.	.	.
smoking	0	1	-0.1852	0.4553	0.1655	0.6841
smoking	1	1	-0.3275	0.5583	0.3442	0.5574
smoking	2	0	0	.	.	.
physically_active	0	1	1.1420	0.3726	9.3913	0.0022
physically_active	1	0	0	.	.	.

Odds Ratio Estimates			
Effect	Point Estimate	95% Wald Confidence Limits	
loghsCRP	0.693	0.497	0.966
age	1.054	1.018	1.092
sex 1 vs 2	1.243	0.550	2.806
BMI	1.046	0.959	1.140
hypertension	1.196	0.596	2.401
logLDL_C	0.372	0.157	0.885
HbA1c	1.342	1.103	1.632
alcohol 0 vs 2	2.234	0.871	5.726
alcohol 1 vs 2	1.971	0.414	9.374
smoking 0 vs 2	0.831	0.340	2.028
smoking 1 vs 2	0.721	0.241	2.152
physically_active 0 vs 1	3.133	1.509	6.504

Association of Predicted Probabilities and Observed Responses			
Percent Concordant	75.0	Somers' D	0.502
Percent Discordant	24.8	Gamma	0.503
Percent Tied	0.2	Tau-a	0.249
Pairs	11858	c	0.751

The LOGISTIC Procedure

Model Information	
Data Set	SASUSER.SUN_30DIA1
Response Variable	dspn_y
Number of Response Levels	2
Model	binary logit
Optimization Technique	Fisher's scoring

Number of Observations Read	219
Number of Observations Used	219

Response Profile		
Ordered Value	dspn_y	Total Frequency
1	0	121
2	1	98

Probability modeled is dspn_y=1.

Class Level Information				
Class	Value	Design Variables		
sex	1	1	0	
	2	0	1	
alcohol	0	1	0	0
	1	0	1	0
	2	0	0	1
smoking	0	1	0	0
	1	0	1	0
	2	0	0	1
physically_active	0	1	0	
	1	0	1	

Model Convergence Status
Convergence criterion (GCONV=1E-8) satisfied.

The LOGISTIC Procedure

Model Fit Statistics		
Criterion	Intercept Only	Intercept and Covariates
AIC	303.178	292.523
SC	306.568	336.581
-2 Log L	301.178	266.523

Testing Global Null Hypothesis: BETA=0			
Test	Chi-Square	DF	Pr > ChiSq
Likelihood Ratio	34.6553	12	0.0005
Score	32.2290	12	0.0013
Wald	27.7244	12	0.0061

Type 3 Analysis of Effects			
Effect	DF	Wald Chi-Square	Pr > ChiSq
logl2n	1	0.1110	0.7390
age	1	9.5510	0.0020
sex	1	0.4287	0.5126
BMI	1	0.2775	0.5983
hypertension	1	0.0000	1.0000
logLDL_C	1	5.5572	0.0184
HbA1c	1	6.6352	0.0100
alcohol	2	2.4541	0.2932
smoking	2	0.2311	0.8909
physically_active	1	8.2072	0.0042

Analysis of Maximum Likelihood Estimates						
Parameter		DF	Estimate	Standard Error	Wald Chi-Square	Pr > ChiSq
Intercept		1	-6.7768	2.3957	8.0019	0.0047
logl2n		1	0.0747	0.2242	0.1110	0.7390
age		1	0.0553	0.0179	9.5510	0.0020
sex	1	1	0.2705	0.4131	0.4287	0.5126
sex	2	0	0	.	.	.
BMI		1	0.0224	0.0425	0.2775	0.5983
hypertension		1	0.000015	0.3404	0.0000	1.0000
logLDL_C		1	-1.0460	0.4437	5.5572	0.0184

The LOGISTIC Procedure

Analysis of Maximum Likelihood Estimates						
Parameter		DF	Estimate	Standard Error	Wald Chi-Square	Pr > ChiSq
HbA1c		1	0.2427	0.0942	6.6352	0.0100
alcohol	0	1	0.7445	0.4752	2.4541	0.1172
alcohol	1	1	0.4330	0.7595	0.3250	0.5686
alcohol	2	0	0	.	.	.
smoking	0	1	-0.1484	0.4490	0.1093	0.7409
smoking	1	1	-0.2558	0.5495	0.2168	0.6415
smoking	2	0	0	.	.	.
physically_active	0	1	1.0359	0.3616	8.2072	0.0042
physically_active	1	0	0	.	.	.

Odds Ratio Estimates			
Effect	Point Estimate	95% Wald Confidence Limits	
logl2n	1.078	0.694	1.672
age	1.057	1.020	1.095
sex 1 vs 2	1.311	0.583	2.945
BMI	1.023	0.941	1.111
hypertension	1.000	0.513	1.949
logLDL_C	0.351	0.147	0.838
HbA1c	1.275	1.060	1.533
alcohol 0 vs 2	2.105	0.829	5.344
alcohol 1 vs 2	1.542	0.348	6.831
smoking 0 vs 2	0.862	0.358	2.078
smoking 1 vs 2	0.774	0.264	2.273
physically_active 0 vs 1	2.818	1.387	5.723

Association of Predicted Probabilities and Observed Responses			
Percent Concordant	73.1	Somers' D	0.465
Percent Discordant	26.6	Gamma	0.466
Percent Tied	0.4	Tau-a	0.231
Pairs	11858	c	0.732

The LOGISTIC Procedure

Model Information	
**Data Set**	SASUSER.SUN_30DIA1
**Response Variable**	dspn_y
**Number of Response Levels**	2
**Model**	binary logit
**Optimization Technique**	Fisher's scoring

**Number of Observations Read**	219
**Number of Observations Used**	219

Response Profile		
**Ordered Value**	**dspn_y**	**Total Frequency**
**1**	0	121
**2**	1	98

Probability modeled is dspn_y=1.

Class Level Information				
**Class**	**Value**	**Design Variables**		
**sex**	**1**	1	0	
	**2**	0	1	
**alcohol**	**0**	1	0	0
	**1**	0	1	0
	**2**	0	0	1
**smoking**	**0**	1	0	0
	**1**	0	1	0
	**2**	0	0	1
**physically_active**	**0**	1	0	
	**1**	0	1	

Model Convergence Status
Convergence criterion (GCONV=1E-8) satisfied.

The LOGISTIC Procedure

Model Fit Statistics		
Criterion	Intercept Only	Intercept and Covariates
AIC	303.178	273.303
SC	306.568	327.528
-2 Log L	301.178	241.303

Testing Global Null Hypothesis: BETA=0			
Test	Chi-Square	DF	Pr > ChiSq
Likelihood Ratio	59.8758	15	<.0001
Score	52.2401	15	<.0001
Wald	39.7972	15	0.0005

Type 3 Analysis of Effects			
Effect	DF	Wald Chi-Square	Pr > ChiSq
logadi	1	4.1831	0.0408
age	1	2.7908	0.0948
sex	1	2.4850	0.1149
BMI	1	0.3205	0.5713
hypertension	1	0.6545	0.4185
logLDL_C	1	1.4190	0.2336
HbA1c	1	4.8083	0.0283
alcohol	2	1.9809	0.3714
smoking	2	2.0217	0.3639
physically_active	1	6.3226	0.0119
lipid_lowering	1	2.1825	0.1396
eGFR_EPI	1	0.5940	0.4409
DM_duration	1	11.9962	0.0005

Analysis of Maximum Likelihood Estimates						
Parameter		DF	Estimate	Standard Error	Wald Chi-Square	Pr > ChiSq
Intercept		1	-7.4807	2.4440	9.3688	0.0022
logadi		1	0.5418	0.2649	4.1831	0.0408
age		1	0.0325	0.0195	2.7908	0.0948
sex	1	1	0.7290	0.4624	2.4850	0.1149
sex	2	0	0	.	.	.

The LOGISTIC Procedure

Analysis of Maximum Likelihood Estimates						
Parameter		DF	Estimate	Standard Error	Wald Chi-Square	Pr > ChiSq
BMI		1	0.0265	0.0468	0.3205	0.5713
hypertension		1	-0.3117	0.3853	0.6545	0.4185
logLDL_C		1	-0.5891	0.4945	1.4190	0.2336
HbA1c		1	0.2249	0.1026	4.8083	0.0283
alcohol	0	1	0.6730	0.5146	1.7104	0.1909
alcohol	1	1	0.7709	0.8033	0.9211	0.3372
alcohol	2	0	0	.	.	.
smoking	0	1	0.0969	0.4993	0.0376	0.8462
smoking	1	1	-0.6929	0.5945	1.3585	0.2438
smoking	2	0	0	.	.	.
physically_active	0	1	0.9667	0.3844	6.3226	0.0119
physically_active	1	0	0	.	.	.
lipid_lowering		1	0.5631	0.3812	2.1825	0.1396
eGFR_EPI		1	-0.00548	0.00711	0.5940	0.4409
DM_duration		1	0.0882	0.0255	11.9962	0.0005

Odds Ratio Estimates			
Effect	Point Estimate	95% Wald Confidence Limits	
logadi	1.719	1.023	2.889
age	1.033	0.994	1.073
sex 1 vs 2	2.073	0.837	5.131
BMI	1.027	0.937	1.126
hypertension	0.732	0.344	1.558
logLDL_C	0.555	0.210	1.463
HbA1c	1.252	1.024	1.531
alcohol 0 vs 2	1.960	0.715	5.374
alcohol 1 vs 2	2.162	0.448	10.437
smoking 0 vs 2	1.102	0.414	2.931
smoking 1 vs 2	0.500	0.156	1.604
physically_active 0 vs 1	2.629	1.238	5.585
lipid_lowering	1.756	0.832	3.707
eGFR_EPI	0.995	0.981	1.008
DM_duration	1.092	1.039	1.148

The LOGISTIC Procedure

Association of Predicted Probabilities and Observed Responses			
**Percent Concordant**	78.4	**Somers' D**	0.570
**Percent Discordant**	21.4	**Gamma**	0.572
**Percent Tied**	0.2	**Tau-a**	0.283
**Pairs**	11858	**c**	0.785

The LOGISTIC Procedure

Model Information	
**Data Set**	SASUSER.SUN_30DIA1
**Response Variable**	dspn_y
**Number of Response Levels**	2
**Model**	binary logit
**Optimization Technique**	Fisher's scoring

**Number of Observations Read**	219
**Number of Observations Used**	219

Response Profile		
**Ordered Value**	**dspn_y**	**Total Frequency**
**1**	0	121
**2**	1	98

Probability modeled is dspn_y=1.

Class Level Information				
**Class**	**Value**	**Design Variables**		
**sex**	**1**	1	0	
	**2**	0	1	
**alcohol**	**0**	1	0	0
	**1**	0	1	0
	**2**	0	0	1
**smoking**	**0**	1	0	0
	**1**	0	1	0
	**2**	0	0	1
**physically_active**	**0**	1	0	
	**1**	0	1	

The LOGISTIC Procedure

Model Convergence Status
Convergence criterion (GCONV=1E-8) satisfied.

Model Fit Statistics		
Criterion	Intercept Only	Intercept and Covariates
AIC	303.178	277.864
SC	306.568	332.089
-2 Log L	301.178	245.864

Testing Global Null Hypothesis: BETA=0			
Test	Chi-Square	DF	Pr > ChiSq
Likelihood Ratio	55.3148	15	<.0001
Score	49.4138	15	<.0001
Wald	38.7979	15	0.0007

Type 3 Analysis of Effects			
Effect	DF	Wald Chi-Square	Pr > ChiSq
logIL6	1	0.0458	0.8306
age	1	4.0850	0.0433
sex	1	1.2220	0.2690
BMI	1	0.4997	0.4796
hypertension	1	1.3320	0.2485
logLDL_C	1	1.3103	0.2523
HbA1c	1	4.5573	0.0328
alcohol	2	1.2473	0.5360
smoking	2	1.6563	0.4369
physically_active	1	6.0309	0.0141
lipid_lowering	1	1.8324	0.1758
eGFR_EPI	1	1.5346	0.2154
DM_duration	1	12.9644	0.0003

The LOGISTIC Procedure

Analysis of Maximum Likelihood Estimates						
Parameter		DF	Estimate	Standard Error	Wald Chi-Square	Pr > ChiSq
Intercept		1	-6.1558	2.3039	7.1390	0.0075
logIL6		1	-0.0167	0.0781	0.0458	0.8306
age		1	0.0382	0.0189	4.0850	0.0433
sex	1	1	0.4898	0.4431	1.2220	0.2690
sex	2	0	0	.	.	.
BMI		1	0.0326	0.0461	0.4997	0.4796
hypertension		1	-0.4367	0.3784	1.3320	0.2485
logLDL_C		1	-0.5626	0.4915	1.3103	0.2523
HbA1c		1	0.2177	0.1020	4.5573	0.0328
alcohol	0	1	0.4920	0.5062	0.9445	0.3311
alcohol	1	1	0.6880	0.7978	0.7437	0.3885
alcohol	2	0	0	.	.	.
smoking	0	1	0.1548	0.4912	0.0993	0.7526
smoking	1	1	-0.5650	0.5803	0.9480	0.3302
smoking	2	0	0	.	.	.
physically_active	0	1	0.9371	0.3816	6.0309	0.0141
physically_active	1	0	0	.	.	.
lipid_lowering		1	0.5117	0.3780	1.8324	0.1758
eGFR_EPI		1	-0.00847	0.00684	1.5346	0.2154
DM_duration		1	0.0910	0.0253	12.9644	0.0003

Odds Ratio Estimates			
Effect	Point Estimate	95% Wald Confidence Limits	
logIL6	0.983	0.844	1.146
age	1.039	1.001	1.078
sex 1 vs 2	1.632	0.685	3.889
BMI	1.033	0.944	1.131
hypertension	0.646	0.308	1.357
logLDL_C	0.570	0.217	1.493
HbA1c	1.243	1.018	1.518
alcohol 0 vs 2	1.636	0.606	4.411
alcohol 1 vs 2	1.990	0.417	9.503
smoking 0 vs 2	1.167	0.446	3.057
smoking 1 vs 2	0.568	0.182	1.772

The LOGISTIC Procedure

Odds Ratio Estimates			
Effect	Point Estimate	95% Wald Confidence Limits	
physically_active 0 vs 1	2.553	1.208	5.393
lipid_lowering	1.668	0.795	3.500
eGFR_EPI	0.992	0.978	1.005
DM_duration	1.095	1.042	1.151

Association of Predicted Probabilities and Observed Responses			
Percent Concordant	77.4	Somers' D	0.549
Percent Discordant	22.4	Gamma	0.551
Percent Tied	0.2	Tau-a	0.273
Pairs	11858	c	0.775

The LOGISTIC Procedure

Model Information	
Data Set	SASUSER.SUN_30DIA1
Response Variable	dspn_y
Number of Response Levels	2
Model	binary logit
Optimization Technique	Fisher's scoring

Number of Observations Read	219
Number of Observations Used	219

Response Profile		
Ordered Value	dspn_y	Total Frequency
1	0	121
2	1	98

Probability modeled is dspn_y=1.

Class Level Information				
Class	Value	Design Variables		
sex	1	1	0	
	2	0	1	
alcohol	0	1	0	0
	1	0	1	0

The LOGISTIC Procedure

Class Level Information				
Class	Value	Design Variables		
	2	0	0	1
smoking	0	1	0	0
	1	0	1	0
	2	0	0	1
physically_active	0	1	0	
	1	0	1	

Model Convergence Status
Convergence criterion (GCONV=1E-8) satisfied.

Model Fit Statistics		
Criterion	Intercept Only	Intercept and Covariates
AIC	303.178	273.865
SC	306.568	328.090
-2 Log L	301.178	241.865

Testing Global Null Hypothesis: BETA=0			
Test	Chi-Square	DF	Pr > ChiSq
Likelihood Ratio	59.3133	15	<.0001
Score	51.9254	15	<.0001
Wald	40.2126	15	0.0004

Type 3 Analysis of Effects			
Effect	DF	Wald Chi-Square	Pr > ChiSq
logIL1beta	1	3.5811	0.0584
age	1	3.6577	0.0558
sex	1	1.0938	0.2956
BMI	1	0.5313	0.4660
hypertension	1	1.1282	0.2882
logLDL_C	1	1.0101	0.3149
HbA1c	1	3.6533	0.0560
alcohol	2	1.7904	0.4085
smoking	2	1.6578	0.4365

The LOGISTIC Procedure

Type 3 Analysis of Effects			
Effect	DF	Wald Chi-Square	Pr > ChiSq
physically_active	1	6.6766	0.0098
lipid_lowering	1	1.8773	0.1706
eGFR_EPI	1	1.0388	0.3081
DM_duration	1	12.9635	0.0003

Analysis of Maximum Likelihood Estimates						
Parameter		DF	Estimate	Standard Error	Wald Chi-Square	Pr > ChiSq
Intercept		1	-6.0726	2.3257	6.8179	0.0090
logIL1beta		1	-0.2493	0.1318	3.5811	0.0584
age		1	0.0367	0.0192	3.6577	0.0558
sex	1	1	0.4701	0.4495	1.0938	0.2956
sex	2	0	0	.	.	.
BMI		1	0.0339	0.0465	0.5313	0.4660
hypertension		1	-0.4033	0.3797	1.1282	0.2882
logLDL_C		1	-0.4956	0.4931	1.0101	0.3149
HbA1c		1	0.1979	0.1035	3.6533	0.0560
alcohol	0	1	0.5074	0.5070	1.0017	0.3169
alcohol	1	1	0.9996	0.8482	1.3890	0.2386
alcohol	2	0	0	.	.	.
smoking	0	1	0.1049	0.4939	0.0451	0.8318
smoking	1	1	-0.6117	0.5878	1.0830	0.2980
smoking	2	0	0	.	.	.
physically_active	0	1	0.9929	0.3842	6.6766	0.0098
physically_active	1	0	0	.	.	.
lipid_lowering		1	0.5211	0.3803	1.8773	0.1706
eGFR_EPI		1	-0.00707	0.00693	1.0388	0.3081
DM_duration		1	0.0918	0.0255	12.9635	0.0003

Odds Ratio Estimates			
Effect	Point Estimate	95% Wald Confidence Limits	
logIL1beta	0.779	0.602	1.009
age	1.037	0.999	1.077
sex 1 vs 2	1.600	0.663	3.861
BMI	1.034	0.944	1.133

The LOGISTIC Procedure

Odds Ratio Estimates			
Effect	Point Estimate	95% Wald Confidence Limits	
hypertension	0.668	0.317	1.406
logLDL_C	0.609	0.232	1.601
HbA1c	1.219	0.995	1.493
alcohol 0 vs 2	1.661	0.615	4.487
alcohol 1 vs 2	2.717	0.515	14.325
smoking 0 vs 2	1.111	0.422	2.924
smoking 1 vs 2	0.542	0.171	1.717
physically_active 0 vs 1	2.699	1.271	5.731
lipid_lowering	1.684	0.799	3.548
eGFR_EPI	0.993	0.980	1.007
DM_duration	1.096	1.043	1.152

Association of Predicted Probabilities and Observed Responses			
Percent Concordant	78.7	Somers' D	0.576
Percent Discordant	21.1	Gamma	0.577
Percent Tied	0.2	Tau-a	0.286
Pairs	11858	c	0.788

The LOGISTIC Procedure

Model Information	
Data Set	SASUSER.SUN_30DIA1
Response Variable	dspn_y
Number of Response Levels	2
Model	binary logit
Optimization Technique	Fisher's scoring

Number of Observations Read	219
Number of Observations Used	219

Response Profile		
Ordered Value	dspn_y	Total Frequency
1	0	121
2	1	98

Probability modeled is dspn_y=1.

The LOGISTIC Procedure

Class Level Information				
Class	Value	Design Variables		
sex	1	1	0	
	2	0	1	
alcohol	0	1	0	0
	1	0	1	0
	2	0	0	1
smoking	0	1	0	0
	1	0	1	0
	2	0	0	1
physically_active	0	1	0	
	1	0	1	

Model Convergence Status
Convergence criterion (GCONV=1E-8) satisfied.

Model Fit Statistics		
Criterion	Intercept Only	Intercept and Covariates
AIC	303.178	276.348
SC	306.568	330.574
-2 Log L	301.178	244.348

Testing Global Null Hypothesis: BETA=0			
Test	Chi-Square	DF	Pr > ChiSq
Likelihood Ratio	56.8301	15	<.0001
Score	50.8075	15	<.0001
Wald	39.8044	15	0.0005

Type 3 Analysis of Effects			
Effect	DF	Wald Chi-Square	Pr > ChiSq
Leptin	1	1.5051	0.2199
age	1	3.4567	0.0630
sex	1	2.3566	0.1248
BMI	1	0.0012	0.9723
hypertension	1	1.3632	0.2430

The LOGISTIC Procedure

Type 3 Analysis of Effects			
**Effect**	**DF**	**Wald Chi-Square**	**Pr > ChiSq**
**logLDL_C**	1	0.7884	0.3746
**HbA1c**	1	4.9273	0.0264
**alcohol**	2	0.9818	0.6121
**smoking**	2	2.0433	0.3600
**physically_active**	1	5.5288	0.0187
**lipid_lowering**	1	2.1551	0.1421
**eGFR_EPI**	1	1.0378	0.3083
**DM_duration**	1	11.4383	0.0007

Analysis of Maximum Likelihood Estimates						
**Parameter**		**DF**	**Estimate**	**Standard Error**	**Wald Chi-Square**	**Pr > ChiSq**
**Intercept**		1	-5.8439	2.3177	6.3576	0.0117
**Leptin**		1	0.0213	0.0174	1.5051	0.2199
**age**		1	0.0354	0.0191	3.4567	0.0630
**sex**	**1**	1	0.7613	0.4959	2.3566	0.1248
**sex**	**2**	0	0	.	.	.
**BMI**		1	0.00183	0.0527	0.0012	0.9723
**hypertension**		1	-0.4412	0.3778	1.3632	0.2430
**logLDL_C**		1	-0.4458	0.5020	0.7884	0.3746
**HbA1c**		1	0.2258	0.1017	4.9273	0.0264
**alcohol**	**0**	1	0.4422	0.5061	0.7631	0.3824
**alcohol**	**1**	1	0.6010	0.7927	0.5749	0.4483
**alcohol**	**2**	0	0	.	.	.
**smoking**	**0**	1	0.2526	0.5018	0.2534	0.6147
**smoking**	**1**	1	-0.5638	0.5819	0.9387	0.3326
**smoking**	**2**	0	0	.	.	.
**physically_active**	**0**	1	0.8984	0.3821	5.5288	0.0187
**physically_active**	**1**	0	0	.	.	.
**lipid_lowering**		1	0.5607	0.3819	2.1551	0.1421
**eGFR_EPI**		1	-0.00704	0.00691	1.0378	0.3083
**DM_duration**		1	0.0865	0.0256	11.4383	0.0007

The LOGISTIC Procedure

Odds Ratio Estimates			
Effect	Point Estimate	95% Wald Confidence Limits	
Leptin	1.022	0.987	1.057
age	1.036	0.998	1.076
sex 1 vs 2	2.141	0.810	5.659
BMI	1.002	0.904	1.111
hypertension	0.643	0.307	1.349
logLDL_C	0.640	0.239	1.713
HbA1c	1.253	1.027	1.530
alcohol 0 vs 2	1.556	0.577	4.196
alcohol 1 vs 2	1.824	0.386	8.624
smoking 0 vs 2	1.287	0.482	3.442
smoking 1 vs 2	0.569	0.182	1.780
physically_active 0 vs 1	2.456	1.161	5.193
lipid_lowering	1.752	0.829	3.703
eGFR_EPI	0.993	0.980	1.007
DM_duration	1.090	1.037	1.146

Association of Predicted Probabilities and Observed Responses			
Percent Concordant	77.7	Somers' D	0.556
Percent Discordant	22.1	Gamma	0.557
Percent Tied	0.2	Tau-a	0.276
Pairs	11858	c	0.778

The LOGISTIC Procedure

Model Information	
Data Set	SASUSER.SUN_30DIA1
Response Variable	dspn_y
Number of Response Levels	2
Model	binary logit
Optimization Technique	Fisher's scoring

Number of Observations Read	219
Number of Observations Used	219

The LOGISTIC Procedure

Response Profile		
Ordered Value	dspn_y	Total Frequency
1	0	121
2	1	98

Probability modeled is dspn_y=1.

Class Level Information				
Class	Value	Design Variables		
sex	1	1	0	
	2	0	1	
alcohol	0	1	0	0
	1	0	1	0
	2	0	0	1
smoking	0	1	0	0
	1	0	1	0
	2	0	0	1
physically_active	0	1	0	
	1	0	1	

Model Convergence Status
Convergence criterion (GCONV=1E-8) satisfied.

Model Fit Statistics		
Criterion	Intercept Only	Intercept and Covariates
AIC	303.178	276.309
SC	306.568	330.534
-2 Log L	301.178	244.309

Testing Global Null Hypothesis: BETA=0			
Test	Chi-Square	DF	Pr > ChiSq
Likelihood Ratio	56.8693	15	<.0001
Score	50.5106	15	<.0001
Wald	39.5320	15	0.0005

The LOGISTIC Procedure

Type 3 Analysis of Effects			
Effect	DF	Wald Chi-Square	Pr > ChiSq
logtnfa	1	1.5586	0.2119
age	1	3.8132	0.0509
sex	1	1.3518	0.2450
BMI	1	0.5696	0.4504
hypertension	1	1.2816	0.2576
logLDL_C	1	1.3270	0.2493
HbA1c	1	4.5649	0.0326
alcohol	2	1.3304	0.5142
smoking	2	1.7530	0.4162
physically_active	1	6.2115	0.0127
lipid_lowering	1	2.0807	0.1492
eGFR_EPI	1	1.4052	0.2358
DM_duration	1	13.5517	0.0002

Analysis of Maximum Likelihood Estimates						
Parameter		DF	Estimate	Standard Error	Wald Chi-Square	Pr > ChiSq
Intercept		1	-5.8149	2.3178	6.2938	0.0121
logtnfa		1	-0.1900	0.1522	1.5586	0.2119
age		1	0.0371	0.0190	3.8132	0.0509
sex	1	1	0.5192	0.4466	1.3518	0.2450
sex	2	0	0	.	.	.
BMI		1	0.0348	0.0461	0.5696	0.4504
hypertension		1	-0.4278	0.3779	1.2816	0.2576
logLDL_C		1	-0.5633	0.4890	1.3270	0.2493
HbA1c		1	0.2188	0.1024	4.5649	0.0326
alcohol	0	1	0.5048	0.5054	0.9974	0.3179
alcohol	1	1	0.7318	0.8152	0.8060	0.3693
alcohol	2	0	0	.	.	.
smoking	0	1	0.1266	0.4932	0.0659	0.7974
smoking	1	1	-0.6084	0.5838	1.0861	0.2973
smoking	2	0	0	.	.	.
physically_active	0	1	0.9526	0.3822	6.2115	0.0127
physically_active	1	0	0	.	.	.
lipid_lowering		1	0.5486	0.3803	2.0807	0.1492

The LOGISTIC Procedure

Analysis of Maximum Likelihood Estimates						
Parameter		DF	Estimate	Standard Error	Wald Chi-Square	Pr > ChiSq
eGFR_EPI		1	-0.00813	0.00686	1.4052	0.2358
DM_duration		1	0.0937	0.0254	13.5517	0.0002

Odds Ratio Estimates			
Effect	Point Estimate	95% Wald Confidence Limits	
logtnfa	0.827	0.614	1.114
age	1.038	1.000	1.077
sex 1 vs 2	1.681	0.700	4.033
BMI	1.035	0.946	1.133
hypertension	0.652	0.311	1.367
logLDL_C	0.569	0.218	1.485
HbA1c	1.245	1.018	1.521
alcohol 0 vs 2	1.657	0.615	4.461
alcohol 1 vs 2	2.079	0.421	10.273
smoking 0 vs 2	1.135	0.432	2.984
smoking 1 vs 2	0.544	0.173	1.709
physically_active 0 vs 1	2.592	1.226	5.483
lipid_lowering	1.731	0.821	3.647
eGFR_EPI	0.992	0.979	1.005
DM_duration	1.098	1.045	1.154

Association of Predicted Probabilities and Observed Responses			
Percent Concordant	77.9	Somers' D	0.560
Percent Discordant	21.9	Gamma	0.561
Percent Tied	0.2	Tau-a	0.278
Pairs	11858	c	0.780

The LOGISTIC Procedure

Model Information	
Data Set	SASUSER.SUN_30DIA1
Response Variable	dspn_y
Number of Response Levels	2
Model	binary logit
Optimization Technique	Fisher's scoring

The LOGISTIC Procedure

**Number of Observations Read**	219
**Number of Observations Used**	219

Response Profile		
**Ordered Value**	**dspn_y**	**Total Frequency**
**1**	0	121
**2**	1	98

Probability modeled is dspn_y=1.

Class Level Information				
**Class**	**Value**	**Design Variables**		
**sex**	**1**	1	0	
	**2**	0	1	
**alcohol**	**0**	1	0	0
	**1**	0	1	0
	**2**	0	0	1
**smoking**	**0**	1	0	0
	**1**	0	1	0
	**2**	0	0	1
**physically_active**	**0**	1	0	
	**1**	0	1	

Model Convergence Status
Convergence criterion (GCONV=1E-8) satisfied.

Model Fit Statistics		
**Criterion**	**Intercept Only**	**Intercept and Covariates**
**AIC**	303.178	271.465
**SC**	306.568	325.690
**-2 Log L**	301.178	239.465

The LOGISTIC Procedure

Testing Global Null Hypothesis: BETA=0			
**Test**	**Chi-Square**	**DF**	**Pr > ChiSq**
**Likelihood Ratio**	61.7139	15	<.0001
**Score**	54.8105	15	<.0001
**Wald**	42.4657	15	0.0002

Type 3 Analysis of Effects			
**Effect**	**DF**	**Wald Chi-Square**	**Pr > ChiSq**
**logFbg**	1	6.1324	0.0133
**age**	1	4.2260	0.0398
**sex**	1	1.9842	0.1590
**BMI**	1	0.1005	0.7513
**hypertension**	1	1.4664	0.2259
**logLDL_C**	1	2.3165	0.1280
**HbA1c**	1	3.2901	0.0697
**alcohol**	2	0.5769	0.7494
**smoking**	2	2.5357	0.2814
**physically_active**	1	6.9294	0.0085
**lipid_lowering**	1	1.5366	0.2151
**eGFR_EPI**	1	0.2226	0.6371
**DM_duration**	1	13.7192	0.0002

Analysis of Maximum Likelihood Estimates						
**Parameter**		**DF**	**Estimate**	**Standard Error**	**Wald Chi-Square**	**Pr > ChiSq**
**Intercept**		1	-8.2736	2.5055	10.9045	0.0010
**logFbg**		1	2.0749	0.8379	6.1324	0.0133
**age**		1	0.0400	0.0194	4.2260	0.0398
**sex**	**1**	1	0.6460	0.4586	1.9842	0.1590
**sex**	**2**	0	0	.	.	.
**BMI**		1	0.0150	0.0474	0.1005	0.7513
**hypertension**		1	-0.4669	0.3856	1.4664	0.2259
**logLDL_C**		1	-0.7649	0.5025	2.3165	0.1280
**HbA1c**		1	0.1897	0.1046	3.2901	0.0697
**alcohol**	**0**	1	0.3794	0.5159	0.5409	0.4621
**alcohol**	**1**	1	0.3691	0.8087	0.2083	0.6481
**alcohol**	**2**	0	0	.	.	.

The LOGISTIC Procedure

Analysis of Maximum Likelihood Estimates						
Parameter		DF	Estimate	Standard Error	Wald Chi-Square	Pr > ChiSq
smoking	0	1	0.2683	0.5015	0.2862	0.5927
smoking	1	1	-0.6642	0.5902	1.2665	0.2604
smoking	2	0	0	.	.	.
physically_active	0	1	1.0263	0.3899	6.9294	0.0085
physically_active	1	0	0	.	.	.
lipid_lowering		1	0.4753	0.3834	1.5366	0.2151
eGFR_EPI		1	-0.00340	0.00720	0.2226	0.6371
DM_duration		1	0.0947	0.0256	13.7192	0.0002

Odds Ratio Estimates			
Effect	Point Estimate	95% Wald Confidence Limits	
logFbg	7.964	1.541	41.144
age	1.041	1.002	1.081
sex 1 vs 2	1.908	0.777	4.688
BMI	1.015	0.925	1.114
hypertension	0.627	0.294	1.335
logLDL_C	0.465	0.174	1.246
HbA1c	1.209	0.985	1.484
alcohol 0 vs 2	1.461	0.532	4.018
alcohol 1 vs 2	1.446	0.296	7.058
smoking 0 vs 2	1.308	0.489	3.494
smoking 1 vs 2	0.515	0.162	1.637
physically_active 0 vs 1	2.791	1.300	5.993
lipid_lowering	1.608	0.759	3.410
eGFR_EPI	0.997	0.983	1.011
DM_duration	1.099	1.046	1.156

Association of Predicted Probabilities and Observed Responses			
Percent Concordant	79.2	Somers' D	0.584
Percent Discordant	20.7	Gamma	0.585
Percent Tied	0.1	Tau-a	0.290
Pairs	11858	c	0.792

The LOGISTIC Procedure

Model Information	
Data Set	SASUSER.SUN_30DIA1
Response Variable	dspn_y
Number of Response Levels	2
Model	binary logit
Optimization Technique	Fisher's scoring

Number of Observations Read	219
Number of Observations Used	219

Response Profile		
Ordered Value	dspn_y	Total Frequency
1	0	121
2	1	98

Probability modeled is dspn_y=1.

Class Level Information				
Class	Value	Design Variables		
sex	1	1	0	
	2	0	1	
alcohol	0	1	0	0
	1	0	1	0
	2	0	0	1
smoking	0	1	0	0
	1	0	1	0
	2	0	0	1
physically_active	0	1	0	
	1	0	1	

Model Convergence Status
Convergence criterion (GCONV=1E-8) satisfied.

The LOGISTIC Procedure

Model Fit Statistics		
Criterion	Intercept Only	Intercept and Covariates
AIC	303.178	270.832
SC	306.568	325.058
-2 Log L	301.178	238.832

Testing Global Null Hypothesis: BETA=0			
Test	Chi-Square	DF	Pr > ChiSq
Likelihood Ratio	62.3460	15	<.0001
Score	54.6109	15	<.0001
Wald	41.8520	15	0.0002

Type 3 Analysis of Effects			
Effect	DF	Wald Chi-Square	Pr > ChiSq
loghsCRP	1	6.7381	0.0094
age	1	3.2355	0.0721
sex	1	0.9008	0.3426
BMI	1	1.4788	0.2240
hypertension	1	0.3536	0.5521
logLDL_C	1	0.8467	0.3575
HbA1c	1	7.4916	0.0062
alcohol	2	2.2427	0.3258
smoking	2	2.2002	0.3328
physically_active	1	7.1837	0.0074
lipid_lowering	1	1.2382	0.2658
eGFR_EPI	1	3.5524	0.0595
DM_duration	1	12.6295	0.0004

Analysis of Maximum Likelihood Estimates						
Parameter		DF	Estimate	Standard Error	Wald Chi-Square	Pr > ChiSq
Intercept		1	-7.1124	2.3337	9.2887	0.0023
loghsCRP		1	-0.4869	0.1876	6.7381	0.0094
age		1	0.0344	0.0191	3.2355	0.0721
sex	1	1	0.4250	0.4478	0.9008	0.3426
sex	2	0	0	.	.	.

The LOGISTIC Procedure

Analysis of Maximum Likelihood Estimates						
Parameter		DF	Estimate	Standard Error	Wald Chi-Square	Pr > ChiSq
BMI		1	0.0581	0.0478	1.4788	0.2240
hypertension		1	-0.2315	0.3892	0.3536	0.5521
logLDL_C		1	-0.4550	0.4945	0.8467	0.3575
HbA1c		1	0.3024	0.1105	7.4916	0.0062
alcohol	0	1	0.6059	0.5189	1.3637	0.2429
alcohol	1	1	1.1019	0.8487	1.6857	0.1942
alcohol	2	0	0	.	.	.
smoking	0	1	0.0865	0.4980	0.0302	0.8621
smoking	1	1	-0.7274	0.5973	1.4833	0.2233
smoking	2	0	0	.	.	.
physically_active	0	1	1.0628	0.3965	7.1837	0.0074
physically_active	1	0	0	.	.	.
lipid_lowering		1	0.4277	0.3843	1.2382	0.2658
eGFR_EPI		1	-0.0139	0.00736	3.5524	0.0595
DM_duration		1	0.0916	0.0258	12.6295	0.0004

Odds Ratio Estimates			
Effect	Point Estimate	95% Wald Confidence Limits	
loghsCRP	0.615	0.425	0.888
age	1.035	0.997	1.075
sex 1 vs 2	1.530	0.636	3.679
BMI	1.060	0.965	1.164
hypertension	0.793	0.370	1.701
logLDL_C	0.634	0.241	1.672
HbA1c	1.353	1.090	1.680
alcohol 0 vs 2	1.833	0.663	5.068
alcohol 1 vs 2	3.010	0.570	15.886
smoking 0 vs 2	1.090	0.411	2.894
smoking 1 vs 2	0.483	0.150	1.558
physically_active 0 vs 1	2.894	1.331	6.297
lipid_lowering	1.534	0.722	3.257
eGFR_EPI	0.986	0.972	1.001
DM_duration	1.096	1.042	1.153

The LOGISTIC Procedure

Association of Predicted Probabilities and Observed Responses			
**Percent Concordant**	79.7	**Somers' D**	0.596
**Percent Discordant**	20.1	**Gamma**	0.597
**Percent Tied**	0.2	**Tau-a**	0.296
**Pairs**	11858	**c**	0.798

The LOGISTIC Procedure

Model Information	
**Data Set**	SASUSER.SUN_30DIA1
**Response Variable**	dspn_y
**Number of Response Levels**	2
**Model**	binary logit
**Optimization Technique**	Fisher's scoring

**Number of Observations Read**	219
**Number of Observations Used**	219

Response Profile		
**Ordered Value**	**dspn_y**	**Total Frequency**
**1**	0	121
**2**	1	98

Probability modeled is dspn_y=1.

Class Level Information				
**Class**	**Value**	**Design Variables**		
**sex**	**1**	1	0	
	**2**	0	1	
**alcohol**	**0**	1	0	0
	**1**	0	1	0
	**2**	0	0	1
**smoking**	**0**	1	0	0
	**1**	0	1	0
	**2**	0	0	1
**physically_active**	**0**	1	0	
	**1**	0	1	

The LOGISTIC Procedure

Model Convergence Status
Convergence criterion (GCONV=1E-8) satisfied.

Model Fit Statistics		
Criterion	Intercept Only	Intercept and Covariates
AIC	303.178	277.728
SC	306.568	331.953
-2 Log L	301.178	245.728

Testing Global Null Hypothesis: BETA=0			
Test	Chi-Square	DF	Pr > ChiSq
Likelihood Ratio	55.4510	15	<.0001
Score	49.5362	15	<.0001
Wald	38.9511	15	0.0007

Type 3 Analysis of Effects			
Effect	DF	Wald Chi-Square	Pr > ChiSq
logl2n	1	0.1813	0.6703
age	1	3.8228	0.0506
sex	1	1.2509	0.2634
BMI	1	0.4961	0.4812
hypertension	1	1.4695	0.2254
logLDL_C	1	1.4191	0.2336
HbA1c	1	4.5560	0.0328
alcohol	2	1.2039	0.5477
smoking	2	1.6747	0.4329
physically_active	1	5.9029	0.0151
lipid_lowering	1	1.8823	0.1701
eGFR_EPI	1	1.6703	0.1962
DM_duration	1	13.2235	0.0003

The LOGISTIC Procedure

Analysis of Maximum Likelihood Estimates						
Parameter		DF	Estimate	Standard Error	Wald Chi-Square	Pr > ChiSq
Intercept		1	-5.5772	2.7244	4.1909	0.0406
logl2n		1	-0.1027	0.2412	0.1813	0.6703
age		1	0.0372	0.0190	3.8228	0.0506
sex	1	1	0.4952	0.4427	1.2509	0.2634
sex	2	0	0	.	.	.
BMI		1	0.0325	0.0462	0.4961	0.4812
hypertension		1	-0.4580	0.3778	1.4695	0.2254
logLDL_C		1	-0.5845	0.4907	1.4191	0.2336
HbA1c		1	0.2177	0.1020	4.5560	0.0328
alcohol	0	1	0.4825	0.5065	0.9076	0.3408
alcohol	1	1	0.6806	0.7962	0.7308	0.3926
alcohol	2	0	0	.	.	.
smoking	0	1	0.1546	0.4915	0.0989	0.7531
smoking	1	1	-0.5663	0.5795	0.9551	0.3284
smoking	2	0	0	.	.	.
physically_active	0	1	0.9268	0.3815	5.9029	0.0151
physically_active	1	0	0	.	.	.
lipid_lowering		1	0.5192	0.3784	1.8823	0.1701
eGFR_EPI		1	-0.00895	0.00693	1.6703	0.1962
DM_duration		1	0.0921	0.0253	13.2235	0.0003

Odds Ratio Estimates			
Effect	Point Estimate	95% Wald Confidence Limits	
logl2n	0.902	0.562	1.448
age	1.038	1.000	1.077
sex 1 vs 2	1.641	0.689	3.908
BMI	1.033	0.944	1.131
hypertension	0.633	0.302	1.326
logLDL_C	0.557	0.213	1.458
HbA1c	1.243	1.018	1.518
alcohol 0 vs 2	1.620	0.600	4.372
alcohol 1 vs 2	1.975	0.415	9.404
smoking 0 vs 2	1.167	0.445	3.058
smoking 1 vs 2	0.568	0.182	1.767

The LOGISTIC Procedure

Odds Ratio Estimates

Effect	Point Estimate	95% Wald Confidence Limits	
physically_active 0 vs 1	2.526	1.196	5.336
lipid_lowering	1.681	0.801	3.529
eGFR_EPI	0.991	0.978	1.005
DM_duration	1.096	1.043	1.152

Association of Predicted Probabilities and Observed Responses

Percent Concordant	77.4	Somers' D	0.549
Percent Discordant	22.5	Gamma	0.550
Percent Tied	0.2	Tau-a	0.273
Pairs	11858	c	0.775
