# Supplementary material for: Serum Adiponectin Levels Are Positively Associated With Diabetic Peripheral Neuropathy in Chinese Patients With Type 2 Diabetes
Source: Front Endocrinol (Lausanne). 2020 Nov 25;11:567959. doi: 10.3389/fendo.2020.567959 (PMC7724032; doi:10.3389/fendo.2020.567959)
Supplement: Supplementary file 2 [file DataSheet_2.pdf]

The LOGISTIC Procedure

Model Information

**Data Set**	SASUSER.SUN_30DIA1
**Response Variable**	dspn_y
**Number of Response Levels**	2
**Model**	binary logit
**Optimization Technique**	Fisher's scoring

**Number of Observations Read**	219
**Number of Observations Used**	219

Response Profile

Ordered Value	dspn_y	Total Frequency
**1**	0	121
**2**	1	98

Probability modeled is dspn_y=1.

Class Level Information

Class	Value	Design Variables		
**sex**	**1**	1	0	
	**2**	0	1	
**alcohol**	**0**	1	0	0
	**1**	0	1	0
	**2**	0	0	1
**smoking**	**0**	1	0	0
	**1**	0	1	0
	**2**	0	0	1
**physically_active**	**0**	1	0	
	**1**	0	1	

Model Convergence Status

Convergence criterion (GCONV=1E-8) satisfied.

Model Fit Statistics

Criterion	Intercept Only	Intercept and Covariates
**AIC**	303.178	275.024

Model Fit Statistics

Criterion	Intercept Only	Intercept and Covariates
**SC**	306.568	332.638
**-2 Log L**	301.178	241.024

Testing Global Null Hypothesis: BETA=0

Test	Chi-Square	DF	Pr > ChiSq
**Likelihood Ratio**	60.1547	16	<.0001
**Score**	52.7155	16	<.0001
**Wald**	40.1759	16	0.0007

Type 3 Analysis of Effects

Effect	DF	Wald Chi-Square	Pr > ChiSq
**logadi**	1	4.5148	0.0336
**logadi*sex**	1	0.2794	0.5971
**age**	1	2.8242	0.0929
**sex**	1	0.0167	0.8970
**BMI**	1	0.3502	0.5540
**hypertension**	1	0.7499	0.3865
**logLDL_C**	1	1.3505	0.2452
**HbA1c**	1	4.5986	0.0320
**alcohol**	2	2.1745	0.3371
**smoking**	2	2.1846	0.3354
**physically_active**	1	6.3267	0.0119
**eGFR_EPI**	1	0.6203	0.4310
**lipid_lowering**	1	2.2788	0.1312
**DM_duration**	1	12.1555	0.0005

Analysis of Maximum Likelihood Estimates

Parameter		DF	Estimate	Standard Error	Wald Chi-Square	Pr > ChiSq
**Intercept**		1	-7.2422	2.4697	8.5991	0.0034
**logadi**		1	0.4285	0.3323	1.6632	0.1972
**logadi*sex**	**1**	1	0.2719	0.5144	0.2794	0.5971
**logadi*sex**	**2**	0	0	.	.	.
**age**		1	0.0326	0.0194	2.8242	0.0929
**sex**	**1**	1	0.1530	1.1824	0.0167	0.8970

Analysis of Maximum Likelihood Estimates

Parameter		DF	Estimate	Standard Error	Wald Chi-Square	Pr > ChiSq
sex	2	0	0	.	.	.
BMI		1	0.0277	0.0468	0.3502	0.5540
hypertension		1	-0.3361	0.3882	0.7499	0.3865
logLDL_C		1	-0.5756	0.4953	1.3505	0.2452
HbA1c		1	0.2210	0.1031	4.5986	0.0320
alcohol	0	1	0.7235	0.5264	1.8888	0.1693
alcohol	1	1	0.8303	0.8178	1.0309	0.3100
alcohol	2	0	0	.	.	.
smoking	0	1	0.0652	0.5040	0.0168	0.8970
smoking	1	1	-0.7579	0.6109	1.5392	0.2147
smoking	2	0	0	.	.	.
physically_active	0	1	0.9678	0.3848	6.3267	0.0119
physically_active	1	0	0	.	.	.
eGFR_EPI		1	-0.00560	0.00711	0.6203	0.4310
lipid_lowering		1	0.5790	0.3836	2.2788	0.1312
DM_duration		1	0.0892	0.0256	12.1555	0.0005

Odds Ratio Estimates

Effect	Point Estimate	95% Wald Confidence Limits	
age	1.033	0.995	1.073
BMI	1.028	0.938	1.127
hypertension	0.715	0.334	1.529
logLDL_C	0.562	0.213	1.485
HbA1c	1.247	1.019	1.527
alcohol 0 vs 2	2.062	0.735	5.785
alcohol 1 vs 2	2.294	0.462	11.396
smoking 0 vs 2	1.067	0.397	2.867
smoking 1 vs 2	0.469	0.142	1.552
physically_active 0 vs 1	2.632	1.238	5.596
eGFR_EPI	0.994	0.981	1.008
lipid_lowering	1.784	0.841	3.784
DM_duration	1.093	1.040	1.149

Association of Predicted Probabilities and Observed Responses

**Percent Concordant**	78.5	**Somers' D**	0.571
**Percent Discordant**	21.3	**Gamma**	0.573
**Percent Tied**	0.2	**Tau-a**	0.284
**Pairs**	11858	**c**	0.786

model3 for logIL6

The LOGISTIC Procedure

Model Information

**Data Set**	SASUSER.SUN_30DIA1
**Response Variable**	dspn_y
**Number of Response Levels**	2
**Model**	binary logit
**Optimization Technique**	Fisher's scoring

**Number of Observations Read**	219
**Number of Observations Used**	219

Response Profile

Ordered Value	dspn_y	Total Frequency
**1**	0	121
**2**	1	98

Probability modeled is dspn_y=1.

Class Level Information

Class	Value	Design Variables		
**sex**	**1**	1	0	
	**2**	0	1	
**alcohol**	**0**	1	0	0
	**1**	0	1	0
	**2**	0	0	1
**smoking**	**0**	1	0	0
	**1**	0	1	0
	**2**	0	0	1
**physically_active**	**0**	1	0	
	**1**	0	1	

Model Convergence Status

Convergence criterion (GCONV=1E-8) satisfied.

Model Fit Statistics

Criterion	Intercept Only	Intercept and Covariates
AIC	303.178	277.194
SC	306.568	334.808
-2 Log L	301.178	243.194

Testing Global Null Hypothesis: BETA=0

Test	Chi-Square	DF	Pr > ChiSq
Likelihood Ratio	57.9847	16	<.0001
Score	51.5604	16	<.0001
Wald	40.4514	16	0.0007

Type 3 Analysis of Effects

Effect	DF	Wald Chi-Square	Pr > ChiSq
logIL6	1	0.0646	0.7994
logIL6*sex	1	2.5806	0.1082
age	1	5.0169	0.0251
sex	1	0.0366	0.8482
BMI	1	0.4590	0.4981
hypertension	1	0.8725	0.3503
logLDL_C	1	1.3096	0.2525
HbA1c	1	4.2234	0.0399
alcohol	2	1.4051	0.4953
smoking	2	1.8286	0.4008
physically_active	1	5.9104	0.0151
eGFR_EPI	1	1.3445	0.2462
lipid_lowering	1	1.9848	0.1589
DM_duration	1	13.1426	0.0003

Analysis of Maximum Likelihood Estimates

Parameter		DF	Estimate	Standard Error	Wald Chi-Square	Pr > ChiSq
Intercept		1	-6.2665	2.3320	7.2209	0.0072
logIL6		1	-0.1537	0.1186	1.6799	0.1949
logIL6*sex	1	1	0.2667	0.1660	2.5806	0.1082
logIL6*sex	2	0	0	.	.	.
age		1	0.0438	0.0196	5.0169	0.0251

Analysis of Maximum Likelihood Estimates

Parameter		DF	Estimate	Standard Error	Wald Chi-Square	Pr > ChiSq
sex	1	1	-0.1109	0.5793	0.0366	0.8482
sex	2	0	0	.	.	.
BMI		1	0.0314	0.0464	0.4590	0.4981
hypertension		1	-0.3568	0.3820	0.8725	0.3503
logLDL_C		1	-0.5640	0.4929	1.3096	0.2525
HbA1c		1	0.2121	0.1032	4.2234	0.0399
alcohol	0	1	0.5815	0.5130	1.2852	0.2569
alcohol	1	1	0.5707	0.7958	0.5143	0.4733
alcohol	2	0	0	.	.	.
smoking	0	1	0.1169	0.4910	0.0567	0.8118
smoking	1	1	-0.6342	0.5843	1.1783	0.2777
smoking	2	0	0	.	.	.
physically_active	0	1	0.9332	0.3838	5.9104	0.0151
physically_active	1	0	0	.	.	.
eGFR_EPI		1	-0.00802	0.00692	1.3445	0.2462
lipid_lowering		1	0.5373	0.3814	1.9848	0.1589
DM_duration		1	0.0920	0.0254	13.1426	0.0003

Odds Ratio Estimates

Effect	Point Estimate	95% Wald Confidence Limits	
age	1.045	1.005	1.086
BMI	1.032	0.942	1.130
hypertension	0.700	0.331	1.480
logLDL_C	0.569	0.217	1.495
HbA1c	1.236	1.010	1.513
alcohol 0 vs 2	1.789	0.655	4.888
alcohol 1 vs 2	1.770	0.372	8.420
smoking 0 vs 2	1.124	0.429	2.942
smoking 1 vs 2	0.530	0.169	1.667
physically_active 0 vs 1	2.543	1.198	5.395
eGFR_EPI	0.992	0.979	1.006
lipid_lowering	1.711	0.810	3.614
DM_duration	1.096	1.043	1.152

Association of Predicted Probabilities and Observed Responses

**Percent Concordant**	78.4	**Somers' D**	0.571
**Percent Discordant**	21.4	**Gamma**	0.572
**Percent Tied**	0.2	**Tau-a**	0.284
**Pairs**	11858	**c**	0.785

model3 for logIL1beta

The LOGISTIC Procedure

Model Information

**Data Set**	SASUSER.SUN_30DIA1
**Response Variable**	dspn_y
**Number of Response Levels**	2
**Model**	binary logit
**Optimization Technique**	Fisher's scoring

**Number of Observations Read**	219
**Number of Observations Used**	219

Response Profile

Ordered Value	dspn_y	Total Frequency
**1**	0	121
**2**	1	98

Probability modeled is dspn_y=1.

Class Level Information

Class	Value	Design Variables		
**sex**	**1**	1	0	
	**2**	0	1	
**alcohol**	**0**	1	0	0
	**1**	0	1	0
	**2**	0	0	1
**smoking**	**0**	1	0	0
	**1**	0	1	0
	**2**	0	0	1
**physically_active**	**0**	1	0	
	**1**	0	1	

Model Convergence Status

Convergence criterion (GCONV=1E-8) satisfied.

Model Fit Statistics

Criterion	Intercept Only	Intercept and Covariates
**AIC**	303.178	275.384
**SC**	306.568	332.998
**-2 Log L**	301.178	241.384

Testing Global Null Hypothesis: BETA=0

Test	Chi-Square	DF	Pr > ChiSq
**Likelihood Ratio**	59.7942	16	<.0001
**Score**	52.2713	16	<.0001
**Wald**	40.3294	16	0.0007

Type 3 Analysis of Effects

Effect	DF	Wald Chi-Square	Pr > ChiSq
**logIL1beta**	1	3.5886	0.0582
**logIL1beta*sex**	1	0.4811	0.4879
**age**	1	3.8683	0.0492
**sex**	1	0.6511	0.4197
**BMI**	1	0.4863	0.4856
**hypertension**	1	0.9540	0.3287
**logLDL_C**	1	1.0775	0.2993
**HbA1c**	1	3.6404	0.0564
**alcohol**	2	1.6002	0.4493
**smoking**	2	1.6126	0.4465
**physically_active**	1	6.6008	0.0102
**eGFR_EPI**	1	1.1647	0.2805
**lipid_lowering**	1	1.7535	0.1854
**DM_duration**	1	12.8511	0.0003

Analysis of Maximum Likelihood Estimates

Parameter		DF	Estimate	Standard Error	Wald Chi-Square	Pr > ChiSq
**Intercept**		1	-6.0300	2.3391	6.6459	0.0099
**logIL1beta**		1	-0.3381	0.1890	3.1988	0.0737
**logIL1beta*sex**	**1**	1	0.1816	0.2619	0.4811	0.4879
**logIL1beta*sex**	**2**	0	0	.	.	.
**age**		1	0.0382	0.0194	3.8683	0.0492

Analysis of Maximum Likelihood Estimates

Parameter		DF	Estimate	Standard Error	Wald Chi-Square	Pr > ChiSq
sex	1	1	0.3775	0.4678	0.6511	0.4197
sex	2	0	0	.	.	.
BMI		1	0.0326	0.0468	0.4863	0.4856
hypertension		1	-0.3728	0.3817	0.9540	0.3287
logLDL_C		1	-0.5142	0.4954	1.0775	0.2993
HbA1c		1	0.1980	0.1038	3.6404	0.0564
alcohol	0	1	0.5058	0.5067	0.9964	0.3182
alcohol	1	1	0.9073	0.8458	1.1508	0.2834
alcohol	2	0	0	.	.	.
smoking	0	1	0.1071	0.4916	0.0475	0.8275
smoking	1	1	-0.6031	0.5874	1.0541	0.3046
smoking	2	0	0	.	.	.
physically_active	0	1	0.9880	0.3846	6.6008	0.0102
physically_active	1	0	0	.	.	.
eGFR_EPI		1	-0.00754	0.00698	1.1647	0.2805
lipid_lowering		1	0.5045	0.3810	1.7535	0.1854
DM_duration		1	0.0913	0.0255	12.8511	0.0003

Odds Ratio Estimates

Effect	Point Estimate	95% Wald Confidence Limits	
age	1.039	1.000	1.079
BMI	1.033	0.943	1.132
hypertension	0.689	0.326	1.455
logLDL_C	0.598	0.226	1.579
HbA1c	1.219	0.995	1.494
alcohol 0 vs 2	1.658	0.614	4.476
alcohol 1 vs 2	2.478	0.472	13.002
smoking 0 vs 2	1.113	0.425	2.917
smoking 1 vs 2	0.547	0.173	1.730
physically_active 0 vs 1	2.686	1.264	5.707
eGFR_EPI	0.992	0.979	1.006
lipid_lowering	1.656	0.785	3.495
DM_duration	1.096	1.042	1.152

Association of Predicted Probabilities and Observed Responses

**Percent Concordant**	79.1	**Somers' D**	0.584
**Percent Discordant**	20.7	**Gamma**	0.585
**Percent Tied**	0.2	**Tau-a**	0.290
**Pairs**	11858	**c**	0.792

model3 for leptin

The LOGISTIC Procedure

Model Information

**Data Set**	SASUSER.SUN_30DIA1
**Response Variable**	dspn_y
**Number of Response Levels**	2
**Model**	binary logit
**Optimization Technique**	Fisher's scoring

**Number of Observations Read**	219
**Number of Observations Used**	219

Response Profile

Ordered Value	dspn_y	Total Frequency
**1**	0	121
**2**	1	98

Probability modeled is dspn_y=1.

Class Level Information

Class	Value	Design Variables		
**sex**	**1**	1	0	
	**2**	0	1	
**alcohol**	**0**	1	0	0
	**1**	0	1	0
	**2**	0	0	1
**smoking**	**0**	1	0	0
	**1**	0	1	0
	**2**	0	0	1
**physically_active**	**0**	1	0	
	**1**	0	1	

Model Convergence Status

Convergence criterion (GCONV=1E-8) satisfied.

Model Fit Statistics

Criterion	Intercept Only	Intercept and Covariates
AIC	303.178	275.389
SC	306.568	333.003
-2 Log L	301.178	241.389

Testing Global Null Hypothesis: BETA=0

Test	Chi-Square	DF	Pr > ChiSq
Likelihood Ratio	59.7898	16	<.0001
Score	52.7329	16	<.0001
Wald	40.7228	16	0.0006

Type 3 Analysis of Effects

Effect	DF	Wald Chi-Square	Pr > ChiSq
Leptin	1	0.4259	0.5140
Leptin*sex	1	2.9630	0.0852
age	1	3.6798	0.0551
sex	1	5.0063	0.0253
BMI	1	0.1135	0.7362
hypertension	1	1.8132	0.1781
logLDL_C	1	0.6293	0.4276
HbA1c	1	5.1459	0.0233
alcohol	2	1.6275	0.4432
smoking	2	2.6292	0.2686
physically_active	1	6.2079	0.0127
eGFR_EPI	1	1.5188	0.2178
lipid_lowering	1	2.4540	0.1172
DM_duration	1	12.5626	0.0004

Analysis of Maximum Likelihood Estimates

Parameter		DF	Estimate	Standard Error	Wald Chi-Square	Pr > ChiSq
Intercept		1	-6.6621	2.3760	7.8622	0.0050
Leptin		1	0.0267	0.0183	2.1338	0.1441
Leptin*sex	1	1	-0.0917	0.0533	2.9630	0.0852
Leptin*sex	2	0	0	.	.	.
age		1	0.0369	0.0193	3.6798	0.0551

Analysis of Maximum Likelihood Estimates

Parameter		DF	Estimate	Standard Error	Wald Chi-Square	Pr > ChiSq
sex	1	1	1.3811	0.6173	5.0063	0.0253
sex	2	0	0	.	.	.
BMI		1	0.0180	0.0535	0.1135	0.7362
hypertension		1	-0.5108	0.3794	1.8132	0.1781
logLDL_C		1	-0.4018	0.5065	0.6293	0.4276
HbA1c		1	0.2335	0.1029	5.1459	0.0233
alcohol	0	1	0.5189	0.5123	1.0257	0.3112
alcohol	1	1	0.9206	0.8269	1.2395	0.2656
alcohol	2	0	0	.	.	.
smoking	0	1	0.3350	0.5061	0.4380	0.5081
smoking	1	1	-0.6032	0.5851	1.0626	0.3026
smoking	2	0	0	.	.	.
physically_active	0	1	0.9695	0.3891	6.2079	0.0127
physically_active	1	0	0	.	.	.
eGFR_EPI		1	-0.00863	0.00700	1.5188	0.2178
lipid_lowering		1	0.5998	0.3829	2.4540	0.1172
DM_duration		1	0.0920	0.0260	12.5626	0.0004

Odds Ratio Estimates

Effect	Point Estimate	95% Wald Confidence Limits	
age	1.038	0.999	1.078
BMI	1.018	0.917	1.131
hypertension	0.600	0.285	1.262
logLDL_C	0.669	0.248	1.806
HbA1c	1.263	1.032	1.545
alcohol 0 vs 2	1.680	0.616	4.586
alcohol 1 vs 2	2.511	0.497	12.695
smoking 0 vs 2	1.398	0.518	3.770
smoking 1 vs 2	0.547	0.174	1.722
physically_active 0 vs 1	2.637	1.230	5.653
eGFR_EPI	0.991	0.978	1.005
lipid_lowering	1.822	0.860	3.858
DM_duration	1.096	1.042	1.154

Association of Predicted Probabilities and Observed Responses

**Percent Concordant**	78.9	**Somers' D**	0.580
**Percent Discordant**	20.9	**Gamma**	0.581
**Percent Tied**	0.3	**Tau-a**	0.288
**Pairs**	11858	**c**	0.790

The LOGISTIC Procedure

Model Information

**Data Set**	SASUSER.SUN_30DIA1
**Response Variable**	dspn_y
**Number of Response Levels**	2
**Model**	binary logit
**Optimization Technique**	Fisher's scoring

**Number of Observations Read**	219
**Number of Observations Used**	219

Response Profile

Ordered Value	dspn_y	Total Frequency
**1**	0	121
**2**	1	98

Probability modeled is dspn_y=1.

Class Level Information

Class	Value	Design Variables		
**sex**	**1**	1	0	
	**2**	0	1	
**alcohol**	**0**	1	0	0
	**1**	0	1	0
	**2**	0	0	1
**smoking**	**0**	1	0	0
	**1**	0	1	0
	**2**	0	0	1
**physically_active**	**0**	1	0	
	**1**	0	1	

Model Convergence Status

Convergence criterion (GCONV=1E-8) satisfied.

Model Fit Statistics

Criterion	Intercept Only	Intercept and Covariates
AIC	303.178	277.776
SC	306.568	335.390
-2 Log L	301.178	243.776

Testing Global Null Hypothesis: BETA=0

Test	Chi-Square	DF	Pr > ChiSq
Likelihood Ratio	57.4023	16	<.0001
Score	51.0458	16	<.0001
Wald	39.9267	16	0.0008

Type 3 Analysis of Effects

Effect	DF	Wald Chi-Square	Pr > ChiSq
logtnfa	1	1.7491	0.1860
logtnfa*sex	1	0.5280	0.4674
age	1	4.2185	0.0400
sex	1	0.0051	0.9432
BMI	1	0.5436	0.4609
hypertension	1	0.9982	0.3177
logLDL_C	1	1.4581	0.2272
HbA1c	1	4.3811	0.0363
alcohol	2	1.2731	0.5291
smoking	2	1.7633	0.4141
physically_active	1	6.2387	0.0125
eGFR_EPI	1	1.4399	0.2302
lipid_lowering	1	1.9459	0.1630
DM_duration	1	13.6413	0.0002

Analysis of Maximum Likelihood Estimates

Parameter		DF	Estimate	Standard Error	Wald Chi-Square	Pr > ChiSq
Intercept		1	-5.6272	2.3385	5.7905	0.0161
logtnfa		1	-0.3207	0.2383	1.8100	0.1785
logtnfa*sex	1	1	0.2351	0.3235	0.5280	0.4674
logtnfa*sex	2	0	0	.	.	.
age		1	0.0403	0.0196	4.2185	0.0400

Analysis of Maximum Likelihood Estimates

Parameter		DF	Estimate	Standard Error	Wald Chi-Square	Pr > ChiSq
sex	1	1	-0.0655	0.9187	0.0051	0.9432
sex	2	0	0	.	.	.
BMI		1	0.0341	0.0462	0.5436	0.4609
hypertension		1	-0.3822	0.3826	0.9982	0.3177
logLDL_C		1	-0.5928	0.4909	1.4581	0.2272
HbA1c		1	0.2155	0.1029	4.3811	0.0363
alcohol	0	1	0.4922	0.5062	0.9454	0.3309
alcohol	1	1	0.7151	0.8074	0.7843	0.3758
alcohol	2	0	0	.	.	.
smoking	0	1	0.1356	0.4921	0.0759	0.7829
smoking	1	1	-0.6043	0.5832	1.0736	0.3001
smoking	2	0	0	.	.	.
physically_active	0	1	0.9548	0.3823	6.2387	0.0125
physically_active	1	0	0	.	.	.
eGFR_EPI		1	-0.00826	0.00688	1.4399	0.2302
lipid_lowering		1	0.5313	0.3809	1.9459	0.1630
DM_duration		1	0.0937	0.0254	13.6413	0.0002

Odds Ratio Estimates

Effect	Point Estimate	95% Wald Confidence Limits	
age	1.041	1.002	1.082
BMI	1.035	0.945	1.133
hypertension	0.682	0.322	1.444
logLDL_C	0.553	0.211	1.447
HbA1c	1.240	1.014	1.518
alcohol 0 vs 2	1.636	0.607	4.412
alcohol 1 vs 2	2.044	0.420	9.950
smoking 0 vs 2	1.145	0.437	3.004
smoking 1 vs 2	0.546	0.174	1.714
physically_active 0 vs 1	2.598	1.228	5.496
eGFR_EPI	0.992	0.978	1.005
lipid_lowering	1.701	0.806	3.589
DM_duration	1.098	1.045	1.154

Association of Predicted Probabilities and Observed Responses

**Percent Concordant**	78.3	**Somers' D**	0.567
**Percent Discordant**	21.6	**Gamma**	0.568
**Percent Tied**	0.2	**Tau-a**	0.282
**Pairs**	11858	**c**	0.784

The LOGISTIC Procedure

Model Information

**Data Set**	SASUSER.SUN_30DIA1
**Response Variable**	dspn_y
**Number of Response Levels**	2
**Model**	binary logit
**Optimization Technique**	Fisher's scoring

**Number of Observations Read**	219
**Number of Observations Used**	219

Response Profile

Ordered Value	dspn_y	Total Frequency
**1**	0	121
**2**	1	98

Probability modeled is dspn_y=1.

Class Level Information

Class	Value	Design Variables		
**sex**	**1**	1	0	
	**2**	0	1	
**alcohol**	**0**	1	0	0
	**1**	0	1	0
	**2**	0	0	1
**smoking**	**0**	1	0	0
	**1**	0	1	0
	**2**	0	0	1
**physically_active**	**0**	1	0	
	**1**	0	1	

Model Convergence Status

Convergence criterion (GCONV=1E-8) satisfied.

Model Fit Statistics

Criterion	Intercept Only	Intercept and Covariates
AIC	303.178	272.171
SC	306.568	329.785
-2 Log L	301.178	238.171

Testing Global Null Hypothesis: BETA=0

Test	Chi-Square	DF	Pr > ChiSq
Likelihood Ratio	63.0079	16	<.0001
Score	55.0784	16	<.0001
Wald	41.8789	16	0.0004

Type 3 Analysis of Effects

Effect	DF	Wald Chi-Square	Pr > ChiSq
loghsCRP	1	6.5919	0.0102
loghsCRP*sex	1	0.6587	0.4170
age	1	3.1470	0.0761
sex	1	0.8084	0.3686
BMI	1	1.3724	0.2414
hypertension	1	0.2842	0.5940
logLDL_C	1	0.9620	0.3267
HbA1c	1	7.1520	0.0075
alcohol	2	1.9415	0.3788
smoking	2	1.9222	0.3825
physically_active	1	7.2136	0.0072
eGFR_EPI	1	3.4609	0.0628
lipid_lowering	1	1.2517	0.2632
DM_duration	1	12.9726	0.0003

Analysis of Maximum Likelihood Estimates

Parameter		DF	Estimate	Standard Error	Wald Chi-Square	Pr > ChiSq
Intercept		1	-6.9925	2.3638	8.7507	0.0031
loghsCRP		1	-0.6139	0.2468	6.1874	0.0129
loghsCRP*sex	1	1	0.2603	0.3208	0.6587	0.4170
loghsCRP*sex	2	0	0	.	.	.
age		1	0.0341	0.0192	3.1470	0.0761

Analysis of Maximum Likelihood Estimates

Parameter		DF	Estimate	Standard Error	Wald Chi-Square	Pr > ChiSq
sex	1	1	0.4042	0.4496	0.8084	0.3686
sex	2	0	0	.	.	.
BMI		1	0.0565	0.0482	1.3724	0.2414
hypertension		1	-0.2087	0.3914	0.2842	0.5940
logLDL_C		1	-0.4867	0.4962	0.9620	0.3267
HbA1c		1	0.2960	0.1107	7.1520	0.0075
alcohol	0	1	0.5785	0.5191	1.2417	0.2651
alcohol	1	1	1.0065	0.8511	1.3983	0.2370
alcohol	2	0	0	.	.	.
smoking	0	1	0.1049	0.4984	0.0443	0.8333
smoking	1	1	-0.6636	0.5982	1.2306	0.2673
smoking	2	0	0	.	.	.
physically_active	0	1	1.0703	0.3985	7.2136	0.0072
physically_active	1	0	0	.	.	.
eGFR_EPI		1	-0.0138	0.00740	3.4609	0.0628
lipid_lowering		1	0.4311	0.3853	1.2517	0.2632
DM_duration		1	0.0937	0.0260	12.9726	0.0003

Odds Ratio Estimates

Effect	Point Estimate	95% Wald Confidence Limits	
age	1.035	0.996	1.074
BMI	1.058	0.963	1.163
hypertension	0.812	0.377	1.748
logLDL_C	0.615	0.232	1.626
HbA1c	1.344	1.082	1.670
alcohol 0 vs 2	1.783	0.645	4.933
alcohol 1 vs 2	2.736	0.516	14.508
smoking 0 vs 2	1.111	0.418	2.949
smoking 1 vs 2	0.515	0.159	1.663
physically_active 0 vs 1	2.916	1.335	6.368
eGFR_EPI	0.986	0.972	1.001
lipid_lowering	1.539	0.723	3.275
DM_duration	1.098	1.044	1.156

Association of Predicted Probabilities and Observed Responses

**Percent Concordant**	79.6	**Somers' D**	0.594
**Percent Discordant**	20.2	**Gamma**	0.595
**Percent Tied**	0.2	**Tau-a**	0.295
**Pairs**	11858	**c**	0.797

model3 for logl2n

The LOGISTIC Procedure

Model Information

**Data Set**	SASUSER.SUN_30DIA1
**Response Variable**	dspn_y
**Number of Response Levels**	2
**Model**	binary logit
**Optimization Technique**	Fisher's scoring

**Number of Observations Read**	219
**Number of Observations Used**	219

Response Profile

Ordered Value	dspn_y	Total Frequency
**1**	0	121
**2**	1	98

Probability modeled is dspn_y=1.

Class Level Information

Class	Value	Design Variables		
**sex**	**1**	1	0	
	**2**	0	1	
**alcohol**	**0**	1	0	0
	**1**	0	1	0
	**2**	0	0	1
**smoking**	**0**	1	0	0
	**1**	0	1	0
	**2**	0	0	1
**physically_active**	**0**	1	0	
	**1**	0	1	

Model Convergence Status

Convergence criterion (GCONV=1E-8) satisfied.

Model Fit Statistics

Criterion	Intercept Only	Intercept and Covariates
**AIC**	303.178	278.312
**SC**	306.568	335.926
**-2 Log L**	301.178	244.312

Testing Global Null Hypothesis: BETA=0

Test	Chi-Square	DF	Pr > ChiSq
**Likelihood Ratio**	56.8670	16	<.0001
**Score**	50.7257	16	<.0001
**Wald**	39.7837	16	0.0008

Type 3 Analysis of Effects

Effect	DF	Wald Chi-Square	Pr > ChiSq
**logl2n**	1	0.1888	0.6639
**logl2n*sex**	1	1.4054	0.2358
**age**	1	3.8739	0.0490
**sex**	1	0.9012	0.3425
**BMI**	1	0.4183	0.5178
**hypertension**	1	1.3020	0.2538
**logLDL_C**	1	1.6724	0.1959
**HbA1c**	1	4.1218	0.0423
**alcohol**	2	1.3514	0.5088
**smoking**	2	1.6319	0.4422
**physically_active**	1	5.6474	0.0175
**eGFR_EPI**	1	1.7241	0.1892
**lipid_lowering**	1	1.6447	0.1997
**DM_duration**	1	14.0185	0.0002

Analysis of Maximum Likelihood Estimates

Parameter		DF	Estimate	Standard Error	Wald Chi-Square	Pr > ChiSq
**Intercept**		1	-4.1497	3.0021	1.9107	0.1669
**logl2n**		1	-0.3884	0.3431	1.2818	0.2576
**logl2n*sex**	**1**	1	0.5670	0.4783	1.4054	0.2358
**logl2n*sex**	**2**	0	0	.	.	.
**age**		1	0.0377	0.0192	3.8739	0.0490

Analysis of Maximum Likelihood Estimates

Parameter		DF	Estimate	Standard Error	Wald Chi-Square	Pr > ChiSq
sex	1	1	-2.1861	2.3029	0.9012	0.3425
sex	2	0	0	.	.	.
BMI		1	0.0301	0.0465	0.4183	0.5178
hypertension		1	-0.4333	0.3797	1.3020	0.2538
logLDL_C		1	-0.6381	0.4934	1.6724	0.1959
HbA1c		1	0.2081	0.1025	4.1218	0.0423
alcohol	0	1	0.5203	0.5088	1.0454	0.3066
alcohol	1	1	0.7057	0.7929	0.7922	0.3734
alcohol	2	0	0	.	.	.
smoking	0	1	0.1562	0.4922	0.1007	0.7510
smoking	1	1	-0.5563	0.5811	0.9167	0.3383
smoking	2	0	0	.	.	.
physically_active	0	1	0.9120	0.3838	5.6474	0.0175
physically_active	1	0	0	.	.	.
eGFR_EPI		1	-0.00916	0.00697	1.7241	0.1892
lipid_lowering		1	0.4874	0.3800	1.6447	0.1997
DM_duration		1	0.0960	0.0256	14.0185	0.0002

Odds Ratio Estimates

Effect	Point Estimate	95% Wald Confidence Limits	
age	1.038	1.000	1.078
BMI	1.031	0.941	1.129
hypertension	0.648	0.308	1.365
logLDL_C	0.528	0.201	1.390
HbA1c	1.231	1.007	1.505
alcohol 0 vs 2	1.682	0.621	4.561
alcohol 1 vs 2	2.025	0.428	9.581
smoking 0 vs 2	1.169	0.446	3.068
smoking 1 vs 2	0.573	0.184	1.791
physically_active 0 vs 1	2.489	1.173	5.282
eGFR_EPI	0.991	0.977	1.005
lipid_lowering	1.628	0.773	3.429
DM_duration	1.101	1.047	1.157

Association of Predicted Probabilities and Observed Responses

**Percent Concordant**	77.8	**Somers' D**	0.557
**Percent Discordant**	22.1	**Gamma**	0.557
**Percent Tied**	0.1	**Tau-a**	0.277
**Pairs**	11858	**c**	0.778

model3 for logadi

The LOGISTIC Procedure

Model Information

**Data Set**	SASUSER.SUN_30DIA1
**Response Variable**	dspn_y
**Number of Response Levels**	2
**Model**	binary logit
**Optimization Technique**	Fisher's scoring

**Number of Observations Read**	219
**Number of Observations Used**	219

Response Profile

Ordered Value	dspn_y	Total Frequency
**1**	0	121
**2**	1	98

Probability modeled is dspn_y=1.

Class Level Information

Class	Value	Design Variables		
**sex**	**1**	1	0	
	**2**	0	1	
**alcohol**	**0**	1	0	0
	**1**	0	1	0
	**2**	0	0	1
**smoking**	**0**	1	0	0
	**1**	0	1	0
	**2**	0	0	1
**physically_active**	**0**	1	0	
	**1**	0	1	

Model Convergence Status

Convergence criterion (GCONV=1E-8) satisfied.

Model Fit Statistics

Criterion	Intercept Only	Intercept and Covariates
AIC	303.178	277.130
SC	306.568	334.744
-2 Log L	301.178	243.130

Testing Global Null Hypothesis: BETA=0

Test	Chi-Square	DF	Pr > ChiSq
Likelihood Ratio	58.0482	16	<.0001
Score	51.1204	16	<.0001
Wald	38.9945	16	0.0011

Type 3 Analysis of Effects

Effect	DF	Wald Chi-Square	Pr > ChiSq
logadi	1	0.7901	0.3741
logadi*age_2g	1	0.0224	0.8812
age_2g	1	0.2010	0.6539
sex	1	2.4573	0.1170
BMI	1	0.1209	0.7281
hypertension	1	0.2761	0.5992
logLDL_C	1	1.1438	0.2849
HbA1c	1	4.1481	0.0417
alcohol	2	2.2693	0.3215
smoking	2	1.5120	0.4695
physically_active	1	5.9832	0.0144
eGFR_EPI	1	0.5442	0.4607
lipid_lowering	1	2.2676	0.1321
DM_duration	1	13.1663	0.0003

Analysis of Maximum Likelihood Estimates

Parameter		DF	Estimate	Standard Error	Wald Chi-Square	Pr > ChiSq
Intercept		1	-6.2483	2.7633	5.1128	0.0238
logadi		1	0.6975	0.7847	0.7901	0.3741
logadi*age_2g		1	-0.0735	0.4915	0.0224	0.8812
age_2g		1	0.5081	1.1332	0.2010	0.6539
sex	1	1	0.7199	0.4592	2.4573	0.1170

Analysis of Maximum Likelihood Estimates

Parameter		DF	Estimate	Standard Error	Wald Chi-Square	Pr > ChiSq
sex	2	0	0	.	.	.
BMI		1	0.0160	0.0460	0.1209	0.7281
hypertension		1	-0.1968	0.3746	0.2761	0.5992
logLDL_C		1	-0.5359	0.5011	1.1438	0.2849
HbA1c		1	0.2055	0.1009	4.1481	0.0417
alcohol	0	1	0.7336	0.5124	2.0493	0.1523
alcohol	1	1	0.7661	0.7940	0.9308	0.3347
alcohol	2	0	0	.	.	.
smoking	0	1	0.1484	0.4990	0.0884	0.7662
smoking	1	1	-0.5434	0.5856	0.8612	0.3534
smoking	2	0	0	.	.	.
physically_active	0	1	0.9369	0.3830	5.9832	0.0144
physically_active	1	0	0	.	.	.
eGFR_EPI		1	-0.00527	0.00714	0.5442	0.4607
lipid_lowering		1	0.5726	0.3803	2.2676	0.1321
DM_duration		1	0.0918	0.0253	13.1663	0.0003

Odds Ratio Estimates

Effect	Point Estimate	95% Wald Confidence Limits	
sex 1 vs 2	2.054	0.835	5.053
BMI	1.016	0.928	1.112
hypertension	0.821	0.394	1.711
logLDL_C	0.585	0.219	1.562
HbA1c	1.228	1.008	1.497
alcohol 0 vs 2	2.082	0.763	5.685
alcohol 1 vs 2	2.151	0.454	10.199
smoking 0 vs 2	1.160	0.436	3.084
smoking 1 vs 2	0.581	0.184	1.830
physically_active 0 vs 1	2.552	1.205	5.406
eGFR_EPI	0.995	0.981	1.009
lipid_lowering	1.773	0.841	3.736
DM_duration	1.096	1.043	1.152

Association of Predicted Probabilities and Observed Responses

**Percent Concordant**	77.6	**Somers' D**	0.554
**Percent Discordant**	22.2	**Gamma**	0.556
**Percent Tied**	0.2	**Tau-a**	0.275
**Pairs**	11858	**c**	0.777

The LOGISTIC Procedure

Model Information

**Data Set**	SASUSER.SUN_30DIA1
**Response Variable**	dspn_y
**Number of Response Levels**	2
**Model**	binary logit
**Optimization Technique**	Fisher's scoring

**Number of Observations Read**	219
**Number of Observations Used**	219

Response Profile

Ordered Value	dspn_y	Total Frequency
**1**	0	121
**2**	1	98

Probability modeled is dspn_y=1.

Class Level Information

Class	Value	Design Variables		
**sex**	**1**	1	0	
	**2**	0	1	
**alcohol**	**0**	1	0	0
	**1**	0	1	0
	**2**	0	0	1
**smoking**	**0**	1	0	0
	**1**	0	1	0
	**2**	0	0	1
**physically_active**	**0**	1	0	
	**1**	0	1	

Model Convergence Status

Convergence criterion (GCONV=1E-8) satisfied.

Model Fit Statistics

Criterion	Intercept Only	Intercept and Covariates
AIC	303.178	280.417
SC	306.568	338.031
-2 Log L	301.178	246.417

Testing Global Null Hypothesis: BETA=0

Test	Chi-Square	DF	Pr > ChiSq
Likelihood Ratio	54.7616	16	<.0001
Score	49.2962	16	<.0001
Wald	39.1509	16	0.0010

Type 3 Analysis of Effects

Effect	DF	Wald Chi-Square	Pr > ChiSq
logIL6	1	1.7310	0.1883
logIL6*age_2g	1	2.2035	0.1377
age_2g	1	3.4976	0.0615
sex	1	0.8002	0.3710
BMI	1	0.1344	0.7139
hypertension	1	0.5606	0.4540
logLDL_C	1	1.0746	0.2999
HbA1c	1	3.0471	0.0809
alcohol	2	1.3609	0.5064
smoking	2	1.0422	0.5939
physically_active	1	6.1939	0.0128
eGFR_EPI	1	1.1701	0.2794
lipid_lowering	1	1.8845	0.1698
DM_duration	1	15.5851	<.0001

Analysis of Maximum Likelihood Estimates

Parameter		DF	Estimate	Standard Error	Wald Chi-Square	Pr > ChiSq
Intercept		1	-4.9809	2.0382	5.9718	0.0145
logIL6		1	0.3111	0.2365	1.7310	0.1883
logIL6*age_2g		1	-0.2360	0.1590	2.2035	0.1377
age_2g		1	0.9618	0.5143	3.4976	0.0615
sex	1	1	0.3956	0.4423	0.8002	0.3710

Analysis of Maximum Likelihood Estimates

Parameter		DF	Estimate	Standard Error	Wald Chi-Square	Pr > ChiSq
sex	2	0	0	.	.	.
BMI		1	0.0166	0.0454	0.1344	0.7139
hypertension		1	-0.2763	0.3690	0.5606	0.4540
logLDL_C		1	-0.5111	0.4930	1.0746	0.2999
HbA1c		1	0.1775	0.1017	3.0471	0.0809
alcohol	0	1	0.5288	0.5094	1.0777	0.2992
alcohol	1	1	0.6909	0.7904	0.7641	0.3820
alcohol	2	0	0	.	.	.
smoking	0	1	0.2170	0.4907	0.1955	0.6584
smoking	1	1	-0.3564	0.5734	0.3863	0.5343
smoking	2	0	0	.	.	.
physically_active	0	1	0.9565	0.3843	6.1939	0.0128
physically_active	1	0	0	.	.	.
eGFR_EPI		1	-0.00750	0.00694	1.1701	0.2794
lipid_lowering		1	0.5189	0.3780	1.8845	0.1698
DM_duration		1	0.1012	0.0256	15.5851	<.0001

Odds Ratio Estimates

Effect	Point Estimate	95% Wald Confidence Limits	
sex 1 vs 2	1.485	0.624	3.534
BMI	1.017	0.930	1.111
hypertension	0.759	0.368	1.563
logLDL_C	0.600	0.228	1.577
HbA1c	1.194	0.978	1.458
alcohol 0 vs 2	1.697	0.625	4.606
alcohol 1 vs 2	1.996	0.424	9.394
smoking 0 vs 2	1.242	0.475	3.250
smoking 1 vs 2	0.700	0.228	2.154
physically_active 0 vs 1	2.603	1.225	5.528
eGFR_EPI	0.993	0.979	1.006
lipid_lowering	1.680	0.801	3.525
DM_duration	1.107	1.052	1.164

Association of Predicted Probabilities and Observed Responses

**Percent Concordant**	77.4	**Somers' D**	0.551
**Percent Discordant**	22.3	**Gamma**	0.552
**Percent Tied**	0.2	**Tau-a**	0.274
**Pairs**	11858	**c**	0.775

The LOGISTIC Procedure

Model Information

**Data Set**	SASUSER.SUN_30DIA1
**Response Variable**	dspn_y
**Number of Response Levels**	2
**Model**	binary logit
**Optimization Technique**	Fisher's scoring

**Number of Observations Read**	219
**Number of Observations Used**	219

Response Profile

Ordered Value	dspn_y	Total Frequency
**1**	0	121
**2**	1	98

Probability modeled is dspn_y=1.

Class Level Information

Class	Value	Design Variables		
**sex**	**1**	1	0	
	**2**	0	1	
**alcohol**	**0**	1	0	0
	**1**	0	1	0
	**2**	0	0	1
**smoking**	**0**	1	0	0
	**1**	0	1	0
	**2**	0	0	1
**physically_active**	**0**	1	0	
	**1**	0	1	

Model Convergence Status

Convergence criterion (GCONV=1E-8) satisfied.

Model Fit Statistics

Criterion	Intercept Only	Intercept and Covariates
**AIC**	303.178	275.255
**SC**	306.568	332.869
**-2 Log L**	301.178	241.255

Testing Global Null Hypothesis: BETA=0

Test	Chi-Square	DF	Pr > ChiSq
**Likelihood Ratio**	59.9233	16	<.0001
**Score**	53.0597	16	<.0001
**Wald**	41.0953	16	0.0005

Type 3 Analysis of Effects

Effect	DF	Wald Chi-Square	Pr > ChiSq
**logIL1beta**	1	1.0091	0.3151
**logIL1beta*age_2g**	1	2.8968	0.0888
**age_2g**	1	2.7596	0.0967
**sex**	1	0.7426	0.3888
**BMI**	1	0.1821	0.6696
**hypertension**	1	0.5064	0.4767
**logLDL_C**	1	1.0918	0.2961
**HbA1c**	1	2.6222	0.1054
**alcohol**	2	1.5848	0.4528
**smoking**	2	1.3329	0.5135
**physically_active**	1	6.9021	0.0086
**eGFR_EPI**	1	1.0532	0.3048
**lipid_lowering**	1	1.3940	0.2377
**DM_duration**	1	15.6976	<.0001

Analysis of Maximum Likelihood Estimates

Parameter		DF	Estimate	Standard Error	Wald Chi-Square	Pr > ChiSq
**Intercept**		1	-4.4231	1.9931	4.9250	0.0265
**logIL1beta**		1	0.3746	0.3729	1.0091	0.3151
**logIL1beta*age_2g**		1	-0.4528	0.2660	2.8968	0.0888
**age_2g**		1	0.6359	0.3828	2.7596	0.0967
**sex**	**1**	1	0.3869	0.4490	0.7426	0.3888

Analysis of Maximum Likelihood Estimates

Parameter		DF	Estimate	Standard Error	Wald Chi-Square	Pr > ChiSq
sex	2	0	0	.	.	.
BMI		1	0.0197	0.0461	0.1821	0.6696
hypertension		1	-0.2634	0.3701	0.5064	0.4767
logLDL_C		1	-0.5226	0.5001	1.0918	0.2961
HbA1c		1	0.1675	0.1034	2.6222	0.1054
alcohol	0	1	0.5089	0.5096	0.9972	0.3180
alcohol	1	1	0.8898	0.8286	1.1533	0.2829
alcohol	2	0	0	.	.	.
smoking	0	1	0.1719	0.4945	0.1209	0.7281
smoking	1	1	-0.4930	0.5882	0.7026	0.4019
smoking	2	0	0	.	.	.
physically_active	0	1	1.0178	0.3874	6.9021	0.0086
physically_active	1	0	0	.	.	.
eGFR_EPI		1	-0.00726	0.00707	1.0532	0.3048
lipid_lowering		1	0.4524	0.3832	1.3940	0.2377
DM_duration		1	0.1026	0.0259	15.6976	<.0001

Odds Ratio Estimates

Effect	Point Estimate	95% Wald Confidence Limits	
sex 1 vs 2	1.472	0.611	3.550
BMI	1.020	0.932	1.116
hypertension	0.768	0.372	1.587
logLDL_C	0.593	0.223	1.580
HbA1c	1.182	0.965	1.448
alcohol 0 vs 2	1.664	0.613	4.517
alcohol 1 vs 2	2.435	0.480	12.352
smoking 0 vs 2	1.188	0.451	3.130
smoking 1 vs 2	0.611	0.193	1.934
physically_active 0 vs 1	2.767	1.295	5.913
eGFR_EPI	0.993	0.979	1.007
lipid_lowering	1.572	0.742	3.332
DM_duration	1.108	1.053	1.166

Association of Predicted Probabilities and Observed Responses

**Percent Concordant**	78.9	**Somers' D**	0.579
**Percent Discordant**	21.0	**Gamma**	0.580
**Percent Tied**	0.1	**Tau-a**	0.288
**Pairs**	11858	**c**	0.790

model3 for leptin

The LOGISTIC Procedure

Model Information

**Data Set**	SASUSER.SUN_30DIA1
**Response Variable**	dspn_y
**Number of Response Levels**	2
**Model**	binary logit
**Optimization Technique**	Fisher's scoring

**Number of Observations Read**	219
**Number of Observations Used**	219

Response Profile

Ordered Value	dspn_y	Total Frequency
**1**	0	121
**2**	1	98

Probability modeled is dspn_y=1.

Class Level Information

Class	Value	Design Variables		
**sex**	**1**	1	0	
	**2**	0	1	
**alcohol**	**0**	1	0	0
	**1**	0	1	0
	**2**	0	0	1
**smoking**	**0**	1	0	0
	**1**	0	1	0
	**2**	0	0	1
**physically_active**	**0**	1	0	
	**1**	0	1	

Model Convergence Status

Convergence criterion (GCONV=1E-8) satisfied.

Model Fit Statistics

Criterion	Intercept Only	Intercept and Covariates
AIC	303.178	278.247
SC	306.568	335.861
-2 Log L	301.178	244.247

Testing Global Null Hypothesis: BETA=0

Test	Chi-Square	DF	Pr > ChiSq
Likelihood Ratio	56.9313	16	<.0001
Score	51.0812	16	<.0001
Wald	40.4074	16	0.0007

Type 3 Analysis of Effects

Effect	DF	Wald Chi-Square	Pr > ChiSq
Leptin	1	3.5779	0.0586
Leptin*age_2g	1	2.4633	0.1165
age_2g	1	3.2083	0.0733
sex	1	2.5155	0.1127
BMI	1	0.0573	0.8108
hypertension	1	0.8540	0.3554
logLDL_C	1	0.4221	0.5159
HbA1c	1	4.1332	0.0420
alcohol	2	0.8955	0.6391
smoking	2	1.5073	0.4707
physically_active	1	5.1421	0.0234
eGFR_EPI	1	0.8987	0.3431
lipid_lowering	1	2.2818	0.1309
DM_duration	1	13.0696	0.0003

Analysis of Maximum Likelihood Estimates

Parameter		DF	Estimate	Standard Error	Wald Chi-Square	Pr > ChiSq
Intercept		1	-4.8809	2.0831	5.4901	0.0191
Leptin		1	0.0989	0.0523	3.5779	0.0586
Leptin*age_2g		1	-0.0440	0.0281	2.4633	0.1165
age_2g		1	0.8534	0.4764	3.2083	0.0733
sex	1	1	0.7848	0.4948	2.5155	0.1127

Analysis of Maximum Likelihood Estimates

Parameter		DF	Estimate	Standard Error	Wald Chi-Square	Pr > ChiSq
**sex**	**2**	0	0	.	.	.
**BMI**		1	-0.0125	0.0521	0.0573	0.8108
**hypertension**		1	-0.3443	0.3726	0.8540	0.3554
**logLDL_C**		1	-0.3271	0.5035	0.4221	0.5159
**HbA1c**		1	0.2059	0.1013	4.1332	0.0420
**alcohol**	**0**	1	0.4367	0.5078	0.7396	0.3898
**alcohol**	**1**	1	0.5401	0.7848	0.4736	0.4913
**alcohol**	**2**	0	0	.	.	.
**smoking**	**0**	1	0.2463	0.5088	0.2343	0.6284
**smoking**	**1**	1	-0.4418	0.5702	0.6004	0.4384
**smoking**	**2**	0	0	.	.	.
**physically_active**	**0**	1	0.8634	0.3808	5.1421	0.0234
**physically_active**	**1**	0	0	.	.	.
**eGFR_EPI**		1	-0.00660	0.00696	0.8987	0.3431
**lipid_lowering**		1	0.5808	0.3845	2.2818	0.1309
**DM_duration**		1	0.0922	0.0255	13.0696	0.0003

Odds Ratio Estimates

Effect	Point Estimate	95% Wald Confidence Limits	
**sex 1 vs 2**	2.192	0.831	5.782
**BMI**	0.988	0.892	1.094
**hypertension**	0.709	0.341	1.471
**logLDL_C**	0.721	0.269	1.934
**HbA1c**	1.229	1.007	1.498
**alcohol 0 vs 2**	1.548	0.572	4.187
**alcohol 1 vs 2**	1.716	0.369	7.991
**smoking 0 vs 2**	1.279	0.472	3.468
**smoking 1 vs 2**	0.643	0.210	1.966
**physically_active 0 vs 1**	2.371	1.124	5.001
**eGFR_EPI**	0.993	0.980	1.007
**lipid_lowering**	1.787	0.841	3.797
**DM_duration**	1.097	1.043	1.153

Association of Predicted Probabilities and Observed Responses

**Percent Concordant**	77.3	**Somers' D**	0.547
**Percent Discordant**	22.6	**Gamma**	0.547
**Percent Tied**	0.2	**Tau-a**	0.272
**Pairs**	11858	**c**	0.773

The LOGISTIC Procedure

Model Information

**Data Set**	SASUSER.SUN_30DIA1
**Response Variable**	dspn_y
**Number of Response Levels**	2
**Model**	binary logit
**Optimization Technique**	Fisher's scoring

**Number of Observations Read**	219
**Number of Observations Used**	219

Response Profile

Ordered Value	dspn_y	Total Frequency
**1**	0	121
**2**	1	98

Probability modeled is dspn_y=1.

Class Level Information

Class	Value	Design Variables		
**sex**	**1**	1	0	
	**2**	0	1	
**alcohol**	**0**	1	0	0
	**1**	0	1	0
	**2**	0	0	1
**smoking**	**0**	1	0	0
	**1**	0	1	0
	**2**	0	0	1
**physically_active**	**0**	1	0	
	**1**	0	1	

Model Convergence Status

Convergence criterion (GCONV=1E-8) satisfied.

Model Fit Statistics

Criterion	Intercept Only	Intercept and Covariates
AIC	303.178	277.775
SC	306.568	335.389
-2 Log L	301.178	243.775

Testing Global Null Hypothesis: BETA=0

Test	Chi-Square	DF	Pr > ChiSq
Likelihood Ratio	57.4036	16	<.0001
Score	51.4791	16	<.0001
Wald	40.6494	16	0.0006

Type 3 Analysis of Effects

Effect	DF	Wald Chi-Square	Pr > ChiSq
logtnfa	1	1.5460	0.2137
logtnfa*age_2g	1	3.0380	0.0813
age_2g	1	4.1894	0.0407
sex	1	0.5376	0.4634
BMI	1	0.2604	0.6098
hypertension	1	0.6320	0.4266
logLDL_C	1	1.4351	0.2309
HbA1c	1	3.6419	0.0563
alcohol	2	1.2279	0.5412
smoking	2	1.2138	0.5450
physically_active	1	6.1026	0.0135
eGFR_EPI	1	0.9888	0.3200
lipid_lowering	1	1.8536	0.1734
DM_duration	1	16.2653	<.0001

Analysis of Maximum Likelihood Estimates

Parameter		DF	Estimate	Standard Error	Wald Chi-Square	Pr > ChiSq
Intercept		1	-5.8798	2.2856	6.6182	0.0101
logtnfa		1	0.5757	0.4630	1.5460	0.2137
logtnfa*age_2g		1	-0.5476	0.3142	3.0380	0.0813
age_2g		1	1.7660	0.8628	4.1894	0.0407
sex	1	1	0.3316	0.4522	0.5376	0.4634

Analysis of Maximum Likelihood Estimates

Parameter		DF	Estimate	Standard Error	Wald Chi-Square	Pr > ChiSq
**sex**	**2**	0	0	.	.	.
**BMI**		1	0.0233	0.0456	0.2604	0.6098
**hypertension**		1	-0.2937	0.3695	0.6320	0.4266
**logLDL_C**		1	-0.5952	0.4968	1.4351	0.2309
**HbA1c**		1	0.1951	0.1022	3.6419	0.0563
**alcohol**	**0**	1	0.5232	0.5091	1.0558	0.3042
**alcohol**	**1**	1	0.6139	0.8089	0.5760	0.4479
**alcohol**	**2**	0	0	.	.	.
**smoking**	**0**	1	0.1424	0.4957	0.0825	0.7739
**smoking**	**1**	1	-0.4727	0.5832	0.6568	0.4177
**smoking**	**2**	0	0	.	.	.
**physically_active**	**0**	1	0.9482	0.3838	6.1026	0.0135
**physically_active**	**1**	0	0	.	.	.
**eGFR_EPI**		1	-0.00692	0.00696	0.9888	0.3200
**lipid_lowering**		1	0.5182	0.3806	1.8536	0.1734
**DM_duration**		1	0.1034	0.0256	16.2653	<.0001

Odds Ratio Estimates

Effect	Point Estimate	95% Wald Confidence Limits	
**sex 1 vs 2**	1.393	0.574	3.380
**BMI**	1.024	0.936	1.119
**hypertension**	0.745	0.361	1.538
**logLDL_C**	0.551	0.208	1.460
**HbA1c**	1.215	0.995	1.485
**alcohol 0 vs 2**	1.687	0.622	4.577
**alcohol 1 vs 2**	1.848	0.379	9.018
**smoking 0 vs 2**	1.153	0.436	3.046
**smoking 1 vs 2**	0.623	0.199	1.955
**physically_active 0 vs 1**	2.581	1.216	5.477
**eGFR_EPI**	0.993	0.980	1.007
**lipid_lowering**	1.679	0.796	3.540
**DM_duration**	1.109	1.055	1.166

Association of Predicted Probabilities and Observed Responses

**Percent Concordant**	78.5	**Somers' D**	0.571
**Percent Discordant**	21.3	**Gamma**	0.572
**Percent Tied**	0.2	**Tau-a**	0.284
**Pairs**	11858	**c**	0.786

model3 for loghsCRP

The LOGISTIC Procedure

Model Information

**Data Set**	SASUSER.SUN_30DIA1
**Response Variable**	dspn_y
**Number of Response Levels**	2
**Model**	binary logit
**Optimization Technique**	Fisher's scoring

**Number of Observations Read**	219
**Number of Observations Used**	219

Response Profile

Ordered Value	dspn_y	Total Frequency
**1**	0	121
**2**	1	98

Probability modeled is dspn_y=1.

Class Level Information

Class	Value	Design Variables		
**sex**	**1**	1	0	
	**2**	0	1	
**alcohol**	**0**	1	0	0
	**1**	0	1	0
	**2**	0	0	1
**smoking**	**0**	1	0	0
	**1**	0	1	0
	**2**	0	0	1
**physically_active**	**0**	1	0	
	**1**	0	1	

Model Convergence Status

Convergence criterion (GCONV=1E-8) satisfied.

Model Fit Statistics

Criterion	Intercept Only	Intercept and Covariates
**AIC**	303.178	274.979
**SC**	306.568	332.593
**-2 Log L**	301.178	240.979

Testing Global Null Hypothesis: BETA=0

Test	Chi-Square	DF	Pr > ChiSq
**Likelihood Ratio**	60.1999	16	<.0001
**Score**	53.0194	16	<.0001
**Wald**	41.0683	16	0.0005

Type 3 Analysis of Effects

Effect	DF	Wald Chi-Square	Pr > ChiSq
**loghsCRP**	1	0.1673	0.6826
**loghsCRP*age_2g**	1	0.3071	0.5795
**age_2g**	1	0.8718	0.3505
**sex**	1	0.7197	0.3962
**BMI**	1	0.9533	0.3289
**hypertension**	1	0.0730	0.7870
**logLDL_C**	1	0.5831	0.4451
**HbA1c**	1	6.5803	0.0103
**alcohol**	2	2.3247	0.3128
**smoking**	2	1.5365	0.4638
**physically_active**	1	6.6138	0.0101
**eGFR_EPI**	1	3.5451	0.0597
**lipid_lowering**	1	1.3032	0.2536
**DM_duration**	1	14.4389	0.0001

Analysis of Maximum Likelihood Estimates

Parameter		DF	Estimate	Standard Error	Wald Chi-Square	Pr > ChiSq
**Intercept**		1	-5.3525	2.0264	6.9766	0.0083
**loghsCRP**		1	-0.2189	0.5353	0.1673	0.6826
**loghsCRP*age_2g**		1	-0.1789	0.3229	0.3071	0.5795
**age_2g**		1	0.3344	0.3581	0.8718	0.3505
**sex**	**1**	1	0.3771	0.4445	0.7197	0.3962

Analysis of Maximum Likelihood Estimates

Parameter		DF	Estimate	Standard Error	Wald Chi-Square	Pr > ChiSq
**sex**	**2**	0	0	.	.	.
**BMI**		1	0.0461	0.0472	0.9533	0.3289
**hypertension**		1	-0.1029	0.3807	0.0730	0.7870
**logLDL_C**		1	-0.3806	0.4985	0.5831	0.4451
**HbA1c**		1	0.2789	0.1087	6.5803	0.0103
**alcohol**	**0**	1	0.6682	0.5201	1.6506	0.1989
**alcohol**	**1**	1	1.0308	0.8407	1.5034	0.2202
**alcohol**	**2**	0	0	.	.	.
**smoking**	**0**	1	0.1309	0.5042	0.0674	0.7951
**smoking**	**1**	1	-0.5516	0.5850	0.8890	0.3458
**smoking**	**2**	0	0	.	.	.
**physically_active**	**0**	1	1.0173	0.3956	6.6138	0.0101
**physically_active**	**1**	0	0	.	.	.
**eGFR_EPI**		1	-0.0139	0.00739	3.5451	0.0597
**lipid_lowering**		1	0.4382	0.3839	1.3032	0.2536
**DM_duration**		1	0.0976	0.0257	14.4389	0.0001

Odds Ratio Estimates

Effect	Point Estimate	95% Wald Confidence Limits	
**sex 1 vs 2**	1.458	0.610	3.484
**BMI**	1.047	0.955	1.149
**hypertension**	0.902	0.428	1.903
**logLDL_C**	0.683	0.257	1.816
**HbA1c**	1.322	1.068	1.636
**alcohol 0 vs 2**	1.951	0.704	5.406
**alcohol 1 vs 2**	2.803	0.540	14.563
**smoking 0 vs 2**	1.140	0.424	3.062
**smoking 1 vs 2**	0.576	0.183	1.813
**physically_active 0 vs 1**	2.766	1.274	6.005
**eGFR_EPI**	0.986	0.972	1.001
**lipid_lowering**	1.550	0.730	3.289
**DM_duration**	1.102	1.048	1.159

Association of Predicted Probabilities and Observed Responses

**Percent Concordant**	79.2	**Somers' D**	0.588
**Percent Discordant**	20.5	**Gamma**	0.589
**Percent Tied**	0.3	**Tau-a**	0.292
**Pairs**	11858	**c**	0.794

model3 for logl2n

The LOGISTIC Procedure

Model Information

**Data Set**	SASUSER.SUN_30DIA1
**Response Variable**	dspn_y
**Number of Response Levels**	2
**Model**	binary logit
**Optimization Technique**	Fisher's scoring

**Number of Observations Read**	219
**Number of Observations Used**	219

Response Profile

Ordered Value	dspn_y	Total Frequency
**1**	0	121
**2**	1	98

Probability modeled is dspn_y=1.

Class Level Information

Class	Value	Design Variables		
**sex**	**1**	1	0	
	**2**	0	1	
**alcohol**	**0**	1	0	0
	**1**	0	1	0
	**2**	0	0	1
**smoking**	**0**	1	0	0
	**1**	0	1	0
	**2**	0	0	1
**physically_active**	**0**	1	0	
	**1**	0	1	

Model Convergence Status

Convergence criterion (GCONV=1E-8) satisfied.

Model Fit Statistics

Criterion	Intercept Only	Intercept and Covariates
AIC	303.178	282.132
SC	306.568	339.747
-2 Log L	301.178	248.132

Testing Global Null Hypothesis: BETA=0

Test	Chi-Square	DF	Pr > ChiSq
Likelihood Ratio	53.0462	16	<.0001
Score	47.8113	16	<.0001
Wald	38.2173	16	0.0014

Type 3 Analysis of Effects

Effect	DF	Wald Chi-Square	Pr > ChiSq
logl2n	1	0.1242	0.7246
logl2n*age_2g	1	0.3256	0.5682
age_2g	1	0.5359	0.4641
sex	1	1.0218	0.3121
BMI	1	0.1723	0.6780
hypertension	1	0.7871	0.3750
logLDL_C	1	1.2476	0.2640
HbA1c	1	3.7988	0.0513
alcohol	2	1.2212	0.5430
smoking	2	1.1092	0.5743
physically_active	1	5.5962	0.0180
eGFR_EPI	1	1.5418	0.2143
lipid_lowering	1	1.9799	0.1594
DM_duration	1	15.2058	<.0001

Analysis of Maximum Likelihood Estimates

Parameter		DF	Estimate	Standard Error	Wald Chi-Square	Pr > ChiSq
Intercept		1	-5.3609	4.0193	1.7790	0.1823
logl2n		1	0.2533	0.7189	0.1242	0.7246
logl2n*age_2g		1	-0.2660	0.4662	0.3256	0.5682
age_2g		1	1.6334	2.2312	0.5359	0.4641
sex	1	1	0.4422	0.4374	1.0218	0.3121

Analysis of Maximum Likelihood Estimates

Parameter		DF	Estimate	Standard Error	Wald Chi-Square	Pr > ChiSq
sex	2	0	0	.	.	.
BMI		1	0.0188	0.0453	0.1723	0.6780
hypertension		1	-0.3263	0.3678	0.7871	0.3750
logLDL_C		1	-0.5507	0.4930	1.2476	0.2640
HbA1c		1	0.1959	0.1005	3.7988	0.0513
alcohol	0	1	0.5039	0.5055	0.9937	0.3188
alcohol	1	1	0.6379	0.7849	0.6606	0.4164
alcohol	2	0	0	.	.	.
smoking	0	1	0.2158	0.4903	0.1937	0.6598
smoking	1	1	-0.3676	0.5679	0.4190	0.5174
smoking	2	0	0	.	.	.
physically_active	0	1	0.9029	0.3817	5.5962	0.0180
physically_active	1	0	0	.	.	.
eGFR_EPI		1	-0.00865	0.00697	1.5418	0.2143
lipid_lowering		1	0.5291	0.3761	1.9799	0.1594
DM_duration		1	0.0982	0.0252	15.2058	<.0001

Odds Ratio Estimates

Effect	Point Estimate	95% Wald Confidence Limits	
sex 1 vs 2	1.556	0.660	3.668
BMI	1.019	0.932	1.114
hypertension	0.722	0.351	1.484
logLDL_C	0.577	0.219	1.515
HbA1c	1.216	0.999	1.481
alcohol 0 vs 2	1.655	0.615	4.458
alcohol 1 vs 2	1.892	0.406	8.812
smoking 0 vs 2	1.241	0.475	3.244
smoking 1 vs 2	0.692	0.227	2.107
physically_active 0 vs 1	2.467	1.167	5.212
eGFR_EPI	0.991	0.978	1.005
lipid_lowering	1.697	0.812	3.547
DM_duration	1.103	1.050	1.159

Association of Predicted Probabilities and Observed Responses

**Percent Concordant**	76.8	**Somers' D**	0.538
**Percent Discordant**	23.0	**Gamma**	0.538
**Percent Tied**	0.2	**Tau-a**	0.267
**Pairs**	11858	**c**	0.769

The LOGISTIC Procedure

Model Information

**Data Set**	SASUSER.SUN_30DIA1
**Response Variable**	dspn_y
**Number of Response Levels**	2
**Model**	binary logit
**Optimization Technique**	Fisher's scoring

**Number of Observations Read**	219
**Number of Observations Used**	219

Response Profile

Ordered Value	dspn_y	Total Frequency
**1**	0	121
**2**	1	98

Probability modeled is dspn_y=1.

Class Level Information

Class	Value	Design Variables		
**sex**	**1**	1	0	
	**2**	0	1	
**alcohol**	**0**	1	0	0
	**1**	0	1	0
	**2**	0	0	1
**smoking**	**0**	1	0	0
	**1**	0	1	0
	**2**	0	0	1
**physically_active**	**0**	1	0	
	**1**	0	1	

Model Convergence Status

Convergence criterion (GCONV=1E-8) satisfied.

Model Fit Statistics

Criterion	Intercept Only	Intercept and Covariates
AIC	303.178	268.129
SC	306.568	329.132
-2 Log L	301.178	232.129

Testing Global Null Hypothesis: BETA=0

Test	Chi-Square	DF	Pr > ChiSq
Likelihood Ratio	69.0499	17	<.0001
Score	59.6624	17	<.0001
Wald	43.4793	17	0.0004

Type 3 Analysis of Effects

Effect	DF	Wald Chi-Square	Pr > ChiSq
logadi	1	2.2773	0.1313
logadi*dn_y	1	0.7151	0.3978
dn_y	1	0.0042	0.9481
age	1	5.1503	0.0232
sex	1	2.4525	0.1173
BMI	1	0.2573	0.6120
hypertension	1	0.5435	0.4610
logLDL_C	1	0.9473	0.3304
HbA1c	1	2.5616	0.1095
alcohol	2	1.4118	0.4937
smoking	2	1.9556	0.3761
physically_active	1	5.2035	0.0225
eGFR_EPI	1	0.2020	0.6531
lipid_lowering	1	1.7852	0.1815
DM_duration	1	9.0125	0.0027

Analysis of Maximum Likelihood Estimates

Parameter	DF	Estimate	Standard Error	Wald Chi-Square	Pr > ChiSq
Intercept	1	-8.4709	2.5606	10.9435	0.0009
logadi	1	0.4100	0.2717	2.2773	0.1313
logadi*dn_y	1	0.7125	0.8426	0.7151	0.3978
dn_y	1	-0.1270	1.9516	0.0042	0.9481

Analysis of Maximum Likelihood Estimates

Parameter		DF	Estimate	Standard Error	Wald Chi-Square	Pr > ChiSq
age		1	0.0487	0.0215	5.1503	0.0232
sex	1	1	0.7443	0.4753	2.4525	0.1173
sex	2	0	0	.	.	.
BMI		1	0.0247	0.0486	0.2573	0.6120
hypertension		1	-0.2880	0.3907	0.5435	0.4610
logLDL_C		1	-0.4997	0.5134	0.9473	0.3304
HbA1c		1	0.1749	0.1093	2.5616	0.1095
alcohol	0	1	0.6057	0.5268	1.3218	0.2503
alcohol	1	1	0.5764	0.7993	0.5201	0.4708
alcohol	2	0	0	.	.	.
smoking	0	1	-0.1131	0.5111	0.0490	0.8248
smoking	1	1	-0.8044	0.6045	1.7706	0.1833
smoking	2	0	0	.	.	.
physically_active	0	1	0.8909	0.3905	5.2035	0.0225
physically_active	1	0	0	.	.	.
eGFR_EPI		1	0.00357	0.00795	0.2020	0.6531
lipid_lowering		1	0.5251	0.3930	1.7852	0.1815
DM_duration		1	0.0785	0.0261	9.0125	0.0027

Odds Ratio Estimates

Effect	Point Estimate	95% Wald Confidence Limits	
age	1.050	1.007	1.095
sex 1 vs 2	2.105	0.829	5.343
BMI	1.025	0.932	1.128
hypertension	0.750	0.349	1.612
logLDL_C	0.607	0.222	1.660
HbA1c	1.191	0.961	1.476
alcohol 0 vs 2	1.833	0.653	5.146
alcohol 1 vs 2	1.780	0.372	8.525
smoking 0 vs 2	0.893	0.328	2.432
smoking 1 vs 2	0.447	0.137	1.463
physically_active 0 vs 1	2.437	1.134	5.240
eGFR_EPI	1.004	0.988	1.019
lipid_lowering	1.691	0.783	3.653

Odds Ratio Estimates

Effect	Point Estimate	95% Wald Confidence Limits	
**DM_duration**	1.082	1.028	1.139

Association of Predicted Probabilities and Observed Responses

**Percent Concordant**	80.1	**Somers' D**	0.604
**Percent Discordant**	19.7	**Gamma**	0.605
**Percent Tied**	0.1	**Tau-a**	0.300
**Pairs**	11858	**c**	0.802

The LOGISTIC Procedure

Model Information

**Data Set**	SASUSER.SUN_30DIA1
**Response Variable**	dspn_y
**Number of Response Levels**	2
**Model**	binary logit
**Optimization Technique**	Fisher's scoring

**Number of Observations Read**	219
**Number of Observations Used**	219

Response Profile

Ordered Value	dspn_y	Total Frequency
**1**	0	121
**2**	1	98

Probability modeled is dspn_y=1.

Class Level Information

Class	Value	Design Variables		
**sex**	**1**	1	0	
	**2**	0	1	
**alcohol**	**0**	1	0	0
	**1**	0	1	0
	**2**	0	0	1
**smoking**	**0**	1	0	0
	**1**	0	1	0
	**2**	0	0	1
**physically_active**	**0**	1	0	
	**1**	0	1	

Model Convergence Status

Convergence criterion (GCONV=1E-8) satisfied.

Model Fit Statistics

Criterion	Intercept Only	Intercept and Covariates
**AIC**	303.178	271.828
**SC**	306.568	332.831
**-2 Log L**	301.178	235.828

Testing Global Null Hypothesis: BETA=0

Test	Chi-Square	DF	Pr > ChiSq
**Likelihood Ratio**	65.3507	17	<.0001
**Score**	56.8110	17	<.0001
**Wald**	42.3200	17	0.0006

Type 3 Analysis of Effects

Effect	DF	Wald Chi-Square	Pr > ChiSq
**logIL6**	1	0.4071	0.5234
**logIL6*dn_y**	1	0.7112	0.3991
**dn_y**	1	1.5558	0.2123
**age**	1	6.8079	0.0091
**sex**	1	0.8557	0.3549
**BMI**	1	0.3637	0.5464
**hypertension**	1	1.0016	0.3169
**logLDL_C**	1	0.9727	0.3240
**HbA1c**	1	2.1388	0.1436
**alcohol**	2	0.7001	0.7047
**smoking**	2	1.5484	0.4611
**physically_active**	1	5.1636	0.0231
**eGFR_EPI**	1	0.0046	0.9460
**lipid_lowering**	1	1.5408	0.2145
**DM_duration**	1	10.2120	0.0014

Analysis of Maximum Likelihood Estimates

Parameter	DF	Estimate	Standard Error	Wald Chi-Square	Pr > ChiSq
**Intercept**	1	-7.1113	2.4159	8.6644	0.0032
**logIL6**	1	-0.0563	0.0883	0.4071	0.5234
**logIL6*dn_y**	1	0.2160	0.2562	0.7112	0.3991
**dn_y**	1	1.0065	0.8070	1.5558	0.2123

Analysis of Maximum Likelihood Estimates

Parameter		DF	Estimate	Standard Error	Wald Chi-Square	Pr > ChiSq
age		1	0.0532	0.0204	6.8079	0.0091
sex	1	1	0.4290	0.4638	0.8557	0.3549
sex	2	0	0	.	.	.
BMI		1	0.0287	0.0475	0.3637	0.5464
hypertension		1	-0.3849	0.3846	1.0016	0.3169
logLDL_C		1	-0.5025	0.5095	0.9727	0.3240
HbA1c		1	0.1591	0.1088	2.1388	0.1436
alcohol	0	1	0.3866	0.5294	0.5333	0.4652
alcohol	1	1	0.5257	0.8067	0.4246	0.5146
alcohol	2	0	0	.	.	.
smoking	0	1	-0.0425	0.5092	0.0070	0.9335
smoking	1	1	-0.6808	0.5945	1.3115	0.2521
smoking	2	0	0	.	.	.
physically_active	0	1	0.8858	0.3898	5.1636	0.0231
physically_active	1	0	0	.	.	.
eGFR_EPI		1	0.000517	0.00764	0.0046	0.9460
lipid_lowering		1	0.4833	0.3894	1.5408	0.2145
DM_duration		1	0.0826	0.0259	10.2120	0.0014

Odds Ratio Estimates

Effect	Point Estimate	95% Wald Confidence Limits	
age	1.055	1.013	1.098
sex 1 vs 2	1.536	0.619	3.811
BMI	1.029	0.938	1.130
hypertension	0.681	0.320	1.446
logLDL_C	0.605	0.223	1.642
HbA1c	1.172	0.947	1.451
alcohol 0 vs 2	1.472	0.522	4.155
alcohol 1 vs 2	1.692	0.348	8.222
smoking 0 vs 2	0.958	0.353	2.600
smoking 1 vs 2	0.506	0.158	1.623
physically_active 0 vs 1	2.425	1.129	5.206
eGFR_EPI	1.001	0.986	1.016
lipid_lowering	1.621	0.756	3.478

Odds Ratio Estimates

Effect	Point Estimate	95% Wald Confidence Limits	
**DM_duration**	1.086	1.032	1.143

Association of Predicted Probabilities and Observed Responses

**Percent Concordant**	79.8	**Somers' D**	0.599
**Percent Discordant**	20.0	**Gamma**	0.600
**Percent Tied**	0.2	**Tau-a**	0.297
**Pairs**	11858	**c**	0.799

model3 for logIL1beta

The LOGISTIC Procedure

Model Information

**Data Set**	SASUSER.SUN_30DIA1
**Response Variable**	dspn_y
**Number of Response Levels**	2
**Model**	binary logit
**Optimization Technique**	Fisher's scoring

**Number of Observations Read**	219
**Number of Observations Used**	219

Response Profile

Ordered Value	dspn_y	Total Frequency
**1**	0	121
**2**	1	98

Probability modeled is dspn_y=1.

Class Level Information

Class	Value	Design Variables		
**sex**	**1**	1	0	
	**2**	0	1	
**alcohol**	**0**	1	0	0
	**1**	0	1	0
	**2**	0	0	1
**smoking**	**0**	1	0	0
	**1**	0	1	0
	**2**	0	0	1
**physically_active**	**0**	1	0	
	**1**	0	1	

Model Convergence Status

Convergence criterion (GCONV=1E-8) satisfied.

Model Fit Statistics

Criterion	Intercept Only	Intercept and Covariates
AIC	303.178	268.016
SC	306.568	329.020
-2 Log L	301.178	232.016

Testing Global Null Hypothesis: BETA=0

Test	Chi-Square	DF	Pr > ChiSq
Likelihood Ratio	69.1622	17	<.0001
Score	59.2879	17	<.0001
Wald	43.2715	17	0.0004

Type 3 Analysis of Effects

Effect	DF	Wald Chi-Square	Pr > ChiSq
logIL1beta	1	3.5028	0.0613
logIL1beta*dn_y	1	0.0027	0.9586
dn_y	1	7.0777	0.0078
age	1	6.2920	0.0121
sex	1	0.9925	0.3191
BMI	1	0.4529	0.5009
hypertension	1	1.0024	0.3167
logLDL_C	1	0.6881	0.4068
HbA1c	1	1.3312	0.2486
alcohol	2	1.1775	0.5550
smoking	2	1.6907	0.4294
physically_active	1	5.6006	0.0180
eGFR_EPI	1	0.1288	0.7197
lipid_lowering	1	1.6552	0.1983
DM_duration	1	10.1365	0.0015

Analysis of Maximum Likelihood Estimates

Parameter	DF	Estimate	Standard Error	Wald Chi-Square	Pr > ChiSq
Intercept	1	-7.2217	2.4562	8.6447	0.0033
logIL1beta	1	-0.2868	0.1532	3.5028	0.0613
logIL1beta*dn_y	1	0.0191	0.3679	0.0027	0.9586
dn_y	1	1.6053	0.6034	7.0777	0.0078

Analysis of Maximum Likelihood Estimates

Parameter		DF	Estimate	Standard Error	Wald Chi-Square	Pr > ChiSq
age		1	0.0522	0.0208	6.2920	0.0121
sex	1	1	0.4640	0.4657	0.9925	0.3191
sex	2	0	0	.	.	.
BMI		1	0.0326	0.0484	0.4529	0.5009
hypertension		1	-0.3871	0.3867	1.0024	0.3167
logLDL_C		1	-0.4262	0.5138	0.6881	0.4068
HbA1c		1	0.1293	0.1120	1.3312	0.2486
alcohol	0	1	0.4367	0.5238	0.6949	0.4045
alcohol	1	1	0.8062	0.8472	0.9055	0.3413
alcohol	2	0	0	.	.	.
smoking	0	1	-0.1337	0.5119	0.0682	0.7939
smoking	1	1	-0.7501	0.5969	1.5792	0.2089
smoking	2	0	0	.	.	.
physically_active	0	1	0.9275	0.3919	5.6006	0.0180
physically_active	1	0	0	.	.	.
eGFR_EPI		1	0.00281	0.00783	0.1288	0.7197
lipid_lowering		1	0.5035	0.3914	1.6552	0.1983
DM_duration		1	0.0835	0.0262	10.1365	0.0015

Odds Ratio Estimates

Effect	Point Estimate	95% Wald Confidence Limits	
age	1.054	1.011	1.097
sex 1 vs 2	1.590	0.638	3.962
BMI	1.033	0.940	1.136
hypertension	0.679	0.318	1.449
logLDL_C	0.653	0.239	1.787
HbA1c	1.138	0.914	1.417
alcohol 0 vs 2	1.548	0.554	4.321
alcohol 1 vs 2	2.239	0.426	11.783
smoking 0 vs 2	0.875	0.321	2.386
smoking 1 vs 2	0.472	0.147	1.522
physically_active 0 vs 1	2.528	1.173	5.450
eGFR_EPI	1.003	0.988	1.018
lipid_lowering	1.655	0.768	3.563

Odds Ratio Estimates

Effect	Point Estimate	95% Wald Confidence Limits	
**DM_duration**	1.087	1.033	1.144

Association of Predicted Probabilities and Observed Responses

**Percent Concordant**	80.8	**Somers' D**	0.618
**Percent Discordant**	19.0	**Gamma**	0.619
**Percent Tied**	0.2	**Tau-a**	0.307
**Pairs**	11858	**c**	0.809

The LOGISTIC Procedure

Model Information

**Data Set**	SASUSER.SUN_30DIA1
**Response Variable**	dspn_y
**Number of Response Levels**	2
**Model**	binary logit
**Optimization Technique**	Fisher's scoring

**Number of Observations Read**	219
**Number of Observations Used**	219

Response Profile

Ordered Value	dspn_y	Total Frequency
**1**	0	121
**2**	1	98

Probability modeled is dspn_y=1.

Class Level Information

Class	Value	Design Variables		
**sex**	**1**	1	0	
	**2**	0	1	
**alcohol**	**0**	1	0	0
	**1**	0	1	0
	**2**	0	0	1
**smoking**	**0**	1	0	0
	**1**	0	1	0
	**2**	0	0	1
**physically_active**	**0**	1	0	
	**1**	0	1	

Model Convergence Status

Convergence criterion (GCONV=1E-8) satisfied.

Model Fit Statistics

Criterion	Intercept Only	Intercept and Covariates
AIC	303.178	270.254
SC	306.568	331.257
-2 Log L	301.178	234.254

Testing Global Null Hypothesis: BETA=0

Test	Chi-Square	DF	Pr > ChiSq
Likelihood Ratio	66.9246	17	<.0001
Score	58.8775	17	<.0001
Wald	44.1965	17	0.0003

Type 3 Analysis of Effects

Effect	DF	Wald Chi-Square	Pr > ChiSq
Leptin	1	2.1503	0.1425
Leptin*dn_y	1	0.0412	0.8392
dn_y	1	4.0983	0.0429
age	1	5.7530	0.0165
sex	1	2.7345	0.0982
BMI	1	0.0385	0.8444
hypertension	1	1.2214	0.2691
logLDL_C	1	0.3551	0.5512
HbA1c	1	2.3121	0.1284
alcohol	2	0.5091	0.7753
smoking	2	1.8130	0.4039
physically_active	1	4.2704	0.0388
eGFR_EPI	1	0.1604	0.6888
lipid_lowering	1	2.2662	0.1322
DM_duration	1	7.9492	0.0048

Analysis of Maximum Likelihood Estimates

Parameter	DF	Estimate	Standard Error	Wald Chi-Square	Pr > ChiSq
Intercept	1	-6.8805	2.4313	8.0089	0.0047
Leptin	1	0.0262	0.0179	2.1503	0.1425
Leptin*dn_y	1	0.00887	0.0437	0.0412	0.8392
dn_y	1	1.5127	0.7472	4.0983	0.0429

Analysis of Maximum Likelihood Estimates

Parameter		DF	Estimate	Standard Error	Wald Chi-Square	Pr > ChiSq
age		1	0.0497	0.0207	5.7530	0.0165
sex	1	1	0.8520	0.5152	2.7345	0.0982
sex	2	0	0	.	.	.
BMI		1	-0.0108	0.0552	0.0385	0.8444
hypertension		1	-0.4258	0.3853	1.2214	0.2691
logLDL_C		1	-0.3128	0.5250	0.3551	0.5512
HbA1c		1	0.1656	0.1089	2.3121	0.1284
alcohol	0	1	0.3578	0.5222	0.4694	0.4933
alcohol	1	1	0.3583	0.7882	0.2067	0.6494
alcohol	2	0	0	.	.	.
smoking	0	1	0.0738	0.5174	0.0204	0.8865
smoking	1	1	-0.6711	0.5926	1.2826	0.2574
smoking	2	0	0	.	.	.
physically_active	0	1	0.8080	0.3910	4.2704	0.0388
physically_active	1	0	0	.	.	.
eGFR_EPI		1	0.00312	0.00779	0.1604	0.6888
lipid_lowering		1	0.5931	0.3940	2.2662	0.1322
DM_duration		1	0.0747	0.0265	7.9492	0.0048

Odds Ratio Estimates

Effect	Point Estimate	95% Wald Confidence Limits	
age	1.051	1.009	1.095
sex 1 vs 2	2.344	0.854	6.436
BMI	0.989	0.888	1.102
hypertension	0.653	0.307	1.390
logLDL_C	0.731	0.261	2.046
HbA1c	1.180	0.953	1.461
alcohol 0 vs 2	1.430	0.514	3.980
alcohol 1 vs 2	1.431	0.305	6.707
smoking 0 vs 2	1.077	0.391	2.968
smoking 1 vs 2	0.511	0.160	1.633
physically_active 0 vs 1	2.243	1.043	4.827
eGFR_EPI	1.003	0.988	1.019
lipid_lowering	1.810	0.836	3.917

Odds Ratio Estimates

Effect	Point Estimate	95% Wald Confidence Limits	
DM_duration	1.078	1.023	1.135

Association of Predicted Probabilities and Observed Responses

Percent Concordant	80.1	Somers' D	0.603
Percent Discordant	19.8	Gamma	0.603
Percent Tied	0.1	Tau-a	0.299
Pairs	11858	c	0.801

The LOGISTIC Procedure

Model Information

**Data Set**	SASUSER.SUN_30DIA1
**Response Variable**	dspn_y
**Number of Response Levels**	2
**Model**	binary logit
**Optimization Technique**	Fisher's scoring

**Number of Observations Read**	219
**Number of Observations Used**	219

Response Profile

Ordered Value	dspn_y	Total Frequency
**1**	0	121
**2**	1	98

Probability modeled is dspn_y=1.

Class Level Information

Class	Value	Design Variables		
**sex**	**1**	1	0	
	**2**	0	1	
**alcohol**	**0**	1	0	0
	**1**	0	1	0
	**2**	0	0	1
**smoking**	**0**	1	0	0
	**1**	0	1	0
	**2**	0	0	1
**physically_active**	**0**	1	0	
	**1**	0	1	

Model Convergence Status

Convergence criterion (GCONV=1E-8) satisfied.

Model Fit Statistics

Criterion	Intercept Only	Intercept and Covariates
AIC	303.178	270.148
SC	306.568	331.151
-2 Log L	301.178	234.148

Testing Global Null Hypothesis: BETA=0

Test	Chi-Square	DF	Pr > ChiSq
Likelihood Ratio	67.0303	17	<.0001
Score	58.0709	17	<.0001
Wald	42.8329	17	0.0005

Type 3 Analysis of Effects

Effect	DF	Wald Chi-Square	Pr > ChiSq
logtnfa	1	2.3365	0.1264
logtnfa*dn_y	1	0.0890	0.7654
dn_y	1	0.8318	0.3617
age	1	6.6133	0.0101
sex	1	1.2256	0.2683
BMI	1	0.4770	0.4898
hypertension	1	1.0599	0.3032
logLDL_C	1	0.9807	0.3220
HbA1c	1	2.0903	0.1482
alcohol	2	0.7611	0.6835
smoking	2	1.7952	0.4075
physically_active	1	5.2496	0.0220
eGFR_EPI	1	0.0494	0.8242
lipid_lowering	1	1.9274	0.1650
DM_duration	1	10.7134	0.0011

Analysis of Maximum Likelihood Estimates

Parameter	DF	Estimate	Standard Error	Wald Chi-Square	Pr > ChiSq
Intercept	1	-6.8921	2.4273	8.0622	0.0045
logtnfa	1	-0.2651	0.1734	2.3365	0.1264
logtnfa*dn_y	1	0.1384	0.4639	0.0890	0.7654
dn_y	1	1.2536	1.3745	0.8318	0.3617

Analysis of Maximum Likelihood Estimates

Parameter		DF	Estimate	Standard Error	Wald Chi-Square	Pr > ChiSq
age		1	0.0532	0.0207	6.6133	0.0101
sex	1	1	0.5133	0.4637	1.2256	0.2683
sex	2	0	0	.	.	.
BMI		1	0.0329	0.0477	0.4770	0.4898
hypertension		1	-0.3958	0.3844	1.0599	0.3032
logLDL_C		1	-0.5028	0.5077	0.9807	0.3220
HbA1c		1	0.1589	0.1099	2.0903	0.1482
alcohol	0	1	0.4208	0.5237	0.6455	0.4217
alcohol	1	1	0.5073	0.8168	0.3857	0.5346
alcohol	2	0	0	.	.	.
smoking	0	1	-0.0943	0.5090	0.0343	0.8531
smoking	1	1	-0.7572	0.5971	1.6082	0.2047
smoking	2	0	0	.	.	.
physically_active	0	1	0.8952	0.3907	5.2496	0.0220
physically_active	1	0	0	.	.	.
eGFR_EPI		1	0.00171	0.00771	0.0494	0.8242
lipid_lowering		1	0.5435	0.3915	1.9274	0.1650
DM_duration		1	0.0856	0.0261	10.7134	0.0011

Odds Ratio Estimates

Effect	Point Estimate	95% Wald Confidence Limits	
age	1.055	1.013	1.098
sex 1 vs 2	1.671	0.673	4.146
BMI	1.033	0.941	1.135
hypertension	0.673	0.317	1.430
logLDL_C	0.605	0.224	1.636
HbA1c	1.172	0.945	1.454
alcohol 0 vs 2	1.523	0.546	4.252
alcohol 1 vs 2	1.661	0.335	8.234
smoking 0 vs 2	0.910	0.336	2.468
smoking 1 vs 2	0.469	0.146	1.511
physically_active 0 vs 1	2.448	1.138	5.264
eGFR_EPI	1.002	0.987	1.017
lipid_lowering	1.722	0.799	3.709

Odds Ratio Estimates

Effect	Point Estimate	95% Wald Confidence Limits	
DM_duration	1.089	1.035	1.147

Association of Predicted Probabilities and Observed Responses

Percent Concordant	79.9	Somers' D	0.599
Percent Discordant	20.0	Gamma	0.600
Percent Tied	0.2	Tau-a	0.298
Pairs	11858	c	0.800

model3 for loghsCRP

The LOGISTIC Procedure

Model Information

**Data Set**	SASUSER.SUN_30DIA1
**Response Variable**	dspn_y
**Number of Response Levels**	2
**Model**	binary logit
**Optimization Technique**	Fisher's scoring

**Number of Observations Read**	219
**Number of Observations Used**	219

Response Profile

Ordered Value	dspn_y	Total Frequency
**1**	0	121
**2**	1	98

Probability modeled is dspn_y=1.

Class Level Information

Class	Value	Design Variables		
**sex**	**1**	1	0	
	**2**	0	1	
**alcohol**	**0**	1	0	0
	**1**	0	1	0
	**2**	0	0	1
**smoking**	**0**	1	0	0
	**1**	0	1	0
	**2**	0	0	1
**physically_active**	**0**	1	0	
	**1**	0	1	

Model Convergence Status

Convergence criterion (GCONV=1E-8) satisfied.

Model Fit Statistics

Criterion	Intercept Only	Intercept and Covariates
**AIC**	303.178	263.661
**SC**	306.568	324.665
**-2 Log L**	301.178	227.661

Testing Global Null Hypothesis: BETA=0

Test	Chi-Square	DF	Pr > ChiSq
**Likelihood Ratio**	73.5171	17	<.0001
**Score**	62.9642	17	<.0001
**Wald**	44.5872	17	0.0003

Type 3 Analysis of Effects

Effect	DF	Wald Chi-Square	Pr > ChiSq
**loghsCRP**	1	6.5335	0.0106
**loghsCRP*dn_y**	1	0.0855	0.7700
**dn_y**	1	8.2501	0.0041
**age**	1	5.7180	0.0168
**sex**	1	0.7171	0.3971
**BMI**	1	1.3346	0.2480
**hypertension**	1	0.1432	0.7051
**logLDL_C**	1	0.5095	0.4754
**HbA1c**	1	4.4885	0.0341
**alcohol**	2	1.6519	0.4378
**smoking**	2	2.1809	0.3361
**physically_active**	1	6.3074	0.0120
**eGFR_EPI**	1	0.3768	0.5393
**lipid_lowering**	1	0.9877	0.3203
**DM_duration**	1	9.3988	0.0022

Analysis of Maximum Likelihood Estimates

Parameter	DF	Estimate	Standard Error	Wald Chi-Square	Pr > ChiSq
**Intercept**	1	-8.3394	2.4595	11.4970	0.0007
**loghsCRP**	1	-0.5539	0.2167	6.5335	0.0106
**loghsCRP*dn_y**	1	-0.1231	0.4212	0.0855	0.7700
**dn_y**	1	1.8473	0.6431	8.2501	0.0041

Analysis of Maximum Likelihood Estimates

Parameter		DF	Estimate	Standard Error	Wald Chi-Square	Pr > ChiSq
age		1	0.0502	0.0210	5.7180	0.0168
sex	1	1	0.3935	0.4646	0.7171	0.3971
sex	2	0	0	.	.	.
BMI		1	0.0569	0.0492	1.3346	0.2480
hypertension		1	-0.1502	0.3968	0.1432	0.7051
logLDL_C		1	-0.3687	0.5165	0.5095	0.4754
HbA1c		1	0.2475	0.1168	4.4885	0.0341
alcohol	0	1	0.5211	0.5354	0.9475	0.3304
alcohol	1	1	0.9789	0.8502	1.3258	0.2496
alcohol	2	0	0	.	.	.
smoking	0	1	-0.1642	0.5127	0.1026	0.7488
smoking	1	1	-0.8797	0.6188	2.0206	0.1552
smoking	2	0	0	.	.	.
physically_active	0	1	1.0169	0.4049	6.3074	0.0120
physically_active	1	0	0	.	.	.
eGFR_EPI		1	-0.00495	0.00807	0.3768	0.5393
lipid_lowering		1	0.3954	0.3979	0.9877	0.3203
DM_duration		1	0.0810	0.0264	9.3988	0.0022

Odds Ratio Estimates

Effect	Point Estimate	95% Wald Confidence Limits	
age	1.051	1.009	1.096
sex 1 vs 2	1.482	0.596	3.684
BMI	1.059	0.961	1.166
hypertension	0.861	0.395	1.873
logLDL_C	0.692	0.251	1.903
HbA1c	1.281	1.019	1.610
alcohol 0 vs 2	1.684	0.590	4.809
alcohol 1 vs 2	2.662	0.503	14.086
smoking 0 vs 2	0.849	0.311	2.318
smoking 1 vs 2	0.415	0.123	1.395
physically_active 0 vs 1	2.764	1.250	6.113
eGFR_EPI	0.995	0.979	1.011
lipid_lowering	1.485	0.681	3.239

Odds Ratio Estimates

Effect	Point Estimate	95% Wald Confidence Limits	
**DM_duration**	1.084	1.030	1.142

Association of Predicted Probabilities and Observed Responses

**Percent Concordant**	81.5	**Somers' D**	0.631
**Percent Discordant**	18.4	**Gamma**	0.632
**Percent Tied**	0.1	**Tau-a**	0.314
**Pairs**	11858	**c**	0.816

model3 for logl2n

The LOGISTIC Procedure

Model Information

**Data Set**	SASUSER.SUN_30DIA1
**Response Variable**	dspn_y
**Number of Response Levels**	2
**Model**	binary logit
**Optimization Technique**	Fisher's scoring

**Number of Observations Read**	219
**Number of Observations Used**	219

Response Profile

Ordered Value	dspn_y	Total Frequency
**1**	0	121
**2**	1	98

Probability modeled is dspn_y=1.

Class Level Information

Class	Value	Design Variables		
**sex**	**1**	1	0	
	**2**	0	1	
**alcohol**	**0**	1	0	0
	**1**	0	1	0
	**2**	0	0	1
**smoking**	**0**	1	0	0
	**1**	0	1	0
	**2**	0	0	1
**physically_active**	**0**	1	0	
	**1**	0	1	

Model Convergence Status

Convergence criterion (GCONV=1E-8) satisfied.

Model Fit Statistics

Criterion	Intercept Only	Intercept and Covariates
AIC	303.178	271.747
SC	306.568	332.751
-2 Log L	301.178	235.747

Testing Global Null Hypothesis: BETA=0

Test	Chi-Square	DF	Pr > ChiSq
Likelihood Ratio	65.4310	17	<.0001
Score	56.9618	17	<.0001
Wald	42.4633	17	0.0006

Type 3 Analysis of Effects

Effect	DF	Wald Chi-Square	Pr > ChiSq
logl2n	1	0.7786	0.3776
logl2n*dn_y	1	0.4864	0.4855
dn_y	1	0.0506	0.8221
age	1	6.6326	0.0100
sex	1	0.9845	0.3211
BMI	1	0.3890	0.5328
hypertension	1	1.2792	0.2581
logLDL_C	1	1.0868	0.2972
HbA1c	1	2.3319	0.1267
alcohol	2	0.6137	0.7358
smoking	2	1.5739	0.4552
physically_active	1	4.8422	0.0278
eGFR_EPI	1	0.0192	0.8897
lipid_lowering	1	1.6912	0.1934
DM_duration	1	9.9212	0.0016

Analysis of Maximum Likelihood Estimates

Parameter	DF	Estimate	Standard Error	Wald Chi-Square	Pr > ChiSq
Intercept	1	-6.2010	2.8534	4.7228	0.0298
logl2n	1	-0.2390	0.2709	0.7786	0.3776
logl2n*dn_y	1	0.4807	0.6892	0.4864	0.4855
dn_y	1	-0.7542	3.3533	0.0506	0.8221

Analysis of Maximum Likelihood Estimates

Parameter		DF	Estimate	Standard Error	Wald Chi-Square	Pr > ChiSq
age		1	0.0534	0.0207	6.6326	0.0100
sex	1	1	0.4578	0.4614	0.9845	0.3211
sex	2	0	0	.	.	.
BMI		1	0.0297	0.0476	0.3890	0.5328
hypertension		1	-0.4340	0.3837	1.2792	0.2581
logLDL_C		1	-0.5308	0.5092	1.0868	0.2972
HbA1c		1	0.1683	0.1102	2.3319	0.1267
alcohol	0	1	0.3714	0.5301	0.4910	0.4835
alcohol	1	1	0.4736	0.7979	0.3523	0.5528
alcohol	2	0	0	.	.	.
smoking	0	1	-0.0656	0.5086	0.0166	0.8974
smoking	1	1	-0.6926	0.5927	1.3657	0.2425
smoking	2	0	0	.	.	.
physically_active	0	1	0.8586	0.3902	4.8422	0.0278
physically_active	1	0	0	.	.	.
eGFR_EPI		1	0.00109	0.00786	0.0192	0.8897
lipid_lowering		1	0.5055	0.3887	1.6912	0.1934
DM_duration		1	0.0819	0.0260	9.9212	0.0016

Odds Ratio Estimates

Effect	Point Estimate	95% Wald Confidence Limits	
age	1.055	1.013	1.099
sex 1 vs 2	1.581	0.640	3.905
BMI	1.030	0.938	1.131
hypertension	0.648	0.305	1.374
logLDL_C	0.588	0.217	1.595
HbA1c	1.183	0.953	1.469
alcohol 0 vs 2	1.450	0.513	4.098
alcohol 1 vs 2	1.606	0.336	7.671
smoking 0 vs 2	0.937	0.346	2.538
smoking 1 vs 2	0.500	0.157	1.598
physically_active 0 vs 1	2.360	1.098	5.070
eGFR_EPI	1.001	0.986	1.017
lipid_lowering	1.658	0.774	3.552

Odds Ratio Estimates

Effect	Point Estimate	95% Wald Confidence Limits	
DM_duration	1.085	1.031	1.142

Association of Predicted Probabilities and Observed Responses

Percent Concordant	80.0	Somers' D	0.601
Percent Discordant	19.9	Gamma	0.602
Percent Tied	0.1	Tau-a	0.299
Pairs	11858	c	0.801

The LOGISTIC Procedure

Model Information

**Data Set**	SASUSER.SUN_30DIA1
**Response Variable**	dspn_y
**Number of Response Levels**	2
**Model**	binary logit
**Optimization Technique**	Fisher's scoring

**Number of Observations Read**	219
**Number of Observations Used**	218

Response Profile

Ordered Value	dspn_y	Total Frequency
**1**	0	121
**2**	1	97

Probability modeled is dspn_y=1.

Note: 1 observation was deleted due to missing values for the response or explanatory variables.

Class Level Information

Class	Value	Design Variables		
**sex**	**1**	1	0	
	**2**	0	1	
**alcohol**	**0**	1	0	0
	**1**	0	1	0
	**2**	0	0	1
**smoking**	**0**	1	0	0
	**1**	0	1	0
	**2**	0	0	1
**physically_active**	**0**	1	0	
	**1**	0	1	

Model Convergence Status

Convergence criterion (GCONV=1E-8) satisfied.

Model Fit Statistics

Criterion	Intercept Only	Intercept and Covariates
**AIC**	301.565	253.701
**SC**	304.949	314.622
**-2 Log L**	299.565	217.701

Testing Global Null Hypothesis: BETA=0

Test	Chi-Square	DF	Pr > ChiSq
**Likelihood Ratio**	81.8633	17	<.0001
**Score**	68.9470	17	<.0001
**Wald**	48.2901	17	<.0001

Type 3 Analysis of Effects

Effect	DF	Wald Chi-Square	Pr > ChiSq
**logadi**	1	0.2828	0.5949
**logadi*dr_y**	1	2.0627	0.1509
**dr_y**	1	0.0142	0.9053
**age**	1	4.0272	0.0448
**sex**	1	2.5976	0.1070
**BMI**	1	0.0800	0.7773
**hypertension**	1	0.6846	0.4080
**logLDL_C**	1	0.2531	0.6149
**HbA1c**	1	1.0284	0.3105
**alcohol**	2	0.8598	0.6506
**smoking**	2	0.6944	0.7067
**physically_active**	1	5.4873	0.0192
**eGFR_EPI**	1	0.0120	0.9129
**lipid_lowering**	1	1.9246	0.1653
**DM_duration**	1	5.5325	0.0187

Analysis of Maximum Likelihood Estimates

Parameter	DF	Estimate	Standard Error	Wald Chi-Square	Pr > ChiSq
**Intercept**	1	-7.2923	2.6811	7.3975	0.0065
**logadi**	1	0.1806	0.3396	0.2828	0.5949
**logadi*dr_y**	1	0.9116	0.6347	2.0627	0.1509
**dr_y**	1	-0.1655	1.3901	0.0142	0.9053

Analysis of Maximum Likelihood Estimates

Parameter		DF	Estimate	Standard Error	Wald Chi-Square	Pr > ChiSq
age		1	0.0438	0.0218	4.0272	0.0448
sex	1	1	0.7857	0.4875	2.5976	0.1070
sex	2	0	0	.	.	.
BMI		1	0.0143	0.0506	0.0800	0.7773
hypertension		1	-0.3419	0.4133	0.6846	0.4080
logLDL_C		1	-0.2627	0.5221	0.2531	0.6149
HbA1c		1	0.1159	0.1143	1.0284	0.3105
alcohol	0	1	0.5018	0.5504	0.8313	0.3619
alcohol	1	1	0.4498	0.8950	0.2527	0.6152
alcohol	2	0	0	.	.	.
smoking	0	1	0.2388	0.5258	0.2063	0.6497
smoking	1	1	-0.2555	0.6298	0.1645	0.6850
smoking	2	0	0	.	.	.
physically_active	0	1	0.9805	0.4186	5.4873	0.0192
physically_active	1	0	0	.	.	.
eGFR_EPI		1	0.000854	0.00780	0.0120	0.9129
lipid_lowering		1	0.5528	0.3984	1.9246	0.1653
DM_duration		1	0.0636	0.0270	5.5325	0.0187

Odds Ratio Estimates

Effect	Point Estimate	95% Wald Confidence Limits	
age	1.045	1.001	1.091
sex 1 vs 2	2.194	0.844	5.704
BMI	1.014	0.919	1.120
hypertension	0.710	0.316	1.597
logLDL_C	0.769	0.276	2.140
HbA1c	1.123	0.898	1.405
alcohol 0 vs 2	1.652	0.562	4.857
alcohol 1 vs 2	1.568	0.271	9.061
smoking 0 vs 2	1.270	0.453	3.558
smoking 1 vs 2	0.775	0.225	2.662
physically_active 0 vs 1	2.666	1.174	6.055
eGFR_EPI	1.001	0.986	1.016
lipid_lowering	1.738	0.796	3.795

Odds Ratio Estimates

Effect	Point Estimate	95% Wald Confidence Limits	
**DM_duration**	1.066	1.011	1.124

Association of Predicted Probabilities and Observed Responses

**Percent Concordant**	83.2	**Somers' D**	0.666
**Percent Discordant**	16.6	**Gamma**	0.667
**Percent Tied**	0.2	**Tau-a**	0.330
**Pairs**	11737	**c**	0.833

model3 for logIL6

The LOGISTIC Procedure

Model Information

**Data Set**	SASUSER.SUN_30DIA1
**Response Variable**	dspn_y
**Number of Response Levels**	2
**Model**	binary logit
**Optimization Technique**	Fisher's scoring

**Number of Observations Read**	219
**Number of Observations Used**	218

Response Profile

Ordered Value	dspn_y	Total Frequency
**1**	0	121
**2**	1	97

Probability modeled is dspn_y=1.

Note: 1 observation was deleted due to missing values for the response or explanatory variables.

Class Level Information

Class	Value	Design Variables		
**sex**	**1**	1	0	
	**2**	0	1	
**alcohol**	**0**	1	0	0
	**1**	0	1	0
	**2**	0	0	1
**smoking**	**0**	1	0	0
	**1**	0	1	0
	**2**	0	0	1
**physically_active**	**0**	1	0	
	**1**	0	1	

Model Convergence Status

Convergence criterion (GCONV=1E-8) satisfied.

Model Fit Statistics

Criterion	Intercept Only	Intercept and Covariates
**AIC**	301.565	258.416
**SC**	304.949	319.336
**-2 Log L**	299.565	222.416

Testing Global Null Hypothesis: BETA=0

Test	Chi-Square	DF	Pr > ChiSq
**Likelihood Ratio**	77.1491	17	<.0001
**Score**	67.6898	17	<.0001
**Wald**	50.0562	17	<.0001

Type 3 Analysis of Effects

Effect	DF	Wald Chi-Square	Pr > ChiSq
**logIL6**	1	0.4643	0.4956
**logIL6*dr_y**	1	0.2461	0.6198
**dr_y**	1	6.4542	0.0111
**age**	1	5.8864	0.0153
**sex**	1	1.3427	0.2466
**BMI**	1	0.1091	0.7411
**hypertension**	1	1.4081	0.2354
**logLDL_C**	1	0.3788	0.5382
**HbA1c**	1	0.9946	0.3186
**alcohol**	2	0.3411	0.8432
**smoking**	2	0.8359	0.6584
**physically_active**	1	5.3602	0.0206
**eGFR_EPI**	1	0.1268	0.7218
**lipid_lowering**	1	1.9754	0.1599
**DM_duration**	1	5.6032	0.0179

Analysis of Maximum Likelihood Estimates

Parameter	DF	Estimate	Standard Error	Wald Chi-Square	Pr > ChiSq
**Intercept**	1	-6.4467	2.4574	6.8821	0.0087
**logIL6**	1	-0.0673	0.0987	0.4643	0.4956
**logIL6*dr_y**	1	0.0937	0.1889	0.2461	0.6198
**dr_y**	1	1.5812	0.6224	6.4542	0.0111

Analysis of Maximum Likelihood Estimates

Parameter		DF	Estimate	Standard Error	Wald Chi-Square	Pr > ChiSq
age		1	0.0505	0.0208	5.8864	0.0153
sex	1	1	0.5497	0.4744	1.3427	0.2466
sex	2	0	0	.	.	.
BMI		1	0.0163	0.0495	0.1091	0.7411
hypertension		1	-0.4808	0.4052	1.4081	0.2354
logLDL_C		1	-0.3156	0.5127	0.3788	0.5382
HbA1c		1	0.1129	0.1132	0.9946	0.3186
alcohol	0	1	0.2981	0.5446	0.2996	0.5842
alcohol	1	1	0.3558	0.9140	0.1515	0.6971
alcohol	2	0	0	.	.	.
smoking	0	1	0.2733	0.5217	0.2743	0.6004
smoking	1	1	-0.2579	0.6162	0.1751	0.6756
smoking	2	0	0	.	.	.
physically_active	0	1	0.9585	0.4140	5.3602	0.0206
physically_active	1	0	0	.	.	.
eGFR_EPI		1	-0.00263	0.00740	0.1268	0.7218
lipid_lowering		1	0.5529	0.3934	1.9754	0.1599
DM_duration		1	0.0633	0.0268	5.6032	0.0179

Odds Ratio Estimates

Effect	Point Estimate	95% Wald Confidence Limits	
age	1.052	1.010	1.096
sex 1 vs 2	1.733	0.684	4.391
BMI	1.016	0.923	1.120
hypertension	0.618	0.279	1.368
logLDL_C	0.729	0.267	1.992
HbA1c	1.120	0.897	1.398
alcohol 0 vs 2	1.347	0.463	3.918
alcohol 1 vs 2	1.427	0.238	8.561
smoking 0 vs 2	1.314	0.473	3.654
smoking 1 vs 2	0.773	0.231	2.585
physically_active 0 vs 1	2.608	1.158	5.871
eGFR_EPI	0.997	0.983	1.012
lipid_lowering	1.738	0.804	3.758

Odds Ratio Estimates

Effect	Point Estimate	95% Wald Confidence Limits	
**DM_duration**	1.065	1.011	1.123

Association of Predicted Probabilities and Observed Responses

**Percent Concordant**	82.6	**Somers' D**	0.653
**Percent Discordant**	17.3	**Gamma**	0.654
**Percent Tied**	0.1	**Tau-a**	0.324
**Pairs**	11737	**c**	0.826

model3 for logIL1beta

The LOGISTIC Procedure

Model Information

**Data Set**	SASUSER.SUN_30DIA1
**Response Variable**	dspn_y
**Number of Response Levels**	2
**Model**	binary logit
**Optimization Technique**	Fisher's scoring

**Number of Observations Read**	219
**Number of Observations Used**	218

Response Profile

Ordered Value	dspn_y	Total Frequency
**1**	0	121
**2**	1	97

Probability modeled is dspn_y=1.

Note: 1 observation was deleted due to missing values for the response or explanatory variables.

Class Level Information

Class	Value	Design Variables		
**sex**	**1**	1	0	
	**2**	0	1	
**alcohol**	**0**	1	0	0
	**1**	0	1	0
	**2**	0	0	1
**smoking**	**0**	1	0	0
	**1**	0	1	0
	**2**	0	0	1
**physically_active**	**0**	1	0	
	**1**	0	1	

Model Convergence Status

Convergence criterion (GCONV=1E-8) satisfied.

Model Fit Statistics

Criterion	Intercept Only	Intercept and Covariates
AIC	301.565	254.674
SC	304.949	315.595
-2 Log L	299.565	218.674

Testing Global Null Hypothesis: BETA=0

Test	Chi-Square	DF	Pr > ChiSq
Likelihood Ratio	80.8908	17	<.0001
Score	69.6978	17	<.0001
Wald	50.3928	17	<.0001

Type 3 Analysis of Effects

Effect	DF	Wald Chi-Square	Pr > ChiSq
logIL1beta	1	3.2623	0.0709
logIL1beta*dr_y	1	0.4773	0.4896
dr_y	1	14.3171	0.0002
age	1	5.4048	0.0201
sex	1	1.1103	0.2920
BMI	1	0.1243	0.7244
hypertension	1	1.2629	0.2611
logLDL_C	1	0.3277	0.5670
HbA1c	1	0.6103	0.4347
alcohol	2	0.5913	0.7441
smoking	2	0.8424	0.6563
physically_active	1	5.9615	0.0146
eGFR_EPI	1	0.0452	0.8317
lipid_lowering	1	1.8548	0.1732
DM_duration	1	5.9246	0.0149

Analysis of Maximum Likelihood Estimates

Parameter	DF	Estimate	Standard Error	Wald Chi-Square	Pr > ChiSq
Intercept	1	-6.3700	2.5011	6.4867	0.0109
logIL1beta	1	-0.3241	0.1794	3.2623	0.0709
logIL1beta*dr_y	1	0.2073	0.3001	0.4773	0.4896
dr_y	1	1.6778	0.4434	14.3171	0.0002

Analysis of Maximum Likelihood Estimates

Parameter		DF	Estimate	Standard Error	Wald Chi-Square	Pr > ChiSq
age		1	0.0494	0.0213	5.4048	0.0201
sex	1	1	0.5075	0.4817	1.1103	0.2920
sex	2	0	0	.	.	.
BMI		1	0.0178	0.0506	0.1243	0.7244
hypertension		1	-0.4563	0.4060	1.2629	0.2611
logLDL_C		1	-0.2973	0.5194	0.3277	0.5670
HbA1c		1	0.0895	0.1145	0.6103	0.4347
alcohol	0	1	0.3318	0.5445	0.3713	0.5423
alcohol	1	1	0.6291	0.9614	0.4282	0.5129
alcohol	2	0	0	.	.	.
smoking	0	1	0.2278	0.5275	0.1864	0.6659
smoking	1	1	-0.3218	0.6249	0.2652	0.6066
smoking	2	0	0	.	.	.
physically_active	0	1	1.0130	0.4149	5.9615	0.0146
physically_active	1	0	0	.	.	.
eGFR_EPI		1	-0.00160	0.00754	0.0452	0.8317
lipid_lowering		1	0.5429	0.3986	1.8548	0.1732
DM_duration		1	0.0657	0.0270	5.9246	0.0149

Odds Ratio Estimates

Effect	Point Estimate	95% Wald Confidence Limits	
age	1.051	1.008	1.095
sex 1 vs 2	1.661	0.646	4.270
BMI	1.018	0.922	1.124
hypertension	0.634	0.286	1.404
logLDL_C	0.743	0.268	2.056
HbA1c	1.094	0.874	1.369
alcohol 0 vs 2	1.393	0.479	4.051
alcohol 1 vs 2	1.876	0.285	12.348
smoking 0 vs 2	1.256	0.447	3.531
smoking 1 vs 2	0.725	0.213	2.467
physically_active 0 vs 1	2.754	1.221	6.210
eGFR_EPI	0.998	0.984	1.013
lipid_lowering	1.721	0.788	3.759

Odds Ratio Estimates

Effect	Point Estimate	95% Wald Confidence Limits	
**DM_duration**	1.068	1.013	1.126

Association of Predicted Probabilities and Observed Responses

**Percent Concordant**	83.4	**Somers' D**	0.669
**Percent Discordant**	16.5	**Gamma**	0.670
**Percent Tied**	0.2	**Tau-a**	0.332
**Pairs**	11737	**c**	0.835

model3 for leptin

The LOGISTIC Procedure

Model Information

**Data Set**	SASUSER.SUN_30DIA1
**Response Variable**	dspn_y
**Number of Response Levels**	2
**Model**	binary logit
**Optimization Technique**	Fisher's scoring

**Number of Observations Read**	219
**Number of Observations Used**	218

Response Profile

Ordered Value	dspn_y	Total Frequency
**1**	0	121
**2**	1	97

Probability modeled is dspn_y=1.

Note: 1 observation was deleted due to missing values for the response or explanatory variables.

Class Level Information

Class	Value	Design Variables		
**sex**	**1**	1	0	
	**2**	0	1	
**alcohol**	**0**	1	0	0
	**1**	0	1	0
	**2**	0	0	1
**smoking**	**0**	1	0	0
	**1**	0	1	0
	**2**	0	0	1
**physically_active**	**0**	1	0	
	**1**	0	1	

Model Convergence Status

Convergence criterion (GCONV=1E-8) satisfied.

Model Fit Statistics

Criterion	Intercept Only	Intercept and Covariates
**AIC**	301.565	257.125
**SC**	304.949	318.046
**-2 Log L**	299.565	221.125

Testing Global Null Hypothesis: BETA=0

Test	Chi-Square	DF	Pr > ChiSq
**Likelihood Ratio**	78.4393	17	<.0001
**Score**	68.4402	17	<.0001
**Wald**	49.7403	17	<.0001

Type 3 Analysis of Effects

Effect	DF	Wald Chi-Square	Pr > ChiSq
**Leptin**	1	0.4005	0.5268
**Leptin*dr_y**	1	0.4244	0.5148
**dr_y**	1	8.3397	0.0039
**age**	1	5.0818	0.0242
**sex**	1	2.6511	0.1035
**BMI**	1	0.0615	0.8042
**hypertension**	1	1.4043	0.2360
**logLDL_C**	1	0.1412	0.7071
**HbA1c**	1	1.0993	0.2944
**alcohol**	2	0.2692	0.8741
**smoking**	2	1.2982	0.5225
**physically_active**	1	4.7353	0.0295
**eGFR_EPI**	1	0.0178	0.8937
**lipid_lowering**	1	2.0448	0.1527
**DM_duration**	1	5.2735	0.0217

Analysis of Maximum Likelihood Estimates

Parameter	DF	Estimate	Standard Error	Wald Chi-Square	Pr > ChiSq
**Intercept**	1	-6.2269	2.4785	6.3119	0.0120
**Leptin**	1	0.0137	0.0217	0.4005	0.5268
**Leptin*dr_y**	1	0.0204	0.0313	0.4244	0.5148
**dr_y**	1	1.5645	0.5418	8.3397	0.0039

Analysis of Maximum Likelihood Estimates

Parameter		DF	Estimate	Standard Error	Wald Chi-Square	Pr > ChiSq
age		1	0.0473	0.0210	5.0818	0.0242
sex	1	1	0.8540	0.5245	2.6511	0.1035
sex	2	0	0	.	.	.
BMI		1	-0.0139	0.0561	0.0615	0.8042
hypertension		1	-0.4797	0.4048	1.4043	0.2360
logLDL_C		1	-0.1953	0.5198	0.1412	0.7071
HbA1c		1	0.1178	0.1124	1.0993	0.2944
alcohol	0	1	0.2697	0.5399	0.2495	0.6174
alcohol	1	1	0.2844	0.8970	0.1005	0.7512
alcohol	2	0	0	.	.	.
smoking	0	1	0.3883	0.5359	0.5250	0.4687
smoking	1	1	-0.2658	0.6135	0.1878	0.6648
smoking	2	0	0	.	.	.
physically_active	0	1	0.8990	0.4131	4.7353	0.0295
physically_active	1	0	0	.	.	.
eGFR_EPI		1	-0.00100	0.00749	0.0178	0.8937
lipid_lowering		1	0.5777	0.4040	2.0448	0.1527
DM_duration		1	0.0627	0.0273	5.2735	0.0217

Odds Ratio Estimates

Effect	Point Estimate	95% Wald Confidence Limits	
age	1.048	1.006	1.093
sex 1 vs 2	2.349	0.840	6.567
BMI	0.986	0.884	1.101
hypertension	0.619	0.280	1.369
logLDL_C	0.823	0.297	2.278
HbA1c	1.125	0.903	1.402
alcohol 0 vs 2	1.310	0.455	3.773
alcohol 1 vs 2	1.329	0.229	7.710
smoking 0 vs 2	1.474	0.516	4.215
smoking 1 vs 2	0.767	0.230	2.551
physically_active 0 vs 1	2.457	1.093	5.522
eGFR_EPI	0.999	0.984	1.014
lipid_lowering	1.782	0.807	3.933

Odds Ratio Estimates

Effect	Point Estimate	95% Wald Confidence Limits	
**DM_duration**	1.065	1.009	1.123

Association of Predicted Probabilities and Observed Responses

**Percent Concordant**	82.4	**Somers' D**	0.649
**Percent Discordant**	17.4	**Gamma**	0.651
**Percent Tied**	0.2	**Tau-a**	0.322
**Pairs**	11737	**c**	0.825

model3 for logtnfa

The LOGISTIC Procedure

Model Information

**Data Set**	SASUSER.SUN_30DIA1
**Response Variable**	dspn_y
**Number of Response Levels**	2
**Model**	binary logit
**Optimization Technique**	Fisher's scoring

**Number of Observations Read**	219
**Number of Observations Used**	218

Response Profile

Ordered Value	dspn_y	Total Frequency
**1**	0	121
**2**	1	97

Probability modeled is dspn_y=1.

Note: 1 observation was deleted due to missing values for the response or explanatory variables.

Class Level Information

Class	Value	Design Variables		
**sex**	**1**	1	0	
	**2**	0	1	
**alcohol**	**0**	1	0	0
	**1**	0	1	0
	**2**	0	0	1
**smoking**	**0**	1	0	0
	**1**	0	1	0
	**2**	0	0	1
**physically_active**	**0**	1	0	
	**1**	0	1	

Model Convergence Status

Convergence criterion (GCONV=1E-8) satisfied.

Model Fit Statistics

Criterion	Intercept Only	Intercept and Covariates
AIC	301.565	256.885
SC	304.949	317.806
-2 Log L	299.565	220.885

Testing Global Null Hypothesis: BETA=0

Test	Chi-Square	DF	Pr > ChiSq
Likelihood Ratio	78.6796	17	<.0001
Score	68.6213	17	<.0001
Wald	50.0822	17	<.0001

Type 3 Analysis of Effects

Effect	DF	Wald Chi-Square	Pr > ChiSq
logtnfa	1	1.5110	0.2190
logtnfa*dr_y	1	0.0151	0.9022
dr_y	1	2.8126	0.0935
age	1	5.4288	0.0198
sex	1	1.5494	0.2132
BMI	1	0.1783	0.6729
hypertension	1	1.4907	0.2221
logLDL_C	1	0.3927	0.5309
HbA1c	1	0.9722	0.3241
alcohol	2	0.4308	0.8062
smoking	2	0.9637	0.6177
physically_active	1	5.4594	0.0195
eGFR_EPI	1	0.0687	0.7933
lipid_lowering	1	2.2444	0.1341
DM_duration	1	6.4155	0.0113

Analysis of Maximum Likelihood Estimates

Parameter	DF	Estimate	Standard Error	Wald Chi-Square	Pr > ChiSq
Intercept	1	-6.1772	2.4716	6.2464	0.0124
logtnfa	1	-0.2422	0.1970	1.5110	0.2190
logtnfa*dr_y	1	0.0440	0.3583	0.0151	0.9022
dr_y	1	1.7093	1.0192	2.8126	0.0935

Analysis of Maximum Likelihood Estimates

Parameter		DF	Estimate	Standard Error	Wald Chi-Square	Pr > ChiSq
age		1	0.0490	0.0210	5.4288	0.0198
sex	1	1	0.5953	0.4783	1.5494	0.2132
sex	2	0	0	.	.	.
BMI		1	0.0209	0.0496	0.1783	0.6729
hypertension		1	-0.4942	0.4048	1.4907	0.2221
logLDL_C		1	-0.3221	0.5139	0.3927	0.5309
HbA1c		1	0.1125	0.1141	0.9722	0.3241
alcohol	0	1	0.3372	0.5402	0.3896	0.5325
alcohol	1	1	0.3843	0.9256	0.1724	0.6780
alcohol	2	0	0	.	.	.
smoking	0	1	0.2385	0.5241	0.2071	0.6490
smoking	1	1	-0.3436	0.6196	0.3075	0.5792
smoking	2	0	0	.	.	.
physically_active	0	1	0.9680	0.4143	5.4594	0.0195
physically_active	1	0	0	.	.	.
eGFR_EPI		1	-0.00195	0.00743	0.0687	0.7933
lipid_lowering		1	0.5949	0.3971	2.2444	0.1341
DM_duration		1	0.0685	0.0271	6.4155	0.0113

Odds Ratio Estimates

Effect	Point Estimate	95% Wald Confidence Limits	
age	1.050	1.008	1.094
sex 1 vs 2	1.814	0.710	4.631
BMI	1.021	0.927	1.125
hypertension	0.610	0.276	1.349
logLDL_C	0.725	0.265	1.984
HbA1c	1.119	0.895	1.399
alcohol 0 vs 2	1.401	0.486	4.038
alcohol 1 vs 2	1.469	0.239	9.012
smoking 0 vs 2	1.269	0.454	3.546
smoking 1 vs 2	0.709	0.211	2.389
physically_active 0 vs 1	2.633	1.169	5.930
eGFR_EPI	0.998	0.984	1.013
lipid_lowering	1.813	0.832	3.948

Odds Ratio Estimates

Effect	Point Estimate	95% Wald Confidence Limits	
**DM_duration**	1.071	1.016	1.129

Association of Predicted Probabilities and Observed Responses

**Percent Concordant**	82.8	**Somers' D**	0.657
**Percent Discordant**	17.1	**Gamma**	0.657
**Percent Tied**	0.1	**Tau-a**	0.326
**Pairs**	11737	**c**	0.828

model3 for loghsCRP

The LOGISTIC Procedure

Model Information

**Data Set**	SASUSER.SUN_30DIA1
**Response Variable**	dspn_y
**Number of Response Levels**	2
**Model**	binary logit
**Optimization Technique**	Fisher's scoring

**Number of Observations Read**	219
**Number of Observations Used**	218

Response Profile

Ordered Value	dspn_y	Total Frequency
**1**	0	121
**2**	1	97

Probability modeled is dspn_y=1.

Note: 1 observation was deleted due to missing values for the response or explanatory variables.

Class Level Information

Class	Value	Design Variables		
**sex**	**1**	1	0	
	**2**	0	1	
**alcohol**	**0**	1	0	0
	**1**	0	1	0
	**2**	0	0	1
**smoking**	**0**	1	0	0
	**1**	0	1	0
	**2**	0	0	1
**physically_active**	**0**	1	0	
	**1**	0	1	

Model Convergence Status

Convergence criterion (GCONV=1E-8) satisfied.

Model Fit Statistics

Criterion	Intercept Only	Intercept and Covariates
AIC	301.565	255.803
SC	304.949	316.724
-2 Log L	299.565	219.803

Testing Global Null Hypothesis: BETA=0

Test	Chi-Square	DF	Pr > ChiSq
Likelihood Ratio	79.7616	17	<.0001
Score	69.5396	17	<.0001
Wald	50.3858	17	<.0001

Type 3 Analysis of Effects

Effect	DF	Wald Chi-Square	Pr > ChiSq
loghsCRP	1	1.7075	0.1913
loghsCRP*dr_y	1	0.0853	0.7703
dr_y	1	16.1606	<.0001
age	1	5.0684	0.0244
sex	1	1.1994	0.2735
BMI	1	0.4741	0.4911
hypertension	1	0.8403	0.3593
logLDL_C	1	0.2923	0.5888
HbA1c	1	2.1226	0.1451
alcohol	2	0.6635	0.7177
smoking	2	1.1227	0.5704
physically_active	1	5.9487	0.0147
eGFR_EPI	1	0.6847	0.4080
lipid_lowering	1	1.5974	0.2063
DM_duration	1	6.0517	0.0139

Analysis of Maximum Likelihood Estimates

Parameter	DF	Estimate	Standard Error	Wald Chi-Square	Pr > ChiSq
Intercept	1	-7.2029	2.4651	8.5381	0.0035
loghsCRP	1	-0.3071	0.2350	1.7075	0.1913
loghsCRP*dr_y	1	-0.1058	0.3624	0.0853	0.7703
dr_y	1	1.6960	0.4219	16.1606	<.0001

Analysis of Maximum Likelihood Estimates

Parameter		DF	Estimate	Standard Error	Wald Chi-Square	Pr > ChiSq
**age**		1	0.0470	0.0209	5.0684	0.0244
**sex**	**1**	1	0.5197	0.4746	1.1994	0.2735
**sex**	**2**	0	0	.	.	.
**BMI**		1	0.0350	0.0509	0.4741	0.4911
**hypertension**		1	-0.3824	0.4171	0.8403	0.3593
**logLDL_C**		1	-0.2764	0.5112	0.2923	0.5888
**HbA1c**		1	0.1756	0.1205	2.1226	0.1451
**alcohol**	**0**	1	0.3869	0.5497	0.4955	0.4815
**alcohol**	**1**	1	0.6008	0.9292	0.4180	0.5179
**alcohol**	**2**	0	0	.	.	.
**smoking**	**0**	1	0.2312	0.5246	0.1943	0.6594
**smoking**	**1**	1	-0.3900	0.6248	0.3897	0.5325
**smoking**	**2**	0	0	.	.	.
**physically_active**	**0**	1	1.0592	0.4343	5.9487	0.0147
**physically_active**	**1**	0	0	.	.	.
**eGFR_EPI**		1	-0.00652	0.00788	0.6847	0.4080
**lipid_lowering**		1	0.5076	0.4016	1.5974	0.2063
**DM_duration**		1	0.0668	0.0272	6.0517	0.0139

Odds Ratio Estimates

Effect	Point Estimate	95% Wald Confidence Limits	
**age**	1.048	1.006	1.092
**sex 1 vs 2**	1.682	0.663	4.262
**BMI**	1.036	0.937	1.144
**hypertension**	0.682	0.301	1.545
**logLDL_C**	0.759	0.278	2.066
**HbA1c**	1.192	0.941	1.510
**alcohol 0 vs 2**	1.472	0.501	4.324
**alcohol 1 vs 2**	1.824	0.295	11.269
**smoking 0 vs 2**	1.260	0.451	3.523
**smoking 1 vs 2**	0.677	0.199	2.304
**physically_active 0 vs 1**	2.884	1.231	6.756
**eGFR_EPI**	0.994	0.978	1.009
**lipid_lowering**	1.661	0.756	3.650

Odds Ratio Estimates

Effect	Point Estimate	95% Wald Confidence Limits	
**DM_duration**	1.069	1.014	1.128

Association of Predicted Probabilities and Observed Responses

**Percent Concordant**	83.0	**Somers' D**	0.662
**Percent Discordant**	16.9	**Gamma**	0.662
**Percent Tied**	0.1	**Tau-a**	0.328
**Pairs**	11737	**c**	0.831

model3 for logl2n

The LOGISTIC Procedure

Model Information

**Data Set**	SASUSER.SUN_30DIA1
**Response Variable**	dspn_y
**Number of Response Levels**	2
**Model**	binary logit
**Optimization Technique**	Fisher's scoring

**Number of Observations Read**	219
**Number of Observations Used**	218

Response Profile

Ordered Value	dspn_y	Total Frequency
**1**	0	121
**2**	1	97

Probability modeled is dspn_y=1.

Note: 1 observation was deleted due to missing values for the response or explanatory variables.

Class Level Information

Class	Value	Design Variables		
**sex**	**1**	1	0	
	**2**	0	1	
**alcohol**	**0**	1	0	0
	**1**	0	1	0
	**2**	0	0	1
**smoking**	**0**	1	0	0
	**1**	0	1	0
	**2**	0	0	1
**physically_active**	**0**	1	0	
	**1**	0	1	

Model Convergence Status

Convergence criterion (GCONV=1E-8) satisfied.

Model Fit Statistics

Criterion	Intercept Only	Intercept and Covariates
**AIC**	301.565	257.376
**SC**	304.949	318.297
**-2 Log L**	299.565	221.376

Testing Global Null Hypothesis: BETA=0

Test	Chi-Square	DF	Pr > ChiSq
**Likelihood Ratio**	78.1887	17	<.0001
**Score**	68.2733	17	<.0001
**Wald**	50.1766	17	<.0001

Type 3 Analysis of Effects

Effect	DF	Wald Chi-Square	Pr > ChiSq
**logl2n**	1	1.3273	0.2493
**logl2n*dr_y**	1	0.8768	0.3491
**dr_y**	1	0.0901	0.7641
**age**	1	6.0058	0.0143
**sex**	1	1.0907	0.2963
**BMI**	1	0.1211	0.7278
**hypertension**	1	1.9284	0.1649
**logLDL_C**	1	0.5577	0.4552
**HbA1c**	1	1.2126	0.2708
**alcohol**	2	0.2142	0.8984
**smoking**	2	0.6440	0.7247
**physically_active**	1	5.0817	0.0242
**eGFR_EPI**	1	0.1011	0.7505
**lipid_lowering**	1	1.8783	0.1705
**DM_duration**	1	5.6104	0.0179

Analysis of Maximum Likelihood Estimates

Parameter	DF	Estimate	Standard Error	Wald Chi-Square	Pr > ChiSq
**Intercept**	1	-5.0076	2.9351	2.9108	0.0880
**logl2n**	1	-0.3500	0.3038	1.3273	0.2493
**logl2n*dr_y**	1	0.5667	0.6052	0.8768	0.3491
**dr_y**	1	-0.8713	2.9033	0.0901	0.7641

Analysis of Maximum Likelihood Estimates

Parameter		DF	Estimate	Standard Error	Wald Chi-Square	Pr > ChiSq
**age**		1	0.0524	0.0214	6.0058	0.0143
**sex**	**1**	1	0.5015	0.4802	1.0907	0.2963
**sex**	**2**	0	0	.	.	.
**BMI**		1	0.0173	0.0496	0.1211	0.7278
**hypertension**		1	-0.5621	0.4048	1.9284	0.1649
**logLDL_C**		1	-0.3841	0.5144	0.5577	0.4552
**HbA1c**		1	0.1263	0.1147	1.2126	0.2708
**alcohol**	**0**	1	0.2459	0.5542	0.1969	0.6572
**alcohol**	**1**	1	0.2638	0.9170	0.0828	0.7736
**alcohol**	**2**	0	0	.	.	.
**smoking**	**0**	1	0.2235	0.5280	0.1792	0.6721
**smoking**	**1**	1	-0.2483	0.6160	0.1624	0.6869
**smoking**	**2**	0	0	.	.	.
**physically_active**	**0**	1	0.9378	0.4160	5.0817	0.0242
**physically_active**	**1**	0	0	.	.	.
**eGFR_EPI**		1	-0.00243	0.00765	0.1011	0.7505
**lipid_lowering**		1	0.5407	0.3945	1.8783	0.1705
**DM_duration**		1	0.0638	0.0269	5.6104	0.0179

Odds Ratio Estimates

Effect	Point Estimate	95% Wald Confidence Limits	
**age**	1.054	1.011	1.099
**sex 1 vs 2**	1.651	0.644	4.232
**BMI**	1.017	0.923	1.121
**hypertension**	0.570	0.258	1.260
**logLDL_C**	0.681	0.249	1.866
**HbA1c**	1.135	0.906	1.420
**alcohol 0 vs 2**	1.279	0.432	3.789
**alcohol 1 vs 2**	1.302	0.216	7.855
**smoking 0 vs 2**	1.250	0.444	3.520
**smoking 1 vs 2**	0.780	0.233	2.609
**physically_active 0 vs 1**	2.554	1.130	5.773
**eGFR_EPI**	0.998	0.983	1.013
**lipid_lowering**	1.717	0.793	3.720

Odds Ratio Estimates

Effect	Point Estimate	95% Wald Confidence Limits	
DM_duration	1.066	1.011	1.124

Association of Predicted Probabilities and Observed Responses

Percent Concordant	82.5	Somers' D	0.652
Percent Discordant	17.3	Gamma	0.654
Percent Tied	0.2	Tau-a	0.324
Pairs	11737	c	0.826

The LOGISTIC Procedure

Model Information

**Data Set**	SASUSER.SUN_30DIA1
**Response Variable**	dspn_y
**Number of Response Levels**	2
**Model**	binary logit
**Optimization Technique**	Fisher's scoring

**Number of Observations Read**	219
**Number of Observations Used**	219

Response Profile

Ordered Value	dspn_y	Total Frequency
**1**	0	121
**2**	1	98

Probability modeled is dspn_y=1.

Class Level Information

Class	Value	Design Variables		
**lipid_lowering**	**0**	1	0	
	**1**	0	1	
**sex**	**1**	1	0	
	**2**	0	1	
**alcohol**	**0**	1	0	0
	**1**	0	1	0
	**2**	0	0	1
**smoking**	**0**	1	0	0
	**1**	0	1	0
	**2**	0	0	1
**physically_active**	**0**	1	0	
	**1**	0	1	

Model Convergence Status

Convergence criterion (GCONV=1E-8) satisfied.

Model Fit Statistics

Criterion	Intercept Only	Intercept and Covariates
AIC	303.178	275.302
SC	306.568	332.917
-2 Log L	301.178	241.302

Testing Global Null Hypothesis: BETA=0

Test	Chi-Square	DF	Pr > ChiSq
Likelihood Ratio	59.8762	16	<.0001
Score	52.4078	16	<.0001
Wald	39.7858	16	0.0008

Type 3 Analysis of Effects

Effect	DF	Wald Chi-Square	Pr > ChiSq
logadi	1	3.9126	0.0479
logadi*lipid_lowerin	1	0.0003	0.9854
lipid_lowering	1	0.2242	0.6358
age	1	2.7726	0.0959
sex	1	2.4786	0.1154
BMI	1	0.3153	0.5745
hypertension	1	0.6547	0.4184
logLDL_C	1	1.4177	0.2338
HbA1c	1	4.7366	0.0295
alcohol	2	1.9728	0.3729
smoking	2	2.0215	0.3640
physically_active	1	6.3185	0.0119
eGFR_EPI	1	0.5941	0.4408
DM_duration	1	11.9825	0.0005

Analysis of Maximum Likelihood Estimates

Parameter		DF	Estimate	Standard Error	Wald Chi-Square	Pr > ChiSq
Intercept		1	-6.9249	2.4479	8.0031	0.0047
logadi		1	0.5478	0.4186	1.7124	0.1907
logadi*lipid_lowerin	0	1	-0.00926	0.5046	0.0003	0.9854
logadi*lipid_lowerin	1	0	0	.	.	.
lipid_lowering	0	1	-0.5432	1.1472	0.2242	0.6358

Analysis of Maximum Likelihood Estimates

Parameter		DF	Estimate	Standard Error	Wald Chi-Square	Pr > ChiSq
lipid_lowering	1	0	0	.	.	.
age		1	0.0325	0.0195	2.7726	0.0959
sex	1	1	0.7296	0.4634	2.4786	0.1154
sex	2	0	0	.	.	.
BMI		1	0.0264	0.0471	0.3153	0.5745
hypertension		1	-0.3117	0.3852	0.6547	0.4184
logLDL_C		1	-0.5889	0.4946	1.4177	0.2338
HbA1c		1	0.2247	0.1032	4.7366	0.0295
alcohol	0	1	0.6738	0.5164	1.7026	0.1919
alcohol	1	1	0.7714	0.8037	0.9212	0.3372
alcohol	2	0	0	.	.	.
smoking	0	1	0.0969	0.4993	0.0377	0.8461
smoking	1	1	-0.6932	0.5947	1.3586	0.2438
smoking	2	0	0	.	.	.
physically_active	0	1	0.9669	0.3847	6.3185	0.0119
physically_active	1	0	0	.	.	.
eGFR_EPI		1	-0.00548	0.00711	0.5941	0.4408
DM_duration		1	0.0882	0.0255	11.9825	0.0005

Odds Ratio Estimates

Effect	Point Estimate	95% Wald Confidence Limits	
age	1.033	0.994	1.073
sex 1 vs 2	2.074	0.836	5.144
BMI	1.027	0.936	1.126
hypertension	0.732	0.344	1.558
logLDL_C	0.555	0.210	1.463
HbA1c	1.252	1.023	1.533
alcohol 0 vs 2	1.962	0.713	5.397
alcohol 1 vs 2	2.163	0.448	10.451
smoking 0 vs 2	1.102	0.414	2.932
smoking 1 vs 2	0.500	0.156	1.604
physically_active 0 vs 1	2.630	1.237	5.589
eGFR_EPI	0.995	0.981	1.008
DM_duration	1.092	1.039	1.148

Association of Predicted Probabilities and Observed Responses

**Percent Concordant**	78.4	**Somers' D**	0.571
**Percent Discordant**	21.4	**Gamma**	0.572
**Percent Tied**	0.2	**Tau-a**	0.283
**Pairs**	11858	**c**	0.785

The LOGISTIC Procedure

Model Information

**Data Set**	SASUSER.SUN_30DIA1
**Response Variable**	dspn_y
**Number of Response Levels**	2
**Model**	binary logit
**Optimization Technique**	Fisher's scoring

**Number of Observations Read**	219
**Number of Observations Used**	219

Response Profile

Ordered Value	dspn_y	Total Frequency
**1**	0	121
**2**	1	98

Probability modeled is dspn_y=1.

Class Level Information

Class	Value	Design Variables		
**lipid_lowering**	**0**	1	0	
	**1**	0	1	
**sex**	**1**	1	0	
	**2**	0	1	
**alcohol**	**0**	1	0	0
	**1**	0	1	0
	**2**	0	0	1
**smoking**	**0**	1	0	0
	**1**	0	1	0
	**2**	0	0	1
**physically_active**	**0**	1	0	
	**1**	0	1	

Model Convergence Status

Convergence criterion (GCONV=1E-8) satisfied.

Model Fit Statistics

Criterion	Intercept Only	Intercept and Covariates
**AIC**	303.178	279.400
**SC**	306.568	337.014
**-2 Log L**	301.178	245.400

Testing Global Null Hypothesis: BETA=0

Test	Chi-Square	DF	Pr > ChiSq
**Likelihood Ratio**	55.7783	16	<.0001
**Score**	50.0199	16	<.0001
**Wald**	39.4232	16	0.0009

Type 3 Analysis of Effects

Effect	DF	Wald Chi-Square	Pr > ChiSq
**logIL6**	1	0.1176	0.7316
**logIL6*lipid_lowerin**	1	0.4604	0.4974
**lipid_lowering**	1	1.9722	0.1602
**age**	1	4.1040	0.0428
**sex**	1	1.3142	0.2516
**BMI**	1	0.5442	0.4607
**hypertension**	1	1.3540	0.2446
**logLDL_C**	1	1.1946	0.2744
**HbA1c**	1	4.2357	0.0396
**alcohol**	2	1.4882	0.4752
**smoking**	2	1.5718	0.4557
**physically_active**	1	5.9339	0.0149
**eGFR_EPI**	1	1.5507	0.2130
**DM_duration**	1	12.3366	0.0004

Analysis of Maximum Likelihood Estimates

Parameter		DF	Estimate	Standard Error	Wald Chi-Square	Pr > ChiSq
**Intercept**		1	-5.5459	2.2966	5.8313	0.0157
**logIL6**		1	-0.0827	0.1259	0.4318	0.5111
**logIL6*lipid_lowerin**	**0**	1	0.1104	0.1627	0.4604	0.4974
**logIL6*lipid_lowerin**	**1**	0	0	.	.	.
**lipid_lowering**	**0**	1	-0.8016	0.5708	1.9722	0.1602

Analysis of Maximum Likelihood Estimates

Parameter		DF	Estimate	Standard Error	Wald Chi-Square	Pr > ChiSq
lipid_lowering	1	0	0	.	.	.
age		1	0.0385	0.0190	4.1040	0.0428
sex	1	1	0.5115	0.4462	1.3142	0.2516
sex	2	0	0	.	.	.
BMI		1	0.0341	0.0462	0.5442	0.4607
hypertension		1	-0.4408	0.3788	1.3540	0.2446
logLDL_C		1	-0.5370	0.4913	1.1946	0.2744
HbA1c		1	0.2112	0.1026	4.2357	0.0396
alcohol	0	1	0.5643	0.5173	1.1896	0.2754
alcohol	1	1	0.7214	0.7981	0.8170	0.3661
alcohol	2	0	0	.	.	.
smoking	0	1	0.1802	0.4939	0.1332	0.7152
smoking	1	1	-0.5289	0.5835	0.8216	0.3647
smoking	2	0	0	.	.	.
physically_active	0	1	0.9298	0.3817	5.9339	0.0149
physically_active	1	0	0	.	.	.
eGFR_EPI		1	-0.00851	0.00684	1.5507	0.2130
DM_duration		1	0.0891	0.0254	12.3366	0.0004

Odds Ratio Estimates

Effect	Point Estimate	95% Wald Confidence Limits	
age	1.039	1.001	1.079
sex 1 vs 2	1.668	0.696	3.999
BMI	1.035	0.945	1.133
hypertension	0.644	0.306	1.352
logLDL_C	0.584	0.223	1.531
HbA1c	1.235	1.010	1.510
alcohol 0 vs 2	1.758	0.638	4.846
alcohol 1 vs 2	2.057	0.430	9.833
smoking 0 vs 2	1.197	0.455	3.152
smoking 1 vs 2	0.589	0.188	1.849
physically_active 0 vs 1	2.534	1.199	5.355
eGFR_EPI	0.992	0.978	1.005
DM_duration	1.093	1.040	1.149

Association of Predicted Probabilities and Observed Responses

**Percent Concordant**	77.6	**Somers' D**	0.554
**Percent Discordant**	22.2	**Gamma**	0.555
**Percent Tied**	0.2	**Tau-a**	0.275
**Pairs**	11858	**c**	0.777

model3 for logIL1beta

The LOGISTIC Procedure

Model Information

**Data Set**	SASUSER.SUN_30DIA1
**Response Variable**	dspn_y
**Number of Response Levels**	2
**Model**	binary logit
**Optimization Technique**	Fisher's scoring

**Number of Observations Read**	219
**Number of Observations Used**	219

Response Profile

Ordered Value	dspn_y	Total Frequency
**1**	0	121
**2**	1	98

Probability modeled is dspn_y=1.

Class Level Information

Class	Value	Design Variables		
**lipid_lowering**	**0**	1	0	
	**1**	0	1	
**sex**	**1**	1	0	
	**2**	0	1	
**alcohol**	**0**	1	0	0
	**1**	0	1	0
	**2**	0	0	1
**smoking**	**0**	1	0	0
	**1**	0	1	0
	**2**	0	0	1
**physically_active**	**0**	1	0	
	**1**	0	1	

Model Convergence Status

Convergence criterion (GCONV=1E-8) satisfied.

Model Fit Statistics

Criterion	Intercept Only	Intercept and Covariates
AIC	303.178	275.853
SC	306.568	333.468
-2 Log L	301.178	241.853

Testing Global Null Hypothesis: BETA=0

Test	Chi-Square	DF	Pr > ChiSq
Likelihood Ratio	59.3251	16	<.0001
Score	51.9386	16	<.0001
Wald	40.2009	16	0.0007

Type 3 Analysis of Effects

Effect	DF	Wald Chi-Square	Pr > ChiSq
logIL1beta	1	3.4370	0.0638
logIL1bet*lipid_lowe	1	0.0118	0.9135
lipid_lowering	1	1.4309	0.2316
age	1	3.6709	0.0554
sex	1	1.0653	0.3020
BMI	1	0.5225	0.4698
hypertension	1	1.1346	0.2868
logLDL_C	1	1.0167	0.3133
HbA1c	1	3.6667	0.0555
alcohol	2	1.7661	0.4135
smoking	2	1.6688	0.4341
physically_active	1	6.6843	0.0097
eGFR_EPI	1	1.0437	0.3070
DM_duration	1	12.9706	0.0003

Analysis of Maximum Likelihood Estimates

Parameter		DF	Estimate	Standard Error	Wald Chi-Square	Pr > ChiSq
Intercept		1	-5.5486	2.3046	5.7968	0.0161
logIL1beta		1	-0.2327	0.2009	1.3421	0.2467
logIL1bet*lipid_lowe	0	1	-0.0285	0.2624	0.0118	0.9135
logIL1bet*lipid_lowe	1	0	0	.	.	.
lipid_lowering	0	1	-0.5018	0.4195	1.4309	0.2316

Analysis of Maximum Likelihood Estimates

Parameter		DF	Estimate	Standard Error	Wald Chi-Square	Pr > ChiSq
lipid_lowering	1	0	0	.	.	.
age		1	0.0367	0.0192	3.6709	0.0554
sex	1	1	0.4656	0.4511	1.0653	0.3020
sex	2	0	0	.	.	.
BMI		1	0.0337	0.0466	0.5225	0.4698
hypertension		1	-0.4049	0.3801	1.1346	0.2868
logLDL_C		1	-0.5050	0.5009	1.0167	0.3133
HbA1c		1	0.1984	0.1036	3.6667	0.0555
alcohol	0	1	0.5005	0.5111	0.9592	0.3274
alcohol	1	1	1.0003	0.8488	1.3887	0.2386
alcohol	2	0	0	.	.	.
smoking	0	1	0.1022	0.4944	0.0427	0.8363
smoking	1	1	-0.6162	0.5890	1.0944	0.2955
smoking	2	0	0	.	.	.
physically_active	0	1	0.9936	0.3843	6.6843	0.0097
physically_active	1	0	0	.	.	.
eGFR_EPI		1	-0.00709	0.00694	1.0437	0.3070
DM_duration		1	0.0920	0.0255	12.9706	0.0003

Odds Ratio Estimates

Effect	Point Estimate	95% Wald Confidence Limits	
age	1.037	0.999	1.077
sex 1 vs 2	1.593	0.658	3.856
BMI	1.034	0.944	1.133
hypertension	0.667	0.317	1.405
logLDL_C	0.603	0.226	1.611
HbA1c	1.219	0.995	1.494
alcohol 0 vs 2	1.650	0.606	4.492
alcohol 1 vs 2	2.719	0.515	14.352
smoking 0 vs 2	1.108	0.420	2.919
smoking 1 vs 2	0.540	0.170	1.713
physically_active 0 vs 1	2.701	1.272	5.736
eGFR_EPI	0.993	0.980	1.007
DM_duration	1.096	1.043	1.153

Association of Predicted Probabilities and Observed Responses

**Percent Concordant**	78.7	**Somers' D**	0.576
**Percent Discordant**	21.1	**Gamma**	0.577
**Percent Tied**	0.2	**Tau-a**	0.286
**Pairs**	11858	**c**	0.788

model3 for leptin

The LOGISTIC Procedure

Model Information

**Data Set**	SASUSER.SUN_30DIA1
**Response Variable**	dspn_y
**Number of Response Levels**	2
**Model**	binary logit
**Optimization Technique**	Fisher's scoring

**Number of Observations Read**	219
**Number of Observations Used**	219

Response Profile

Ordered Value	dspn_y	Total Frequency
**1**	0	121
**2**	1	98

Probability modeled is dspn_y=1.

Class Level Information

Class	Value	Design Variables		
**lipid_lowering**	**0**	1	0	
	**1**	0	1	
**sex**	**1**	1	0	
	**2**	0	1	
**alcohol**	**0**	1	0	0
	**1**	0	1	0
	**2**	0	0	1
**smoking**	**0**	1	0	0
	**1**	0	1	0
	**2**	0	0	1
**physically_active**	**0**	1	0	
	**1**	0	1	

Model Convergence Status

Convergence criterion (GCONV=1E-8) satisfied.

Model Fit Statistics

Criterion	Intercept Only	Intercept and Covariates
**AIC**	303.178	277.360
**SC**	306.568	334.974
**-2 Log L**	301.178	243.360

Testing Global Null Hypothesis: BETA=0

Test	Chi-Square	DF	Pr > ChiSq
**Likelihood Ratio**	57.8183	16	<.0001
**Score**	51.2154	16	<.0001
**Wald**	39.4282	16	0.0009

Type 3 Analysis of Effects

Effect	DF	Wald Chi-Square	Pr > ChiSq
**Leptin**	1	1.7451	0.1865
**Leptin*lipid_lowerin**	1	0.9439	0.3313
**lipid_lowering**	1	0.2907	0.5898
**age**	1	3.6991	0.0544
**sex**	1	2.3703	0.1237
**BMI**	1	0.0006	0.9808
**hypertension**	1	1.3789	0.2403
**logLDL_C**	1	0.7409	0.3894
**HbA1c**	1	5.0363	0.0248
**alcohol**	2	0.8922	0.6401
**smoking**	2	2.2907	0.3181
**physically_active**	1	5.5964	0.0180
**eGFR_EPI**	1	1.0866	0.2972
**DM_duration**	1	11.4749	0.0007

Analysis of Maximum Likelihood Estimates

Parameter		DF	Estimate	Standard Error	Wald Chi-Square	Pr > ChiSq
**Intercept**		1	-5.5278	2.3303	5.6271	0.0177
**Leptin**		1	0.0370	0.0247	2.2457	0.1340
**Leptin*lipid_lowerin**	**0**	1	-0.0263	0.0271	0.9439	0.3313
**Leptin*lipid_lowerin**	**1**	0	0	.	.	.
**lipid_lowering**	**0**	1	-0.2628	0.4875	0.2907	0.5898

Analysis of Maximum Likelihood Estimates

Parameter		DF	Estimate	Standard Error	Wald Chi-Square	Pr > ChiSq
lipid_lowering	1	0	0	.	.	.
age		1	0.0369	0.0192	3.6991	0.0544
sex	1	1	0.7634	0.4958	2.3703	0.1237
sex	2	0	0	.	.	.
BMI		1	0.00128	0.0529	0.0006	0.9808
hypertension		1	-0.4439	0.3780	1.3789	0.2403
logLDL_C		1	-0.4316	0.5014	0.7409	0.3894
HbA1c		1	0.2287	0.1019	5.0363	0.0248
alcohol	0	1	0.4170	0.5087	0.6720	0.4124
alcohol	1	1	0.5873	0.7918	0.5501	0.4583
alcohol	2	0	0	.	.	.
smoking	0	1	0.2259	0.5030	0.2016	0.6534
smoking	1	1	-0.6438	0.5900	1.1907	0.2752
smoking	2	0	0	.	.	.
physically_active	0	1	0.9079	0.3838	5.5964	0.0180
physically_active	1	0	0	.	.	.
eGFR_EPI		1	-0.00723	0.00693	1.0866	0.2972
DM_duration		1	0.0872	0.0258	11.4749	0.0007

Odds Ratio Estimates

Effect	Point Estimate	95% Wald Confidence Limits	
age	1.038	0.999	1.077
sex 1 vs 2	2.145	0.812	5.670
BMI	1.001	0.903	1.111
hypertension	0.642	0.306	1.346
logLDL_C	0.649	0.243	1.735
HbA1c	1.257	1.029	1.535
alcohol 0 vs 2	1.517	0.560	4.112
alcohol 1 vs 2	1.799	0.381	8.493
smoking 0 vs 2	1.253	0.468	3.360
smoking 1 vs 2	0.525	0.165	1.670
physically_active 0 vs 1	2.479	1.168	5.260
eGFR_EPI	0.993	0.979	1.006
DM_duration	1.091	1.037	1.148

Association of Predicted Probabilities and Observed Responses

**Percent Concordant**	77.9	**Somers' D**	0.560
**Percent Discordant**	22.0	**Gamma**	0.560
**Percent Tied**	0.1	**Tau-a**	0.278
**Pairs**	11858	**c**	0.780

The LOGISTIC Procedure

Model Information

**Data Set**	SASUSER.SUN_30DIA1
**Response Variable**	dspn_y
**Number of Response Levels**	2
**Model**	binary logit
**Optimization Technique**	Fisher's scoring

**Number of Observations Read**	219
**Number of Observations Used**	219

Response Profile

Ordered Value	dspn_y	Total Frequency
**1**	0	121
**2**	1	98

Probability modeled is dspn_y=1.

Class Level Information

Class	Value	Design Variables		
**lipid_lowering**	**0**	1	0	
	**1**	0	1	
**sex**	**1**	1	0	
	**2**	0	1	
**alcohol**	**0**	1	0	0
	**1**	0	1	0
	**2**	0	0	1
**smoking**	**0**	1	0	0
	**1**	0	1	0
	**2**	0	0	1
**physically_active**	**0**	1	0	
	**1**	0	1	

Model Convergence Status

Convergence criterion (GCONV=1E-8) satisfied.

Model Fit Statistics

Criterion	Intercept Only	Intercept and Covariates
**AIC**	303.178	277.170
**SC**	306.568	334.784
**-2 Log L**	301.178	243.170

Testing Global Null Hypothesis: BETA=0

Test	Chi-Square	DF	Pr > ChiSq
**Likelihood Ratio**	58.0087	16	<.0001
**Score**	51.0369	16	<.0001
**Wald**	39.5351	16	0.0009

Type 3 Analysis of Effects

Effect	DF	Wald Chi-Square	Pr > ChiSq
**logtnfa**	1	1.1331	0.2871
**logtnfa*lipid_loweri**	1	1.1284	0.2881
**lipid_lowering**	1	0.1432	0.7051
**age**	1	4.2026	0.0404
**sex**	1	1.1751	0.2784
**BMI**	1	0.5952	0.4404
**hypertension**	1	1.3974	0.2372
**logLDL_C**	1	1.6061	0.2050
**HbA1c**	1	4.8503	0.0276
**alcohol**	2	1.1203	0.5711
**smoking**	2	2.0420	0.3602
**physically_active**	1	6.3471	0.0118
**eGFR_EPI**	1	1.3899	0.2384
**DM_duration**	1	14.1030	0.0002

Analysis of Maximum Likelihood Estimates

Parameter		DF	Estimate	Standard Error	Wald Chi-Square	Pr > ChiSq
**Intercept**		1	-5.8940	2.3916	6.0737	0.0137
**logtnfa**		1	0.00609	0.2361	0.0007	0.9794
**logtnfa*lipid_loweri**	**0**	1	-0.3399	0.3199	1.1284	0.2881
**logtnfa*lipid_loweri**	**1**	0	0	.	.	.
**lipid_lowering**	**0**	1	0.3504	0.9260	0.1432	0.7051

Analysis of Maximum Likelihood Estimates

Parameter		DF	Estimate	Standard Error	Wald Chi-Square	Pr > ChiSq
lipid_lowering	1	0	0	.	.	.
age		1	0.0393	0.0192	4.2026	0.0404
sex	1	1	0.4853	0.4477	1.1751	0.2784
sex	2	0	0	.	.	.
BMI		1	0.0357	0.0463	0.5952	0.4404
hypertension		1	-0.4487	0.3796	1.3974	0.2372
logLDL_C		1	-0.6305	0.4975	1.6061	0.2050
HbA1c		1	0.2269	0.1030	4.8503	0.0276
alcohol	0	1	0.4009	0.5174	0.6005	0.4384
alcohol	1	1	0.7735	0.8200	0.8898	0.3455
alcohol	2	0	0	.	.	.
smoking	0	1	0.1220	0.4930	0.0612	0.8045
smoking	1	1	-0.6695	0.5858	1.3064	0.2531
smoking	2	0	0	.	.	.
physically_active	0	1	0.9657	0.3833	6.3471	0.0118
physically_active	1	0	0	.	.	.
eGFR_EPI		1	-0.00812	0.00689	1.3899	0.2384
DM_duration		1	0.0967	0.0257	14.1030	0.0002

Odds Ratio Estimates

Effect	Point Estimate	95% Wald Confidence Limits	
age	1.040	1.002	1.080
sex 1 vs 2	1.625	0.676	3.907
BMI	1.036	0.946	1.135
hypertension	0.638	0.303	1.343
logLDL_C	0.532	0.201	1.411
HbA1c	1.255	1.025	1.535
alcohol 0 vs 2	1.493	0.542	4.116
alcohol 1 vs 2	2.167	0.434	10.814
smoking 0 vs 2	1.130	0.430	2.969
smoking 1 vs 2	0.512	0.162	1.614
physically_active 0 vs 1	2.627	1.239	5.568
eGFR_EPI	0.992	0.979	1.005
DM_duration	1.102	1.047	1.159

Association of Predicted Probabilities and Observed Responses

**Percent Concordant**	78.4	**Somers' D**	0.569
**Percent Discordant**	21.5	**Gamma**	0.570
**Percent Tied**	0.1	**Tau-a**	0.283
**Pairs**	11858	**c**	0.784

model3 for loghsCRP

The LOGISTIC Procedure

Model Information

**Data Set**	SASUSER.SUN_30DIA1
**Response Variable**	dspn_y
**Number of Response Levels**	2
**Model**	binary logit
**Optimization Technique**	Fisher's scoring

**Number of Observations Read**	219
**Number of Observations Used**	219

Response Profile

Ordered Value	dspn_y	Total Frequency
**1**	0	121
**2**	1	98

Probability modeled is dspn_y=1.

Class Level Information

Class	Value	Design Variables		
**lipid_lowering**	**0**	1	0	
	**1**	0	1	
**sex**	**1**	1	0	
	**2**	0	1	
**alcohol**	**0**	1	0	0
	**1**	0	1	0
	**2**	0	0	1
**smoking**	**0**	1	0	0
	**1**	0	1	0
	**2**	0	0	1
**physically_active**	**0**	1	0	
	**1**	0	1	

Model Convergence Status

Convergence criterion (GCONV=1E-8) satisfied.

Model Fit Statistics

Criterion	Intercept Only	Intercept and Covariates
**AIC**	303.178	271.843
**SC**	306.568	329.458
**-2 Log L**	301.178	237.843

Testing Global Null Hypothesis: BETA=0

Test	Chi-Square	DF	Pr > ChiSq
**Likelihood Ratio**	63.3351	16	<.0001
**Score**	55.2594	16	<.0001
**Wald**	42.0754	16	0.0004

Type 3 Analysis of Effects

Effect	DF	Wald Chi-Square	Pr > ChiSq
**loghsCRP**	1	7.5372	0.0060
**loghsCRP*lipid_lower**	1	0.9852	0.3209
**lipid_lowering**	1	1.4935	0.2217
**age**	1	3.1393	0.0764
**sex**	1	1.0287	0.3105
**BMI**	1	1.0431	0.3071
**hypertension**	1	0.3841	0.5354
**logLDL_C**	1	0.8501	0.3565
**HbA1c**	1	7.6597	0.0056
**alcohol**	2	2.2152	0.3303
**smoking**	2	2.0953	0.3508
**physically_active**	1	7.7810	0.0053
**eGFR_EPI**	1	4.0052	0.0454
**DM_duration**	1	12.3797	0.0004

Analysis of Maximum Likelihood Estimates

Parameter		DF	Estimate	Standard Error	Wald Chi-Square	Pr > ChiSq
**Intercept**		1	-6.4361	2.3195	7.6996	0.0055
**loghsCRP**		1	-0.6987	0.2857	5.9824	0.0144
**loghsCRP*lipid_lower**	**0**	1	0.3348	0.3374	0.9852	0.3209
**loghsCRP*lipid_lower**	**1**	0	0	.	.	.
**lipid_lowering**	**0**	1	-0.4768	0.3901	1.4935	0.2217

Analysis of Maximum Likelihood Estimates

Parameter		DF	Estimate	Standard Error	Wald Chi-Square	Pr > ChiSq
**lipid_lowering**	**1**	0	0	.	.	.
**age**		1	0.0339	0.0191	3.1393	0.0764
**sex**	**1**	1	0.4572	0.4508	1.0287	0.3105
**sex**	**2**	0	0	.	.	.
**BMI**		1	0.0494	0.0483	1.0431	0.3071
**hypertension**		1	-0.2411	0.3891	0.3841	0.5354
**logLDL_C**		1	-0.4572	0.4959	0.8501	0.3565
**HbA1c**		1	0.3061	0.1106	7.6597	0.0056
**alcohol**	**0**	1	0.6506	0.5235	1.5445	0.2140
**alcohol**	**1**	1	1.0279	0.8513	1.4579	0.2273
**alcohol**	**2**	0	0	.	.	.
**smoking**	**0**	1	0.1334	0.5043	0.0699	0.7914
**smoking**	**1**	1	-0.6754	0.6005	1.2649	0.2607
**smoking**	**2**	0	0	.	.	.
**physically_active**	**0**	1	1.1264	0.4038	7.7810	0.0053
**physically_active**	**1**	0	0	.	.	.
**eGFR_EPI**		1	-0.0149	0.00746	4.0052	0.0454
**DM_duration**		1	0.0910	0.0259	12.3797	0.0004

Odds Ratio Estimates

Effect	Point Estimate	95% Wald Confidence Limits	
**age**	1.034	0.996	1.074
**sex 1 vs 2**	1.580	0.653	3.822
**BMI**	1.051	0.956	1.155
**hypertension**	0.786	0.367	1.684
**logLDL_C**	0.633	0.240	1.673
**HbA1c**	1.358	1.093	1.687
**alcohol 0 vs 2**	1.917	0.687	5.348
**alcohol 1 vs 2**	2.795	0.527	14.828
**smoking 0 vs 2**	1.143	0.425	3.070
**smoking 1 vs 2**	0.509	0.157	1.651
**physically_active 0 vs 1**	3.085	1.398	6.806
**eGFR_EPI**	0.985	0.971	1.000
**DM_duration**	1.095	1.041	1.152

Association of Predicted Probabilities and Observed Responses

**Percent Concordant**	79.6	**Somers' D**	0.594
**Percent Discordant**	20.2	**Gamma**	0.595
**Percent Tied**	0.1	**Tau-a**	0.295
**Pairs**	11858	**c**	0.797

model3 for logl2n

The LOGISTIC Procedure

Model Information

**Data Set**	SASUSER.SUN_30DIA1
**Response Variable**	dspn_y
**Number of Response Levels**	2
**Model**	binary logit
**Optimization Technique**	Fisher's scoring

**Number of Observations Read**	219
**Number of Observations Used**	219

Response Profile

Ordered Value	dspn_y	Total Frequency
**1**	0	121
**2**	1	98

Probability modeled is dspn_y=1.

Class Level Information

Class	Value	Design Variables		
**lipid_lowering**	**0**	1	0	
	**1**	0	1	
**sex**	**1**	1	0	
	**2**	0	1	
**alcohol**	**0**	1	0	0
	**1**	0	1	0
	**2**	0	0	1
**smoking**	**0**	1	0	0
	**1**	0	1	0
	**2**	0	0	1
**physically_active**	**0**	1	0	
	**1**	0	1	

Model Convergence Status

Convergence criterion (GCONV=1E-8) satisfied.

Model Fit Statistics

Criterion	Intercept Only	Intercept and Covariates
AIC	303.178	278.786
SC	306.568	336.401
-2 Log L	301.178	244.786

Testing Global Null Hypothesis: BETA=0

Test	Chi-Square	DF	Pr > ChiSq
Likelihood Ratio	56.3920	16	<.0001
Score	50.0541	16	<.0001
Wald	39.1040	16	0.0011

Type 3 Analysis of Effects

Effect	DF	Wald Chi-Square	Pr > ChiSq
logl2n	1	0.0931	0.7603
logl2n*lipid_lowerin	1	0.9277	0.3355
lipid_lowering	1	0.5314	0.4660
age	1	3.7985	0.0513
sex	1	1.1122	0.2916
BMI	1	0.4733	0.4915
hypertension	1	1.5941	0.2067
logLDL_C	1	1.3306	0.2487
HbA1c	1	4.6342	0.0313
alcohol	2	0.9461	0.6231
smoking	2	1.9376	0.3795
physically_active	1	5.9439	0.0148
eGFR_EPI	1	1.5242	0.2170
DM_duration	1	13.6708	0.0002

Analysis of Maximum Likelihood Estimates

Parameter		DF	Estimate	Standard Error	Wald Chi-Square	Pr > ChiSq
Intercept		1	-6.3002	3.0273	4.3312	0.0374
logl2n		1	0.1598	0.3655	0.1910	0.6621
logl2n*lipid_lowerin	0	1	-0.4697	0.4877	0.9277	0.3355
logl2n*lipid_lowerin	1	0	0	.	.	.
lipid_lowering	0	1	1.7060	2.3403	0.5314	0.4660

Analysis of Maximum Likelihood Estimates

Parameter		DF	Estimate	Standard Error	Wald Chi-Square	Pr > ChiSq
lipid_lowering	1	0	0	.	.	.
age		1	0.0370	0.0190	3.7985	0.0513
sex	1	1	0.4684	0.4441	1.1122	0.2916
sex	2	0	0	.	.	.
BMI		1	0.0319	0.0464	0.4733	0.4915
hypertension		1	-0.4792	0.3795	1.5941	0.2067
logLDL_C		1	-0.5694	0.4936	1.3306	0.2487
HbA1c		1	0.2196	0.1020	4.6342	0.0313
alcohol	0	1	0.3868	0.5184	0.5568	0.4555
alcohol	1	1	0.6816	0.8019	0.7224	0.3954
alcohol	2	0	0	.	.	.
smoking	0	1	0.1771	0.4924	0.1293	0.7191
smoking	1	1	-0.6041	0.5805	1.0830	0.2980
smoking	2	0	0	.	.	.
physically_active	0	1	0.9334	0.3829	5.9439	0.0148
physically_active	1	0	0	.	.	.
eGFR_EPI		1	-0.00860	0.00697	1.5242	0.2170
DM_duration		1	0.0948	0.0256	13.6708	0.0002

Odds Ratio Estimates

Effect	Point Estimate	95% Wald Confidence Limits	
age	1.038	1.000	1.077
sex 1 vs 2	1.597	0.669	3.814
BMI	1.032	0.943	1.131
hypertension	0.619	0.294	1.303
logLDL_C	0.566	0.215	1.489
HbA1c	1.246	1.020	1.521
alcohol 0 vs 2	1.472	0.533	4.067
alcohol 1 vs 2	1.977	0.411	9.520
smoking 0 vs 2	1.194	0.455	3.133
smoking 1 vs 2	0.547	0.175	1.705
physically_active 0 vs 1	2.543	1.201	5.386
eGFR_EPI	0.991	0.978	1.005
DM_duration	1.099	1.046	1.156

Association of Predicted Probabilities and Observed Responses

**Percent Concordant**	77.6	**Somers' D**	0.554
**Percent Discordant**	22.2	**Gamma**	0.554
**Percent Tied**	0.2	**Tau-a**	0.275
**Pairs**	11858	**c**	0.777
